# Unravelling the Enantioselective Mechanism of Benzylsuccinate Synthase: Insights into Anaerobic Hydrocarbon Degradation through Multiscale Modeling and Microkinetics

**DOI:** 10.1021/acs.biochem.5c00699

**Published:** 2026-03-03

**Authors:** Maciej Szaleniec, Gabriela Oleksy, Tomasz Borowski, Johann Heider

**Affiliations:** † Jerzy Haber Institute of Catalysis and Surface Chemistry, Polish Academy of Sciences, Kraków 30-239, Poland; ‡ Department of Biology, Laboratory for Microbial Biochemistry, 9377 Philipps University Marburg, 35043 Marburg, Germany; § SYNMIKRO Research Center, 35043 Marburg, Germany

## Abstract

Fumarate-adding enzymes (FAE) are a subset of the glycyl radical enzyme superfamily involved in anaerobic hydrocarbon degradation. Benzylsuccinate synthase (BSS) catalyzes the enantiospecific formation of *R*-benzylsuccinate from toluene and fumarate, initiating anaerobic toluene degradation. In this paper, we present a detailed theoretical study of the reaction mechanism using classical molecular dynamics and multiscale modeling (QM/MM). We describe the potential energy surface of the reaction and confirm the previously postulated mechanism. However, the multiscale character of our model allowed us to elucidate the origins of several experimentally observed catalytic phenomena, such as the inversion of the benzylic carbon configuration upon C–C bond formation and the *syn* addition of the abstracted H atom back to the benzylsuccinyl radical. The obtained model is supported by microkinetic analysis and was able to explain and quantitatively predict the strict *R*-enantioselectivity of BSS, which is enforced predominantly by the dynamic kinetic behavior of toluene in the active site, leading to over 40-times faster production of the *R*-enantiomer, not by the binding orientation of the fumarate. Our study contributes to the elucidation of the catalytic processes catalyzed by BSS and its role in the bioremediation of hydrocarbon pollutants.

## Graphical abstract

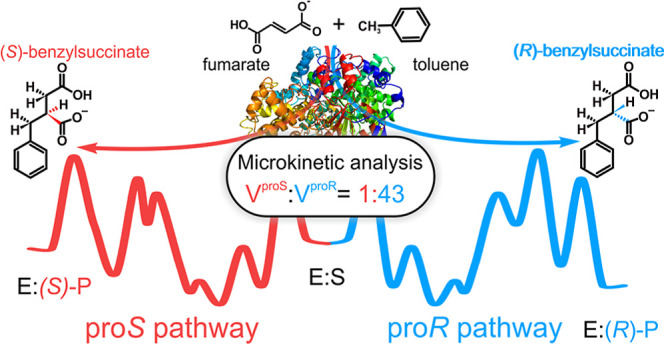

## Introduction

Fumarate-adding enzymes (FAE) comprise a subset of the glycyl radical enzyme (GRE) superfamily,^1,2^ which are involved in anaerobic degradation pathways of hydrocarbons or similar substrates and catalyze the addition of chemically inert alkyl carbon atoms to the double bond of a fumarate cosubstrate.^3–6^ The first recognized FAE was benzylsuccinate synthase (BSS),^7,8^ which enantiospecifically forms (*R*)-benzylsuccinate from toluene and fumarate^8–10^ and initiates anaerobic toluene degradation in all known organisms capable of this trait (Figure 1B). Very similar initial reactions were also reported for the anaerobic degradation of xylene and cresol isomers,^11–13^ as well as for 2-methylnaphthalene,^14,15^ ethylbenzene,^16^ and even aliphatic alkanes^17–19^ or cycloalkanes.^20^ The discovery of both the characteristic succinate adducts as well as the genes coding for the respective FAE in field studies of contaminated sediments or aquifers indicated that these enzymes are widespread in nature and contribute an essential part in bioremediation situations.^21–26^

Like other GREs, the FAE members contain a large subunit of approximately 100 kDa, which forms the active site (Figure 1A) and contains a conserved glycine close to the C-terminus (Gly829, to be activated to a glycyl radical) and a conserved cysteine at about the middle of the primary sequence (Cys493).^7^ Although the similarity with other GREs outside the functional centers is limited, the available X-ray structure information on BSS shows very good structural conservation with other members of the GRE.^27,28^ In contrast to the other known GRE, BSS, and all related aromatic-activating FAE, contains two additional small subunits, which are encoded by separate genes (*bssBC*) in a common operon with the *bssA* gene for the large subunit. The alkane-activating FAE may even be expected to contain three small subunits in addition to the large glycyl-radical containing one, judging from the presence of an additional gene in the respective operons.^17^ These small subunits contain unusual Fe_4_S_4_ clusters with low redox potential^28,29^ and appear to be necessary for enzyme activity,^3,30^ and stabilization of the thiyl radical state.^31^ Like all GRE, BSS and the other FAE need to be activated to the active glycyl-radical status by an activating enzyme, which is usually encoded in the same operons containing the genes for the subunits (*bssD* in the case of BSS), or in close vicinity to the other *bss* genes (e.g., separated by a gene coding for a transposase in the operons of some alkane degraders^17^). The activating enzymes belong to the large family of S-adenosylmethionine (SAM)-dependent radical enzymes and abstract a hydrogen atom from the specifically recognized conserved Gly residue of the large subunit to convert the enzyme into the catalytically active glycyl radical form. Like all SAM-radical enzymes, these activating enzymes carry an Fe_4_S_4_ cluster in their N-terminal domains, which contains a bound SAM molecule that can be converted to methionine and an adenosyl radical by a one-electron reduction via the Fe_4_S_4_ cluster. The generated adenosyl radical then reacts with the active site glycine of the GRE and abstracts the pro­(*S*) hydrogen atom, while being reduced to 5′-deoxyadenosine.^32–40^ The glycyl radical in activated GRE is involved in mesomeric interchange with the electrons of the peptide bond and, therefore, relatively stable. However, exposure to oxygen results in irreversible destruction of the GRE by oxygenolytic cleavage of the peptide bond at the site of the glycyl radical.^7,41^ The introduced glycyl radicals in GRE are continuously recycled during the reaction cycles, which allows many turnovers without reactivation, instead of requiring the energetically expensive degradation and regeneration of SAM for every reaction round.^39,41,42^

The mechanism of BSS was initially proposed on the basis of what was generally known for GRE enzymes.^3,6,43^ The glycyl radical represents a stable state of the activated enzyme but is not very reactive. Therefore, it is assumed that the substrates toluene and fumarate bind to the enzyme in this state, followed by closing the active site.^30,44^ The reaction is then initiated by the transfer of a hydrogen atom (HAT) from the conserved Cys493 of the active site to the glycyl radical, generating a reactive thiyl radical, which then abstracts a hydrogen atom from the methyl group of toluene. The generated benzyl radical intermediate attacks the double bond of the bound fumarate, forming a new C–C bond in a benzylsuccinyl product radical. Finally, the product radical reabstracts the hydrogen atom from the side chain of Cys493, and the resulting thiyl radical abstracts the hydrogen from Gly829. This re-establishes the stable glycyl radical form, allowing the enzyme to open the active site for product release and subsequent substrate binding. This mechanism was originally suggested based on results of calculations for a gas phase model, which did not take into account the presence of the enzyme or any other catalyst.^45^ Later on, we expanded this analysis to a cluster model based on the X-ray structure of the BSS apoenzyme (PDB 4PKF). This model allowed for describing a continuous mechanism from the first thiyl radical state to benzylsuccinate formation, but fell short of accurately describing the whole reaction.^44^ In addition to lacking the transition of the glycyl radical to the thiyl radical state, it disregarded the conformational changes associated with fumarate binding, evidence for which was published only at a later stage.^27^

Therefore, in this study we refine our understanding of the catalytic process and present results obtained with the first QM/MM model that was based on the complete structure of the active subunit crystallized with the bound substrate (PDB 5BWD). The results cover the whole reaction cycle. We obtained new insights into the geometry of the transition states that are shaped by the steric constraints imposed by the protein structure. In addition, we analyzed the progress of the reaction by using microkinetic analysis and identified factors by which the enzyme imposes the regioselectivity and enantioselectivity of the C–C bond formation.

## Methods

### BSS Model Preparation

The initial structure of the α subunit of BSS_T1_ in complex with monoprotonated fumarate and toluene was obtained from the crystal structure (PDB codes: 5BWD, 5BWE)^27^ as described previously by Salii et al.^30^ and Szaleniec et al.^46^ The UniProt identification of the catalytic alpha subunit of BSS_T1_ is O68395. The selection of the starting geometry for QM/MM modeling was conducted by means of the clustering of the MD trajectory using the k-means method and taking into consideration the heavy atoms of the active site residues (see Supporting Information). Then, from the most abundant cluster, representing 36.6% of the trajectory, the frame with the shortest distance between the methyl carbon of toluene and the S atom of Cys493 was selected, and its geometry was minimized with the AMBER force field (ff03.r1). The monoanionic state of the fumarate was selected based on the results of the docking studies presented in^44^ and is consistent with a recently published investigation of the initial phase of the reaction.^46^ Unexpectedly, modeling the whole reaction pathway with completely deprotonated fumarate proved extremely challenging due to problems with the geometry convergence of such QM/MM models, even when an extended QM part was used.

### MD Simulations

All classical MD simulations were performed for BSS models using the AMBER ff03.r1 force field.^47^ The calculations were conducted using AMBER 18–22 ^48^ according to the previously described protocol^30^ (see Supporting Information for details). The following MD simulations were conducted for the study: four 100 ns simulations for BSS with radical Gly829 in complex with toluene and monoprotonated fumarate in the pro*R* orientation (Figure S1), three 100 ns simulations for BSS with radical Gly829 in complex with toluene and monoprotonated fumarate in the pro*S* orientation (Figure S2), four 100 ns simulations of BSS with radical Cys493 in complex with toluene and monoprotonated fumarate in the pro*R* orientation (Figure S3), two 100 ns simulations of radical Cys493 and monoprotonated benzylsuccinate (protonation at carboxyl group close to Arg508) (Figure S4), as well as two 100 ns simulations for BSS with radical Gly829 in complex with monoprotonated fumarate in the pro*R* orientation (Figure S5A,B), the pro*R* orientation with the protonated group rotated by 180° (Figure S5C,D) and the pro*S* bound orientation (Figure S5E,F). The selection of simulation regions for statistical analysis was based on the stability of the RMSD calculated for the protein backbone, and the relevance of the selection was further confirmed by analysis of the RMSD calculated for the active-site atoms. The reagent binding Δ*G*_b_ values were calculated using the MM/PBSA protocol^49^ using the Poisson-Boltzmann and Generalized Born approach (Figure S16).

**Figure 1:**
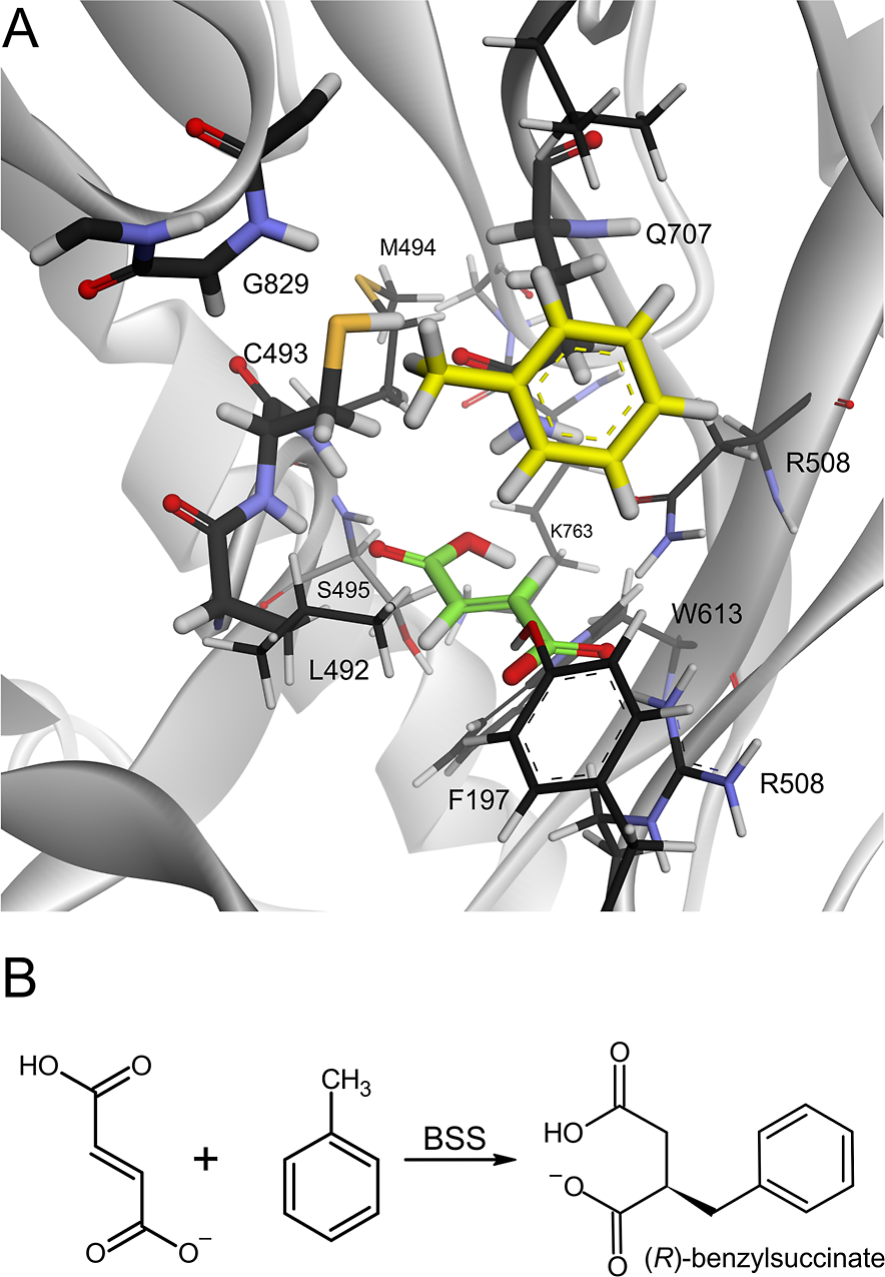
(A) BSS active site, (B) scheme of the reaction. The C atoms of toluene are depicted in yellow, those of fumarate in green.

### QM/MM

All QM/MM calculations were conducted using the Gaussian16 program.^50^ The QM/MM model obtained from MD simulations was stripped of all seven sodium ions and most of the water molecules, leaving only H_2_O molecules penetrating a 20 Å radius from Cys493. The positions of all residues and water molecules outside a 15 Å radius from Cys493 were frozen in geometry optimization. The latter was done to ensure faster geometry optimization and to increase the likelihood of the model staying in the same basin of attraction.^51^ The fumarate was modeled in the monoprotonated state, following previous docking studies,^44^ and the model’s overall charge was −7. Two sizes of high layer (HL) were used for the QM portion of the study: a small one (QM1, Figure 2a) used for geometry optimization and vibrational analysis, or a big one (QM2, Figure 2b), used for single point correction of the energy. The QM1 comprised Cys493 and Gly829 residues with adjacent fragments of the main chain, Gln707, fumarate, and toluene. The QM2 comprised QM1 extended by all residues and solvent molecules penetrating a 3 Å radius of Cys493, toluene, and fumarate (330 atoms). The charge of both models was −1. All calculations were conducted for a doublet spin state because of the presence of a single radical.

**Figure 2:**
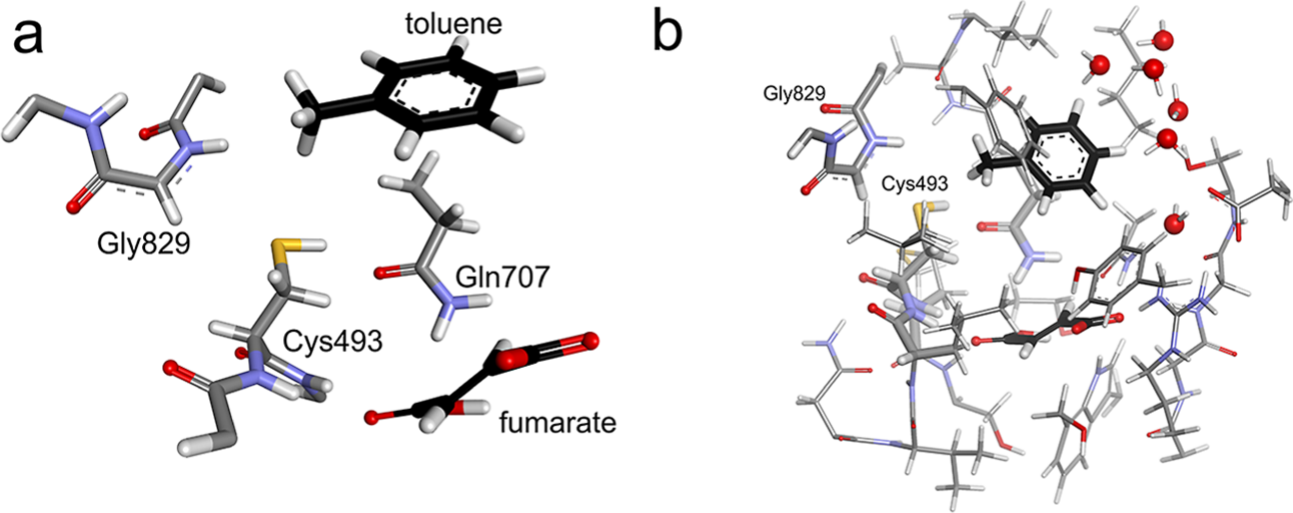
QM (HL) part of the QM/MM BSS models: (a) a small one (QM1) used for geometry optimization and vibrational corrections, and (b) a big one (QM2) used for single-point energy corrections.

The geometry of the QM/MM models was optimized at the unrestricted B3LYP/6–31g­(d,p):AMBER level of theory, using an electronic embedding approach,^52^ which was followed by vibrational analysis, introducing vibrational corrections to the energy of the stationary points. For each elementary step, the geometry of the model was settled in the local minimum by reoptimization of the model at the stationary states adjacent to the TS under investigation. The procedure was repeated up to 3 times until the energy of the system converged. The transition states were localized using relaxed scans along the reaction coordinate (e.g., d­(Cys–S–H···C^rad^-Gly)) followed by TS optimization using the Berny algorithm. After obtaining the geometry of TS, each stationary state preceding or following a particular TS was optimized individually, with initial geometries derived from an intrinsic reaction coordinate (IRC) scan. For each of the stationary states, vibrational analysis was conducted to confirm that a minimum or a first-order saddle point (TS) was reached. The inevitable discontinuities in the potential energy surface along the multistep reaction, which originated from changes in residue conformation(s) due to reagent movement during the reaction scans or multiple reoptimizations of stationary points before TS optimization, were corrected by applying reference calculations (i.e., calculation of the particular HL geometry in the new conformation of the MM part, see Tables S4 and S5). The energies of the final stationary points were corrected with single point calculations using QM2 at unrestricted B3LYP/6–311g+(2d,2p):AMBER level of theory with electronic embedding and Grimme D3 corrections for dispersion interactions.^53^ The electronic energies were corrected with zero-point energies calculated at default conditions (1 atm., 298.15 K, no scaling factor—see Tables S1 and S2). For predicting reaction rates, Gibbs free energy corrections were used, obtained at 1 atm., 303 K, with a 0.9806 scaling factor (Tables S3 and S7). All energies presented in the study are in kJ mol^–1^.

### Kinetic Rate Estimations

All kinetic constants were calculated according to a standard equation from transition state theory, assuming a temperature of 303 K, and the transmission coefficient is equal to one (Tables S6 and S8). To account for the tunnelling effect that may be involved in the H atom transfer, the obtained rates were corrected by Wigner’s tunnelling corrections Γ­(*T*).^54^ The calculated kinetic constants were used to estimate the steady-state reaction rate using the equation derived according to the King-Altman algorithm^55^ (see Supporting Information methods section).

### BSS Production in *Aromatoleum evansii*

A cell-free extract of *Aromatoleum evansii* KB740 conjugants producing the recombinant BSS from *Thauera aromatica* K172 was obtained as described in Salii et al.^30^ The genetic construct contained the following genes: bssA (A0A2R4BRH7), bssB (A0A2R4BRW5), bssC (A0A2R4BRP1), and bssD (A0A2R4BRI4).

### Activity Assay and Sample Preparation for Chiral Chromatography

The activity assay of recombinant wild-type BSS, overproduced in *A. evansii*, was conducted in 10 mL reactors, comprised of 2 mL of the cell-free extract, 10 mM of fumaric acid, and buffered at pH 7.8 with 7.3 mL of 20 mM TEA-HCl. Reaction was started by adding toluene from a 100 mM isopropanol stock, to the final concentration of 2 mM. Experiments were conducted at ambient temperature. Five mL samples were collected at t_0_ and after 1 h. Reaction was stopped by the addition of 10% of H_2_SO_4_ (v/v), and the proteins were removed by centrifugation (13 000 rpm). After that, the reaction mixture was extracted with >99.8% diethyl ether and left for evaporation. Extracted reagents were resuspended in 120 μL of isopropanol.

### Normal-Phase Chiral Chromatography

The enantioselectivity of the reaction catalyzed by wild type BSS was tested with normal-phase high-performance liquid chromatography. Separation of the benzylsuccinic acid to *R*- and *S*-optical enantiomers was performed on an Agilent 1100 VL instrument, coupled with a diode array detector (DAD) at the wavelength of 210 nm. Two μL of standards or extracts of reaction mixtures were injected on a Lux-Cellulose-1 column (Phenomenex 5 μm, 10 × 100 mm) thermostated at 30 °C and separated isocratically at 1 mL/min flow rate with a mobile phase of 90:10:0.1 *n*-hexane/isopropanol: 0.1% trifluoroacetic acid. (*R*)-benzylsuccinic acid eluted first at 3.0 min, and the peak of the (*S*)-enantiomer was observed at 3.72 min.

## Results

This study was initiated to expand the knowledge of the reaction mechanism of BSS beyond our previous study, conducted on a limited cluster model.^56^ The new data allowed to start the description of the mechanism from the glycyl radical state and with a newly published structure with the bound substrates. This revealed the importance of considering conformational variants (e.g., rotamers) of active site residues to correctly assess the energetics of the reaction. Furthermore, the stereochemistry of the reaction is explained by different kinetic rates for the pathways leading to different enantiomers of the product, rather than by different regioselectivity of C–C bond formation based on the mode of fumarate binding.

It should be noted that the QM/MM study was limited to one structure representing the most prevalent conformation observed in MD simulation, mainly due to model size (2900 unrestrained atoms), calculations of five consecutive reaction steps, and the necessity to analyze several pathway variants (see below). Also, note that the substantial unrestrained part of the model and its conformational flexibility led to unavoidable discontinuities of the energy profile (i.e., the model transferring to another local minimum). These arise from the shifts of some residues during geometry optimization of the model at various stages of the reaction and usually occur in all QM/MM studies to a similar extent as observed here.^57^

### Step 1—Activation of Cys493

The first step of the reaction (Figure 4a) involves the hydrogen atom transfer (HAT) from Cys493 onto radical Gly829. As indicated by MD simulation, the conformation of the Cys493 residue is predominantly (73% of simulation time) oriented toward Gln707 with an H-bond formed between the SH group of Cys493 and the carbonyl group of Gln707, resulting in a C_α_–C_β_–S_γ_–H_δ_ dihedral angle of 240° (Figures 4b and S6). Therefore, to face toward the glycyl radical and transfer the H atom, Cys493 has first to break the H-bond stabilizing its position, by rotating along the C_β_–S_γ_ bond (from the angle of 240° to 62°) (Figure S6). However, the analysis of the MD trajectory indicates that the position of the Hγ proton is not fixed during the simulation, as it rotates quite freely along the C_β_–S_γ_ bond, as indicated by Figure S6D. After geometry optimization at the QM/MM level, the energy of the conformer facing the glycyl radical was higher by only 3.63 kJ/mol. Therefore, we did not evaluate the barrier of proton rotation.

**Figure 3:**
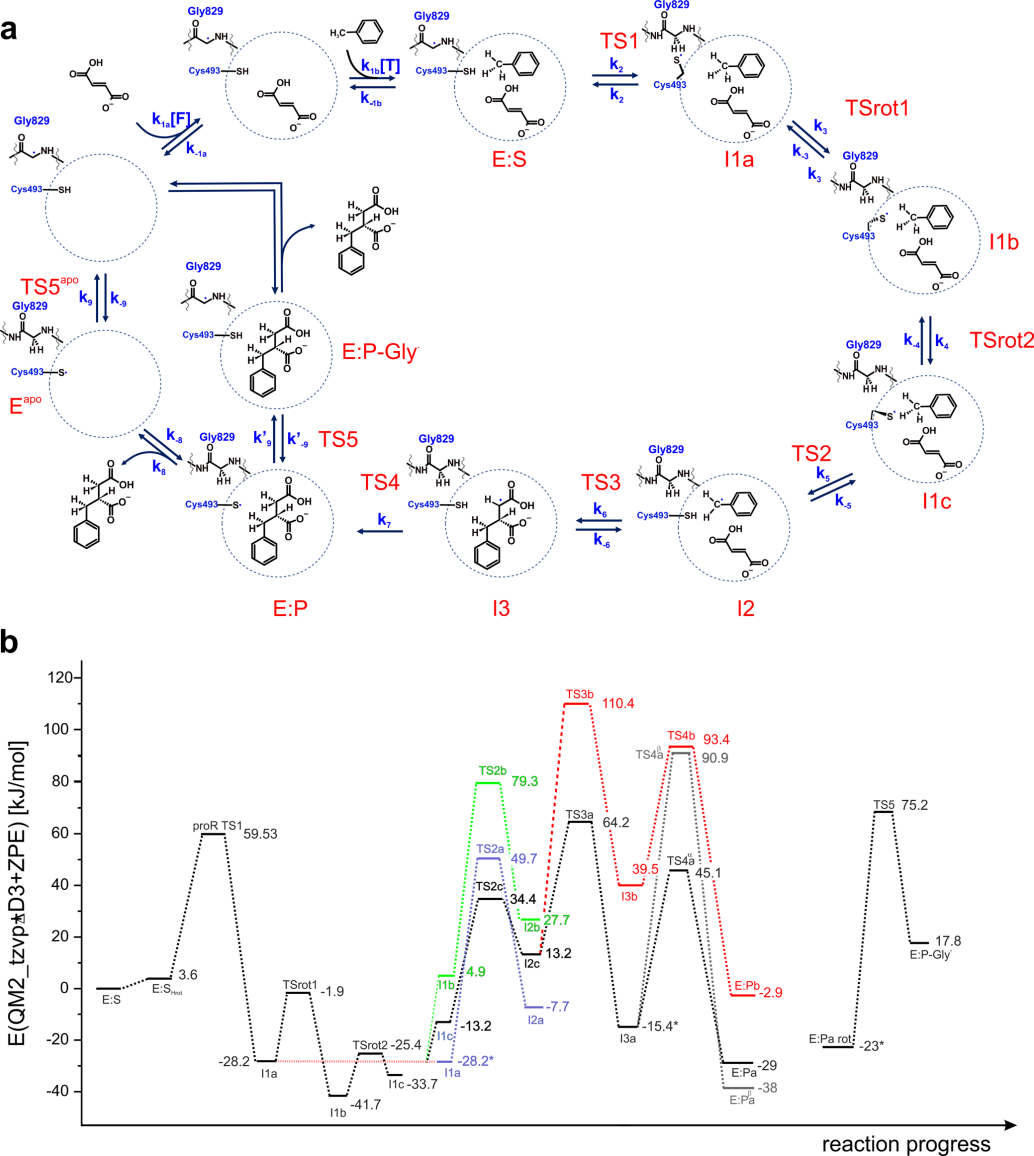
(A) The mechanism proposed for *R*-benzylsuccinate formation taking into account the current work and a recent report on HAT between glycyl radical and Cys493.^46^ (B) Energy diagram for the pro*R* conformation of the fumarate; * and dotted red line indicate the site of profile matching due to conformation changes of the model along the reaction coordinate.

**Figure 4:**
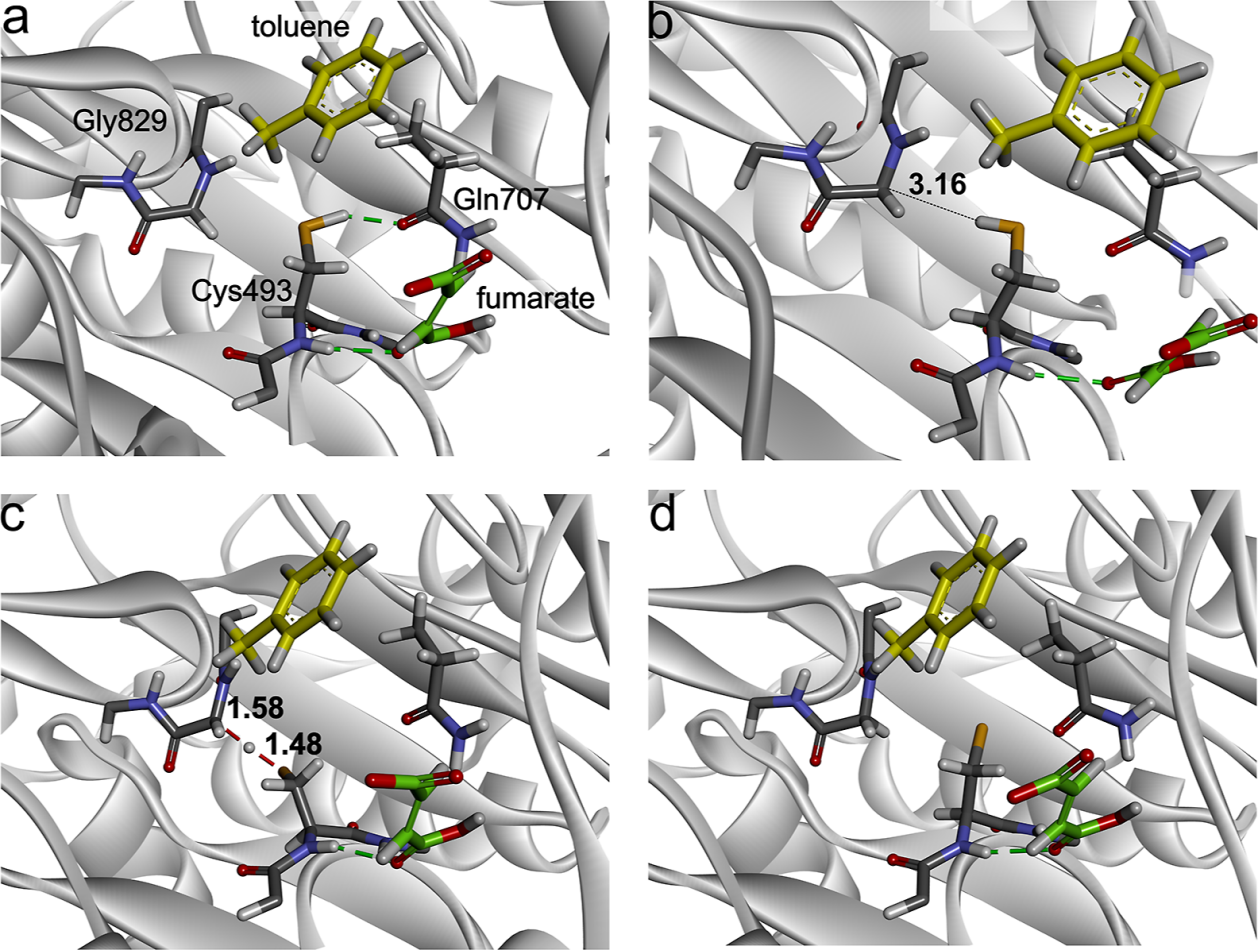
Geometry of BSS during Cys activation: (a) E:S complex, (b) E:S_Hrot_, (c) pro*R* TS1, (d) intermediate I1a. Fumarate in green, toluene in yellow.

In the next step, a reversible HAT occurs from the Cys493 SH group to the glycyl radical, as shown in Figure 4. We have recently devoted much attention to this process,^46^ showing that Cys493 can transfer the H atom from either the re or si face of the glycyl radical. While the former is associated with a lower energy barrier (59.5 kJ/mol), the latter is significantly slower but still expected to occur occasionally in the same time frame (at a barrier of 71.0 kJ/mol). In the transition state (Figure 4b), the bond lengths are 1.48 Å for S–H and 1.58 Å for C–H, while the C–H–S angle is 167° (see Table S9 for all relevant parameters). The radical spin density of the transition state (Figure S15a) is divided between the Cα of Gly829 and the S atom of Cys493 (0.66 vs 0.31, respectively).

The HAT is completed with the formation of Gly829 in the nonradical state and the formation of the (almost) entirely radical thiyl group at Cys493 (0.875 spin located at the S atom), which is calculated to be more stable than ES_Hrot_ (by 31.84 kJ/mol, Figure 3b).

Because of the removal of an H atom from the sulfhydryl group, the resulting radical cysteine has slightly higher mobility, e.g., realized as rotation along the Cα-Cβ bond. While the Cys493 is to some extent restrained before the removal of the H atom (with 73% of the snapshots oriented toward Gln707—Figure S7A), after HAT, the occupation of different conformers is more even. Although the radical placed between Gln707 and Gly829 (at an angle of 52.5°) remained the most probable conformation (69% of simulation time in 59° ± 29°), the chance of attaining alternative conformations with dihedral angles C–Cα-Cβ-Sγ of 287° ± 21° and 170° ± 29°, which both point toward the active site, became higher for the thiyl radical form (the former at 29% of simulation time, the latter at 2%; see Figure S7B). To gain insight into the energy barriers associated with the conformation changes of the radical Cys, we have optimized transition states related to the rotation along C–Cα-Cβ-Sγ dihedral angles of Cys493. First, we were able to identify the same energy minima as observed in MD simulations at similar C–Cα-Cβ-Sγ dihedral angles, namely 52.8°, 186°, and 280° (I1a, I1c, and I1b). The radical is oriented toward the active site in the two conformations with 186° or 280° dihedral angles, whereas it is directed toward the opposite side in the 52.8° conformation. The highest rotation barrier (TSrot3) is associated with the transition between the minima at 52.5° and 186°, with an activation energy of approximately 63.9 kJ/mol and a dihedral angle of 144°. The rotation barrier (TSrot1) between the minima I1a and I1b (at 52.5° and 280°) turned out to be much smaller, i.e., 26.3 kJ/mol (dihedral angle of 339°), and I1b was more stable than I1a by 13.5 kJ/mol. Finally, the rotation barrier (TSrot2) between the two minima presenting the thiyl radical toward the active site (I1b at 280° and I1c at 186°) is calculated to be 16.3 kJ/mol (dihedral 222.5°), and the final energy of I1c was 8 kJ/mol higher than that of I1b (Figures 3b and S8). This analysis shows that the radical-carrying cysteine residue will very rarely rotate in the counterclockwise rotation (i.e., between I1a and I1c), mostly due to steric hindrance introduced by the loop carrying Gly829. Instead, it will rotate clockwise, breaking away from the interaction with Gln707 at 52.5° to reach the first local minimum at 280° and finally the shallow second minimum around 186° (Figure 5). It should be noted that the rotational analysis resulted in miniscule change in enzyme conformation, which required −7.6 kJ/mol correction to match the energy of I1a and I1 obtained after TS1 (see Table S4).

**Figure 5:**
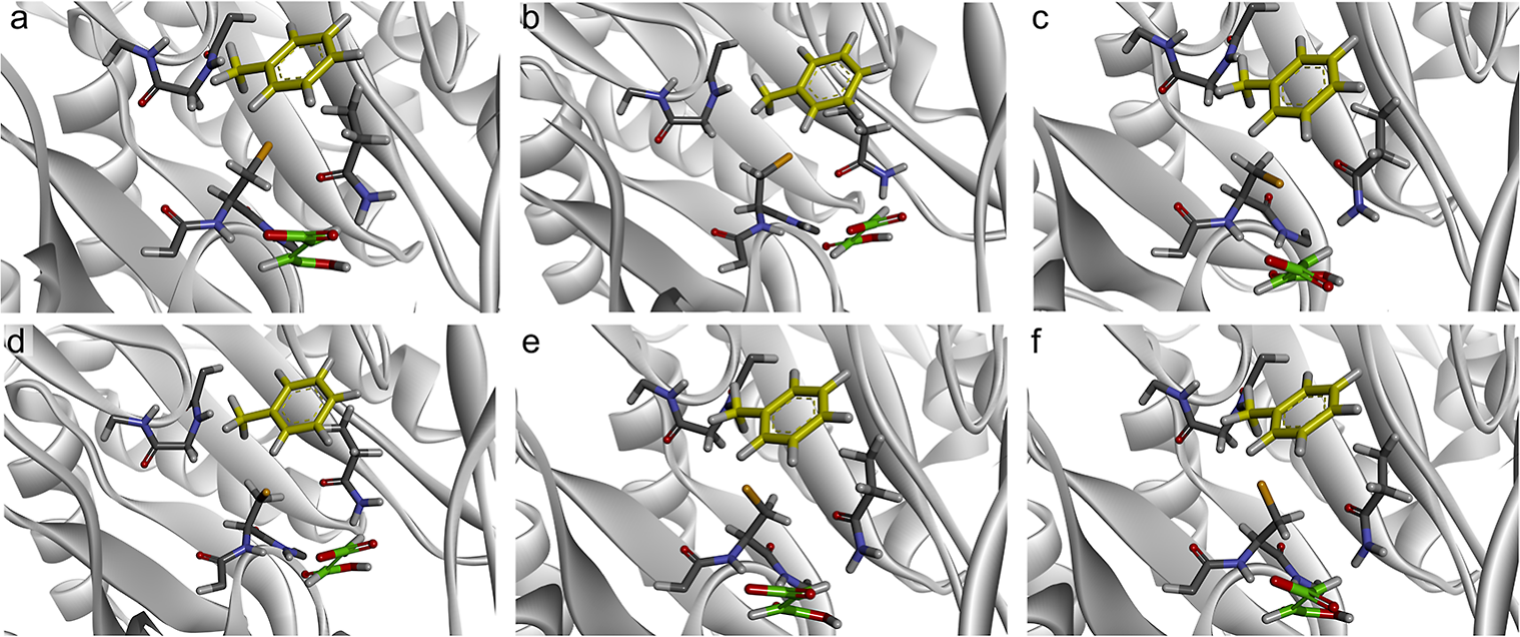
Conformational analysis of radical Cys493 with values of the dihedral angle C–Cα-Cβ-Sγ equal to (a) 52.5° for I1a, (b) 339° for TSrot1, (c) 280° for I1b, (d) 222.5° for TSrot2, (e) 186° for I1c, and (f) 144° for TSrot3.

### Step 2—Activation of Toluene

The activation of toluene proceeds by the HAT from its methyl group to the thiyl radical (Figure 3a). As was shown above, the radical Cys can exist in three different conformations corresponding to the C–Cα-Cβ-S dihedral angle values of 59° ± 29°, 169° ± 29° and 287° ± 21° in the QM/MM optimized models (I1a, I1c and I1b, respectively Figure 5). This diversity results in different geometries and energies of the transition states reached from the respective conformations (TS2a-c). To elucidate this issue, we have studied the activation of toluene for all three starting conformations of radical Cys (denoted a, b, and c in the following section). As a result, the value of the C–Cα-Cβ-S dihedral angle of the resulting TS changed somewhat, but stayed close to those of the starting states, yielding values of 83° for TS2a, 295° for TS2b, and 179° for TS2c (Figure 6).

**Figure 6:**
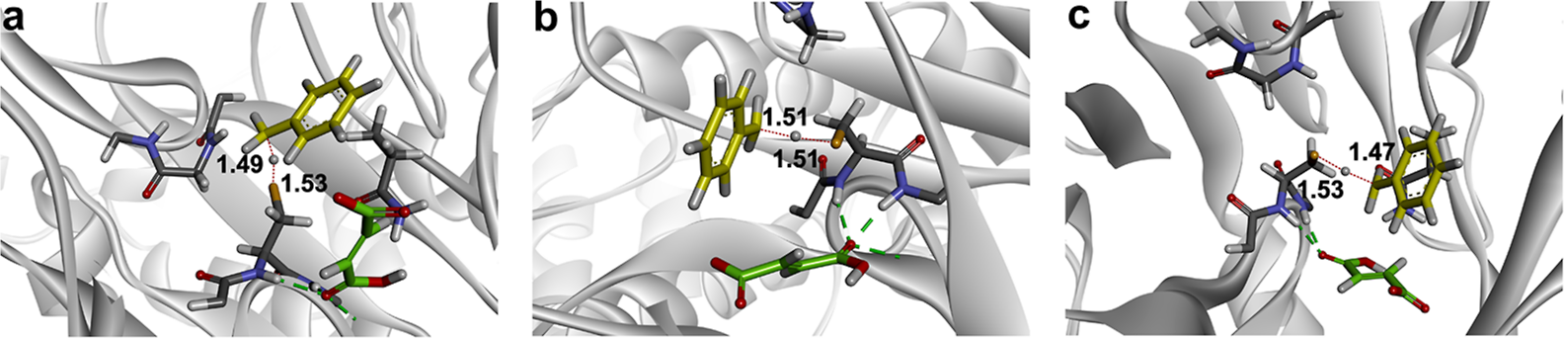
Three variants of toluene activation by radical Cys: (a) TS2a with the C–Cα-Cβ-S dihedral of 83°, (b) TS2b with the C–Cα-Cβ-S dihedral of 295°, (c) TS2c with the C–Cα-Cβ-S dihedral of 179°. Fumarate in green, toluene in yellow, and distances are in Å. The red dashed line marks formed/broken bonds in TS.

As was already apparent in the previous MD simulation, the respective conformations of the radical Cys differ in their relative abundance, with I1a being the most abundant. It became clear during the calculations that the active site structure of the model used had to be redefined because the initial position of Tyr197 was blocking the toluene positioning required for H-abstraction in pathway c. Although we started with an initial frame of the E:S complex containing Tyr197 bound to the fumarate (Figure S6c), the steric clash with toluene and subsequent repositioning of Tyr197 observed in the TS2c stage prompted us to re-evaluate all values with the alternative conformation of Tyr197. Inspection of the respective geometries showed that TS2c significantly changes the binding mode of fumarate, while toluene pushes Tyr197 out of the active site into a different conformation. Prompted by this observation, we analyzed MD simulations, which indicated that Tyr197 probably acts as a gating residue that can also attain three different conformations: the major population displays the C–Cα-Cβ-Cγ dihedral angle values of 77° ± 28°, and two smaller populations represented by broad peaks of dihedral angles with values of 302° ± 39° and 340° ± 20° (Figure S9A). Only in the first population (dihedral angle 77°), Tyr197 is directed toward fumarate. However, it does not necessarily form a H-bond with its carboxyl group, as shown in Figure S6c. Indeed, the OH group of Tyr197 comes into the H-bonding distance (below 4 Å) of the fumarate O1 oxygen atom only for approximately 6.5% of the simulation time (Figure S9B). The repositioned Tyr197 in TS2c results in a conformer with the C–Cα-Cβ-Cγ dihedral angle equal to 143°. Under these conditions, the energy of the whole system is elevated by approximately 18.2 kJ/mol, but we consider this step necessary because the reaction cannot proceed further with Tyr197 bound to fumarate. Therefore, the energy-minimized geometries of the respective I1a,b,c states with the alternative conformation of Tyr197 were used for the QM/MM calculations, creating comparable data sets for all three Cys conformations. The energy discontinuity of the reaction profile associated with the conformational rearrangement associated with the shift of Tyr197 was corrected by matching, with a factor of −18.2 kJ/mol (i.e., the energy difference between I1a obtained before and after the Tyr197 shift). Therefore, all energies reported for these and later steps in the text are corrected for this factor, while the originally calculated energy values are reported in Supporting Information (see Table S3). The energy elevation may be an artifact of the model, as not all residues surrounding Tyr197 in the new positions were flexible.

The shift of Tyr197 from the active site changes the relative energy of I1a with respect to I1b and I1c, restoring a picture more similar to that observed in MD. The I1a turned out to have the lowest energy of −28.2 kJ/mol, surprisingly followed by I1c at −13.7 kJ/mol and finally I1b at 4.9 kJ/mol. This suggests a reversal of the energetic preferences of I1b and I1c, compared to their calculated energies after step 1 of the reaction.

Each of the three conformations of radical Cys theoretically allows for the activation of toluene by the thiyl radical, but via totally different approaches (Figures 6 and S10, Table S9). The TS2a transition state is characterized by S–H and C–H distances of 1.53 Å and 1.49 Å, respectively, and the three atoms are aligned almost in line (angle S–H–C of 166°). As the radical Cys is oriented away from the active site and toward Gly829, the toluene has to approach very closely to the surface of the active site cavity, and the only possible orientation enforces positioning the toluene molecule above the fumarate cofactor and the thiyl radical. Because the activated hydrogen is localized between toluene and fumarate, the subsequent C–C bond formation would proceed without inversion of the methyl group configuration. In the TS2a, the spin density is already mostly localized on the activated substrate (0.54 on the methyl carbon and 0.23 on the aromatic ring with 0.35 still localized on the Sγ atom of Cys. The energy of TS2a is 49.7 kJ/mol, which results in a barrier for toluene activation of 77.9 kJ/mol.

TS2b allows for a seemingly more convenient positioning of toluene in the active site, as the radical Cys faces the active site, close to Gln707. In this conformation, both distances in TS2 for d­(S–H) and d­(C–H) equal 1.51 Å, while the S–H–C angle is slightly more obtuse, reaching 178°. The spin density on the substrate reaches 0.56 at the methyl carbon atom, 0.16 at the aromatic ring and 0.29 at the Sγ atom of Cys. The activation barrier turned out to be of the same magnitude as for TS2a (74.4 kJ/mol), which, together with an elevated energy of I1b, results in the highest absolute energy of TS2b (79.3 kJ/mol).

Finally, the distances in TS2c for d­(S–H) and d­(C–H) are 1.47 Å and 1.53 Å, respectively, while the S–H–C angle is 167°. The spin density distribution is similar to those reported above for TS2a and TS2b, with 0.5 at the methyl carbon, 0.14 spread over the aromatic ring, and 0.26 at the Sγ atom of Cys (see Figure S15B). In this conformation, the radical Cys is localized directly over the fumarate cosubstrate. Therefore, toluene has to slide in between Cys493 and fumarate to be activated, but its approach is blocked by Leu492. As a result, the plane of a newly formed benzyl radical is positioned at an angle of 109.65° with respect to the fumarate plane. Remarkably, only this conformation leads to H abstraction from the face of toluene opposite to that oriented toward fumarate, resulting in inversion of configuration after C–C bond formation. While the energy of I1c is slightly higher than that of I1a (by 15 kJ/mol), the barrier associated with toluene activation in TS2c turns out to be the lowest of all three conformers (47.6 kJ/mol), and the resulting energy difference of TS2c and I1c is only 34.4 kJ/mol. Therefore, the TS2c-type activation is kinetically the most favorable of all three variants, as well as consistent with the experimental observations that the reaction involves inversion of configuration at the methyl group.^58^

In all cases, the I2 intermediates exhibit higher energy than the I1 intermediates by 20–26 kJ/mol (see Figure S10, Table S4 and Figure 3b), and the relative energies exhibit the same order as in the case of I1, with I2a at the lowest (−7.7 kJ/mol), I2c at an intermediate (13.2 kJ/mol) and I2b at the highest level (27.7 kJ/mol). As the TS2c barrier was clearly the lowest and the reaction path is favored by the experimentally established inversion of the methyl group, the following steps of the reaction were continued from I2c.

### Step 3—C–C Bond Formation

In the I2 intermediate, the benzyl radical derived from toluene is localized directly over the fumarate cosubstrate. The C–C bond can, in principle, be formed by the attack of the benzyl radical at any one of the carbon atoms of the double bond of fumarate, which are labeled as “distal” (C2) or “proximal” (C3) to Cys493 in the following, based on their distances (further away or closer, respectively). To determine the regioselectivity of the process, we have studied both pathways, designating attack on the distal C2 atom as variant a, and on the proximal C3 atom as variant b. First, we used an MD simulation of the E:S complex to assess the probability of attack by the benzyl radical on either of the fumarate carbon atoms. Although this complex contains the Gly radical and toluene instead of the benzyl radical, these simulations provided a good approximation of the behavior of the benzyl radical intermediate, especially since Cys493 is in the protonated nonradical state in both cases (which models the behavior of the Cys residue correctly), whereas the state of Gly829 most likely does not have much influence on the active site.

The distances from the methyl C atom of toluene to each of the C atoms of the fumarate double bond were analyzed in the MD trajectory, with the histograms of distance distributions presented in Figure S11. We observed that toluene approaches closer to the distal than the proximal C atom (6.0 Å and 2.9 Å or 6.85 Å and 3.2 Å for the median and minimal distances between the methyl carbon of toluene and the distal C2 or proximal C3, respectively). Therefore, we estimate that there is a higher probability of the benzyl radical attacking fumarate at the distal C atom (pathway a) compared to an attack at the proximal atom (pathway b).

Similarly to the analysis of the previous step, we observed another conformational shift when assessing the QM/MM models of this step, which became apparent during the optimization of TS3a. This time, the change applies to a conformational adjustment of Trp613, which also triggers changes in the conformations of Asn611, Ser495, and Asn615 (Figure S10). As Trp613 and Asn615 directly interact with the unreactive face of fumarate, these modifications again resulted in a change of the MM energy of the whole system, amounting to a value of 212 kJ/mol. The discontinuity of the energy profile was corrected by aligning the energies of the I2 state obtained from a scan of the internal reaction coordinate (IRC) from TS3a and TS3b with the previously determined energy of the I2c state (13.2 kJ/mol), obtained from TS2c.

In TS3a, the benzyl radical approaches the distal C atom of the double bond of fumarate, forming a new C–C bond. The C–C distance in TS3a is 2.23 Å (Figure 7a, Table S9). Due to the steric interaction of the phenyl ring of the radical with some active site residues (Gln707 and Ile617) and some distortion of the fumarate structure, the bond being formed is not directly perpendicular to the fumarate frame, but at a slightly wider angle of 106°, which is in good agreement with the resulting sp^3^ hybridization of the intermediate. The fumarate is also significantly distorted, with the distal atom moving out of the plane and closer to the benzyl radical, which corresponds to the change from sp^2^ to sp^3^ hybridization of the C atom (see Table S9). The spin density is distributed on the benzylic carbon atom (0.58) and the aromatic ring (0.18), as well as the proximal carbon atom of the former double bond of fumarate (0.32). In contrast, the distal carbon atom exhibits a negative spin population of −0.12 (Figure S15C).

**Figure 7:**
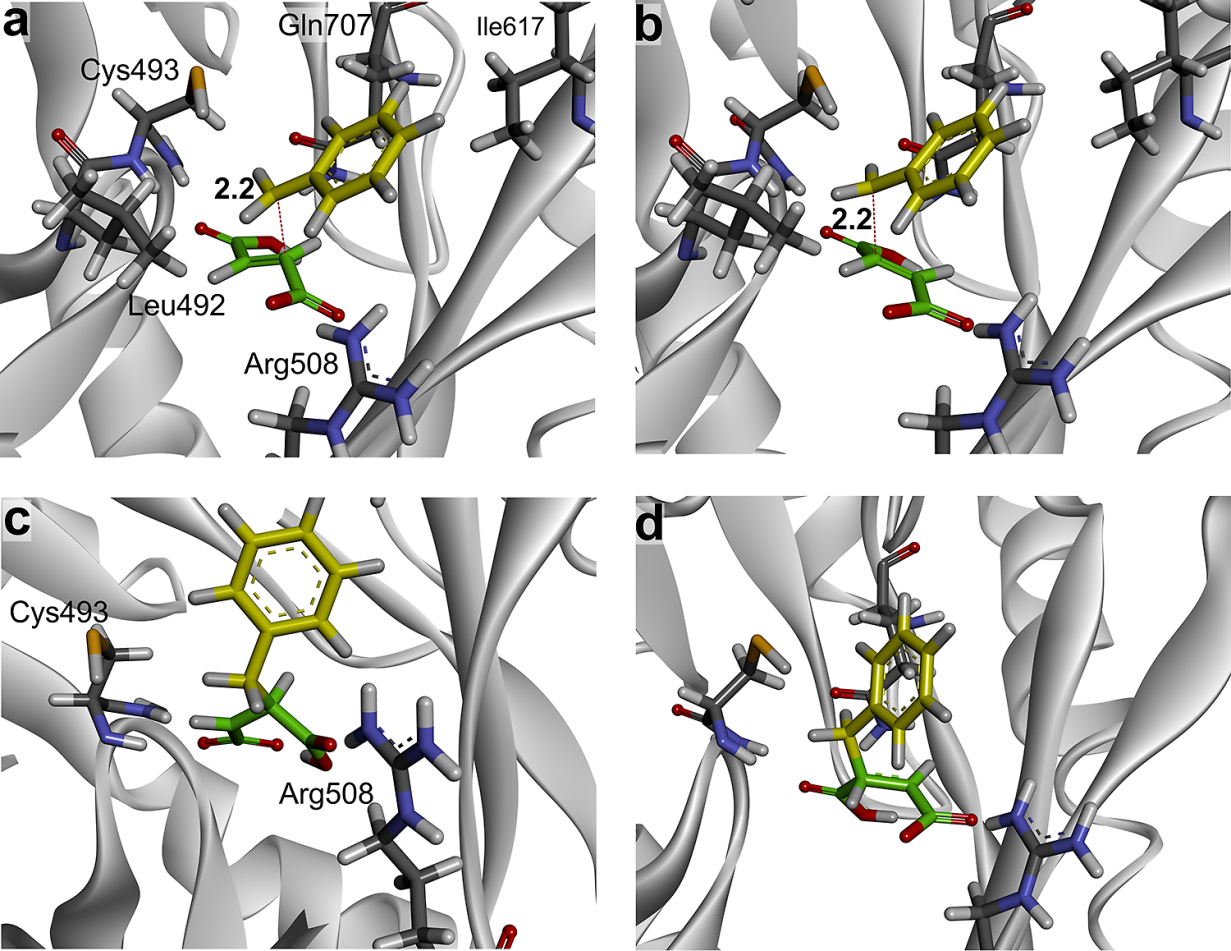
Formation of the C–C bond: (a) TS3a attack of radical toluene on the distal C atom of the fumarate double bond, (b) TS3b attack of radical toluene on the proximal C atom of the fumarate double bond; radical benzylsuccinate intermediates (c) I3a and (d) I3b.

The completion of this reaction step leads to the formation of an (*R*)-benzylsuccinyl radical intermediate, I3a, with a newly formed C–C bond of 1.55 Å and a radical at the proximal C carbon (Figure 7c). The change of hybridization of the distal C atom from sp^2^ to sp^3^ bends the acidic part of the benzylsuccinyl molecule, bringing the two carboxylic groups together. As a result, an internal H-bond is formed between the carboxylic groups, and the proton shifts spontaneously between the formerly protonated and deprotonated carboxyl groups (Figure 7c). This effect also significantly weakens the electrostatic interaction with Arg508 and may later help in the release of the product.

In the alternative TS3b, the new C–C bond is formed with the benzyl radical approaching the proximal C atom of the double bond of fumarate. In this case, the C–C bond distance is 2.2 Å and the C^dis^–C^prox^–C^meth^ angle is 108° with the bend toward Cys493. Similarly, as in the case of TS3a, the plane of the benzyl radical is not parallel to that of the fumarate, but slightly bent to one side (Figure 7b). Although the proximal C atom is also drawn out of the plane of the fumarate toward the benzyl radical, the distortion is significantly smaller than in TS3a, indicating higher sp^2^ character in TS3b. The spin density is evenly distributed between the benzylic carbon (0.56) and the distal carbon atom of the double bond of fumarate (0.54), while the proximal carbon atom exhibits a negative spin population of −0.16. The discrepancies in spin density distribution indicate a significant difference between TS3a and TS3b.

The formation of the C–C bond leads to the production of an (*R*)-benzylsuccinyl radical intermediate I3b, shown in Figure 7d. Unlike in the case of I3a, the carboxyl groups remain far apart, and no internal H-bond forms between them. As a result, the distal carboxyl group still forms a strong salt bridge with the side chain of Arg508, which will profoundly influence the next step of the reaction.

The analysis of the energy profile (Figure 3b) indicates that the formation of the C–C bond proceeds preferentially at the distal C atom, as the energy barrier for TS3a is 64.2 kJ/mol while that for TS3b is 110.4 kJ/mol. This high difference in energy barriers is associated with the steric constraints imposed on the formation of the C–C bond at the proximal C atom, which is in close vicinity to Cys493 and Leu492. Also, the absolute energy of I3a is significantly lower than that of I3b (−15.4 vs 39.5 kJ/mol).

### Step 4—Quenching of the Benzylsuccinyl Radical Intermediate

The final step of the reaction is associated with quenching of the radical intermediate. This step involves HAT from Cys493 to the radical carbon atom. During the optimization of the stationary points defining this step, another conformational shift occurred, resulting in a decrease of total energy by 73 kJ mol^–1^. This conformational shift was associated with a flip of Arg826 combined with the relaxation of the loop containing Gly829, as well as a small accommodation of the SH group of Cys493. Besides these details, the models are essentially identical (RMSD 0.076 Å), and the residues that changed their conformations are not in any contact with the reactants.

As a consequence of the two potential pathways considered in step 3, the conversion of the benzylsuccinyl radicals to the product benzylsuccinate can also proceed in two different ways. Pathway a involves the HAT to the proximal C2 atom of the benzylsuccinyl radical intermediate (TS4a), while pathway b requires HAT to the distal C3 atom (TS4b)—see Figure S12.

Furthermore, quenching of the radical intermediate has been shown experimentally to proceed usually in a *syn* geometry (i.e., at the same face of the double bond as the C–C addition).^59^ However, we recently reported results of an H/D exchange experiment^46^ indicating that *R*-benzylsuccinate is slowly deuterated upon incubation in D_2_O in the presence of active BSS, producing *d*_1_-benzylsuccinate as the major product, with traces of *d*_2_-benzylsuccinate produced at a significantly lower rate.^46^ These observations indicated that, on one side, the last reaction steps are reversible, while on the other, the HAT reactions involved in quenching of the benzylsuccinyl radical are not absolutely enantioselective and H/D atoms may occasionally be transferred to or removed from the other (anti) face of the intermediates. Therefore, we decided to investigate a potential anti-addition variant of the pathway a (using a β superscript to denote anti-addition, in contrast to α for *syn*-addition) (Figure S12).

After the C–C bond is formed at the distal C atom in pathway a, the radical is localized at the proximal carbon atom, close to Cys493. This allows for a relatively easy HAT from the SH group, which at TS4^α^a exhibits almost linear geometry (S–H–C angle of 171°) and 1.52 Å distance between the H and both S and C atoms (Figure 8a, Table S9). Because this transition state is reached with minimal rearrangement of the intermediate, its barrier turns out to be the lowest (45.1 kJ/mol—see Figure 3b). The spin density (Figure S15d) at the TS is divided mainly between the S and C atoms (0.37 and 0.65).

**Figure 8:**
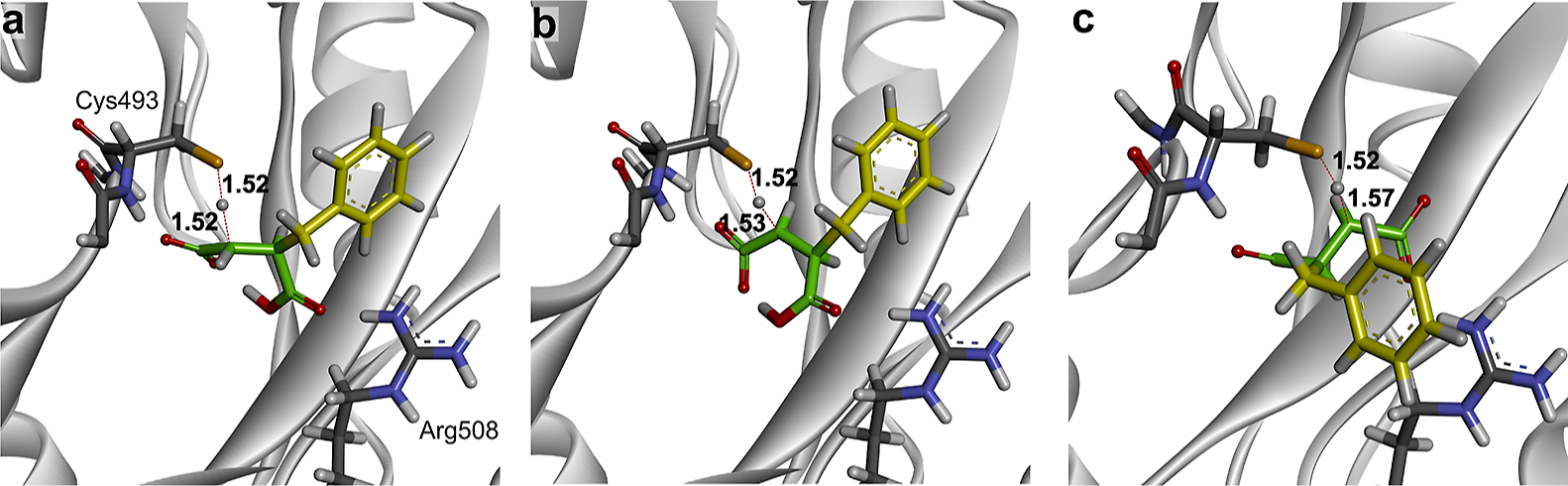
TS4 for quenching the radical benzylsuccinate intermediate by HAT from Cys493: (a) TS4^α^a a *syn*-addition to the proximal C2 atom, (b) TS4^β^a an anti-addition to the proximal C2 atom, (c) TS4b *syn*-addition to the distal C3 atom.

In the TS4^β^a (Figure 8b), the H atom from the SH group of Cys489 would be transferred in the *anti*-geometry to the benzylsuccinyl radical, which requires partial rotation of the now deprotonated C1 carboxyl group, removing it from the H-bonding pocket. As a result, despite the similar geometries of TS4^β^a and TS4^α^a (C–H and S–H distances of 1.53 and 1.52 Å, 170° S–H–C angle), the energy barrier connected with TS4^β^a is relatively high (90.9 kJ/mol—see Figure 3b).

Quenching of the benzylsuccinyl radical formed by addition at the proximal C atom in pathway b turns out to be more difficult. This is because the radical is located far from Cys493 on the distal C atom, and to reach TS4b the intermediate has to rotate in the active site (Figure 8c). This also implies that the deprotonated carboxyl group has to move away from the positively charged guanidyl group of Arg508. The geometry of TS4b resembles that of TS4a with an almost linear arrangement of S–H–C (170°) and slightly longer C–H distance relative to S–H distance (1.57 vs 1.52 Å). At the same time, the transfer of the spin density is less advanced than in the case of TS4a (0.29 and 0.72 at S and C atoms, respectively). The energy of the TS4b is very high (93.4 kJ/mol), but the energy difference between I3b and TS4b is in fact smaller (53.9 kJ/mol) than that between I3a and TS4a (60.5 kJ/mol). This is mainly due to the highly elevated energy of I3b.

The completion of HAT in both cases results in the formation of *R*-benzylsuccinate, and the obtained E:Pa state exhibits significantly lower energy (−29 kJ/mol) compared to that of E:Pb (−2.9 kJ/mol). Even though in both states, E:Pa and E:Pb, the enzyme is in complex with *R*-benzylsuccinate, E:Pb has higher energy due to steric clashes of the benzyl ring positioned closer to the cysteine. At this stage, the enzyme is ready for product release, and we postulate that this is precisely what happens.

### Step 5—HAT between Gly829 and Cys493 for the E:P Complex

For over two decades, it has been postulated that the last step of the BSS mechanism is the final transfer of the radical from Cys493 to Gly829 (Figure 4). This is due to the fact that the thiyl radical is not observed in EPR. Recently, we have analyzed this step in detail^46^ and demonstrated that the process preferentially proceeds via a *re*-side attack. To take over the H atom from Gly829, Cys493 has to change its conformation again, i.e., rotate counterclockwise from the position attained during quenching the benzylsuccinate radical (Cys93 C–Cα-Cβ-Cγ dihedral angle of 182°) to the conformation where its S atom is closest to Gly829 (dihedral angle 52°). The MD simulations conducted for the E:P complex containing a radical Cys493 and a monoprotonated benzylsuccinate with its protonated group facing Arg508 indicate some difference in the population of the three main conformers of Cys493, compared to that of the E-Cys^rad^:S complex (Figures S13 and S7). Although the population with the C–Cα-Cβ-Cγ dihedral angle around a local maximum of 54° ± 23° still turns out to be the most probable (65% of simulation time), the conformation facing the interior of the active site, i.e. at dihedral of 172° ± 22°, is more prevalent (20% vs 2% for E:S complex), followed by the transitory population with the dihedral angle of 287° ± 28° (14%). On the other hand, when the product is removed, the conformation with Cys493 facing Gly829 becomes predominant (95% of snapshots—Figure S14A). The same result is obtained from two independent simulations conducted for the apo enzyme (PDB 4PKF) derived from the earlier study.^46^ QM/MM modeling revealed that the conformation change of Cys493 from that in E:Pa to the one allowing HAT to Gly829 (E:Pa rot, i.e. with a dihedral angle of 54°) is associated with an increase of energy by 6.1 kJ/mol, which is of the same magnitude as the difference between I1c and I1a before the shift of Tyr197 (Figure 3a).

The calculated C–H and S–H distances of TS5 are 1.57 and 1.48 Å, respectively, and the S–H–C angle is 166.5° (Figure 9, Table S9, and Figure S15e). Analysis of the energies indicates that the HAT between Gly829 and Cys493 in the E:P complex is highly endergonic, requiring more than 40 kJ/mol (Figure 3b). This step is energetically symmetrical to step 1, i.e. the barrier for transferring the H atom from Gly to the thiyl radical is higher than the barrier of its transfer from Cys493 to the glycyl radical (99 kJ/mol vs 57 kJ/mol). Therefore, we think that HAT for BSS in complex with the product is highly improbable.

**Figure 9:**
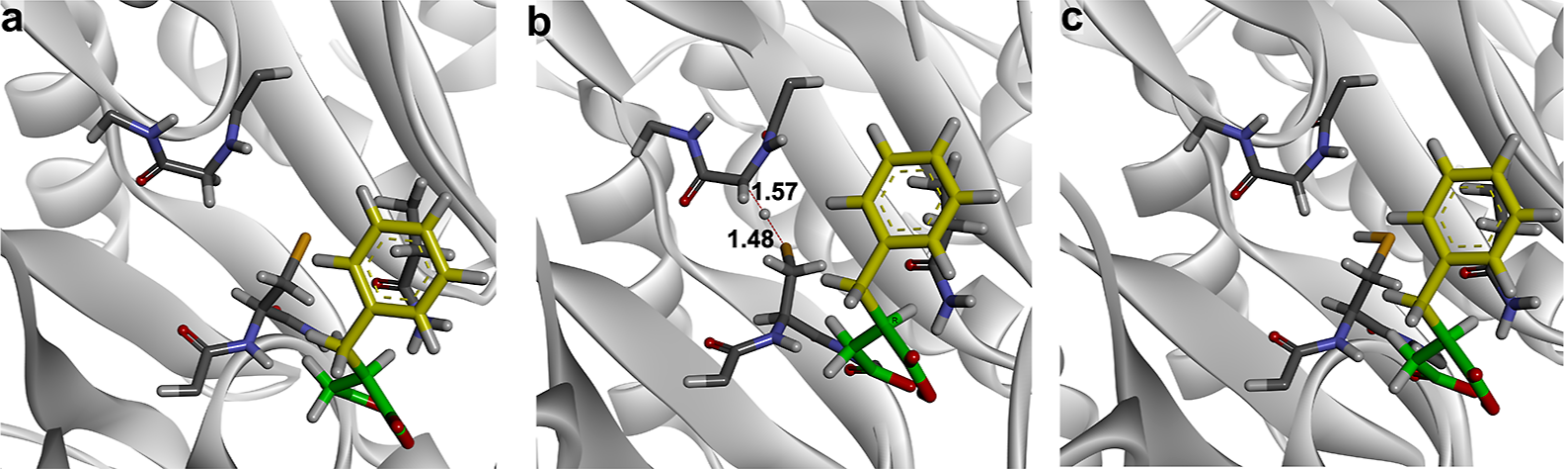
HAT between Gly829 and Cys493 for the E:P complex: (a) E:P-rot radical Cys rotated to the most stable conformation, (b) TS5 pro*R* H atom transfer from Gly to Cys, (c) radical E:P-Gly.

In our earlier study on the HAT reaction between the glycyl and thiyl radical states of BSS, we obtained very similar results with either substrate- or product-bound BSS, while apo-BSS with an empty active site showed the reverse behavior, i.e., lower energy of the glycyl-than the thiyl-radical state.^46^ Thus, our calculations indicate that if HAT between Gly829 and the thiyl radical truly occurred in the E:P complex, it would be the rate-limiting step of the overall reaction (with a barrier of 75.2 kJ/mol). Since this would effectively mask any kinetic isotope effects associated with labeled substrates, which were observed in the experiment,^60^ we can safely assume that HAT does not take place in the E:P complex, and the product is released immediately after formation. Therefore, the radical appears likely to remain localized at Cys493 after completion of the C–C addition reaction, instead of moving to Gly829 in the E:P complex. Because the glycyl/thiyl HAT still needs to be resolved to close the catalytic cycle, we speculate that it may either occur (in an energetically favorable reaction^46^) in the apoenzyme after benzylsuccinate release, or that BSS may employ a concerted reaction for the release of benzylsuccinate and glycyl/thiyl HAT.

### Reaction Enantioselectivity

The reaction catalyzed by BSS is highly enantioselective, yielding exclusively (*R*)-benzylsuccinate as the product.^61^ We additionally confirmed the high reaction enantioselectivity of recombinant BSS expressed in *Aromatoleum evansii* with a chiral separation of the reaction mixture. The chromatogram did not show a detectable peak of *S*-benzylsuccinate, and only a peak for *R*-benzylsuccinate was detected (Figure S20). Notably, we detected an additional peak with a retention time close to that of *S*-benzylsuccinate. However, its UV–vis spectrum differed from that of benzylsuccinate (Figure S20C), and it was also present at the start of the experiment (data not shown), which indicates that it corresponds to the impurity derived from the cell extract.

In a previous study, we used cluster models to rationalize this behavior, which indicated that (*S*)-benzylsuccinate can be formed only when the fumarate is bound in a conformation that presents its other face (pro*S*) to be attacked by the benzyl radical. While binding of fumarate in this conformation can easily be envisaged, the pro*S* binding mode results in subtle changes in the positions of the proximal and distal carbon atoms capable of reacting with the benzyl radical.

To assess whether fumarate binding in either of the two poses provides any thermodynamic preference for the reaction, we have conducted MD simulations for the pro*R* and pro*S*-BSS/fumarate complexes. However, no significant differences were obtained between these two configurations, and any deviations were within the error of the method, especially if the variability introduced by repeated simulations is taken into account. The average Δ*G* values of binding according to the ‘Generalized Born’ method were in the range of −191 to −143 kJ mol^–1^ (SD 6 −17 kJ mol^–1^) for pro*R* fumarate and −192 to −152 kJ mol^–1^ (SD 6.5–11 kJ mol^–1^) for pro*S* fumarate. A similar effect was observed for the Δ*G* values of fumarate binding computed using the Poisson–Boltzmann method (see Supporting Information for more details, Figure S16).

As the Δ*G* of fumarate binding does not appear to be responsible for the observed pro*R* enantioselectivity of the reaction, we decided to conduct a similar analysis of the MD trajectories for the model with fumarate in the pro*S*-conformation as in the case of the pro*R* pathway. We analyzed the dynamic behavior of toluene and its approach toward the proximal or distal C atoms of the fumarate double bond. Analysis of the distances from three independent MD simulations indicated that toluene indeed can approach the fumarate bound in the pro*S* orientation more easily at the proximal C3 atom than at the distal C2 atom (minimal and median distances 3.02 Å and 7.1 Å or 3.13 Å and 7.4 Å for the proximal and distal C atoms, respectively—Figure S17). This pattern of preferred attacks is directly opposed to that observed with fumarate bound in the pro*R* orientation. The simulations with fumarate in the pro*S* orientation also show larger average distances between fumarate and toluene, compared to those with fumarate in the pro*R* orientation (medians 6.0 and 6.85 Å for the distal and proximal C atom, respectively, Figure S11).

Furthermore, we analyzed the pro*S* reaction pathway also by QM/MM modeling, starting from the benzyl radical intermediate of the standard pathway (I2c) assuming that the binding mode of fumarate in either pro*R* or pro*S* conformation does not significantly influence H abstraction from toluene (Figure 10). A detailed description of the transition states is provided in the supplement (Figures S18 and S19, Tables S2, S5, S7, S10). Unexpectedly, we observed the same regioselectivity pattern as in the case of the pro*R* pathway, i.e., a kinetic preference of the benzyl radical to attack on the distal carbon atom (C2), compared to the proximal C3 atom of the double bond of fumarate (44.7 vs 92.9 kJ/mol for TS3a vs TS3b, Figure 11).

**Figure 10:**
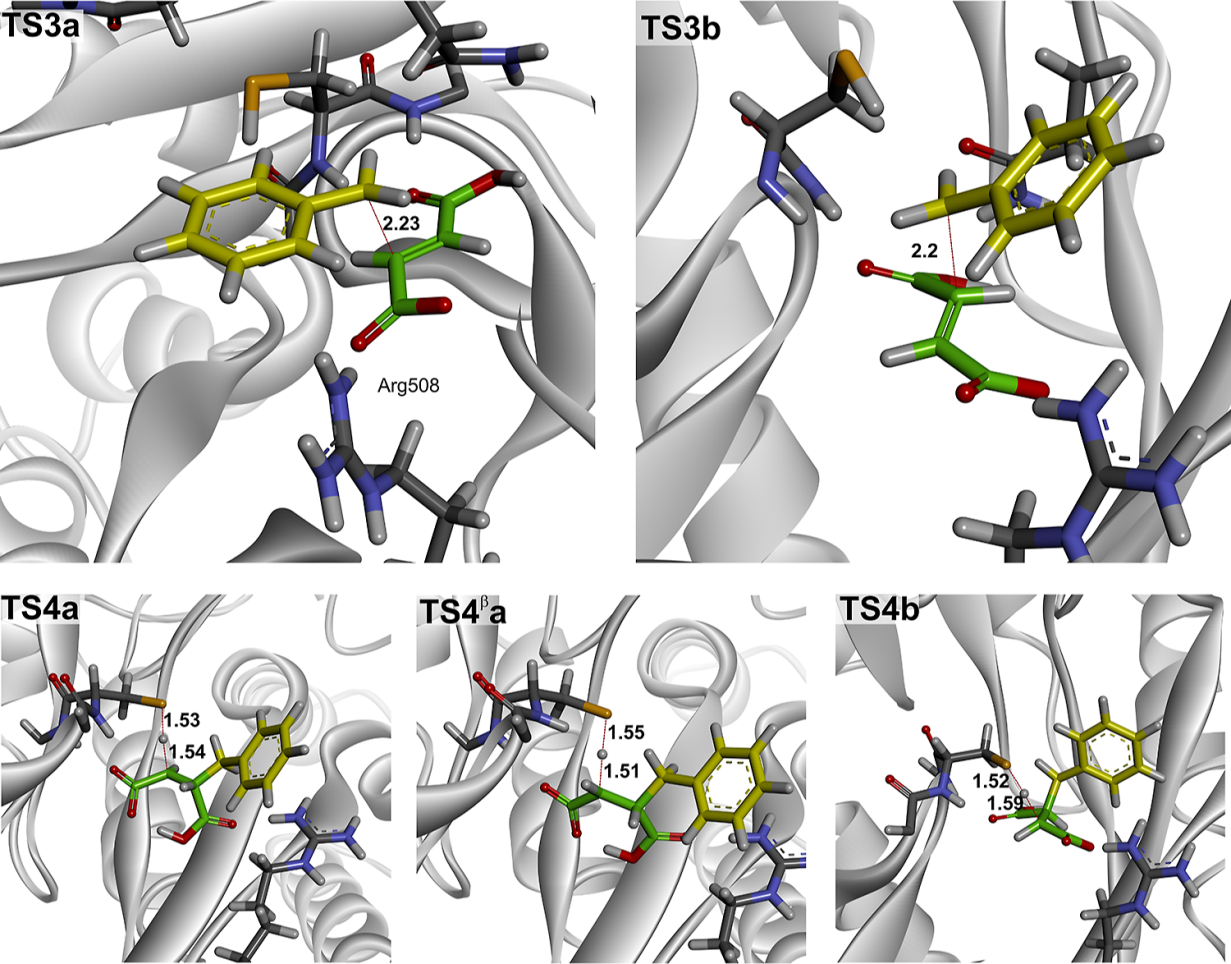
Geometries of transition states calculated for the pro*S* pathway.

**Figure 11:**
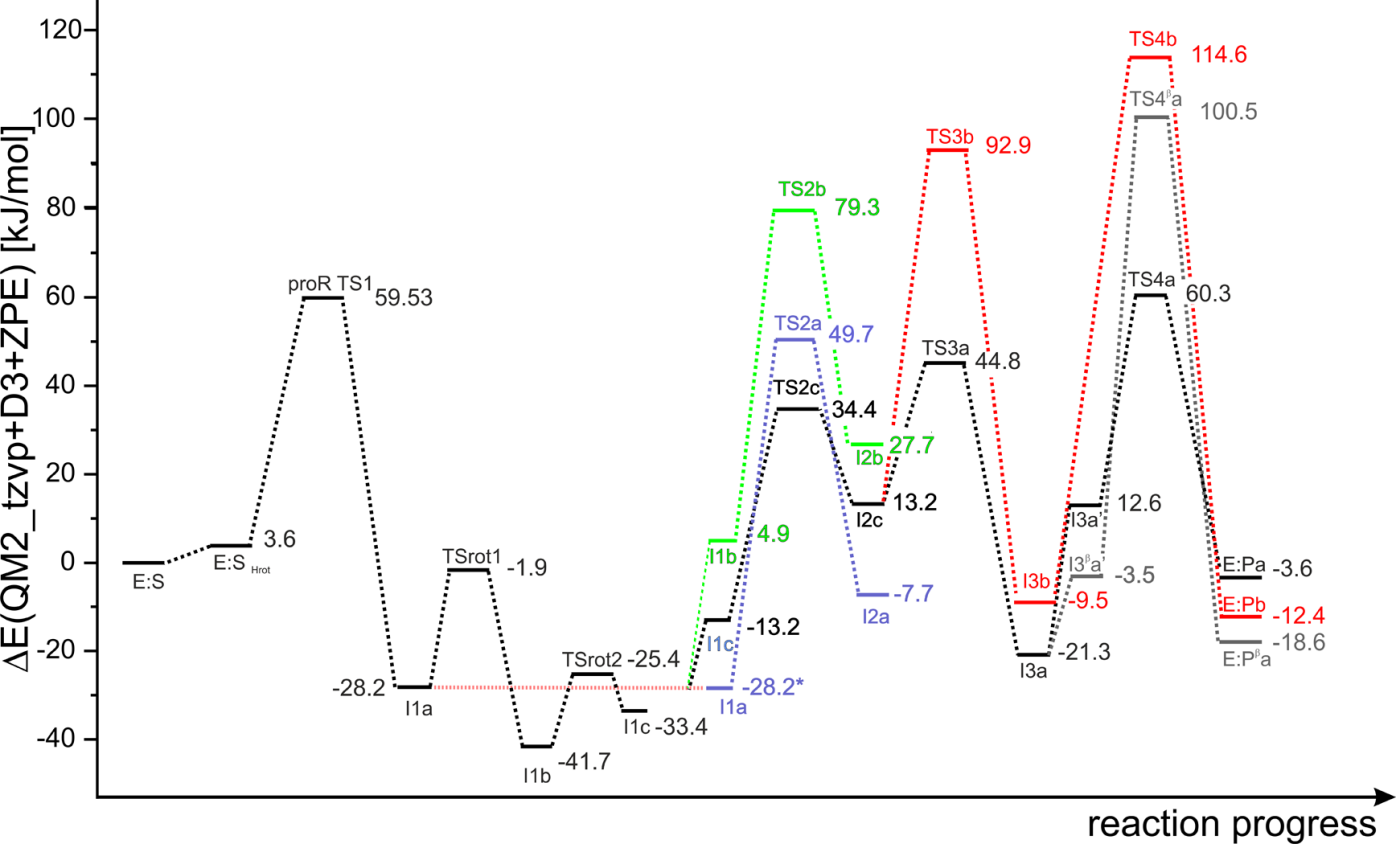
Energy diagram for the pro*S* conformation of the fumarate. The pro*S* profile was matched at I2c.

The formation of the *S*-benzylsuccinyl radical is followed by a radical quenching process, which is associated with barriers of 60.3 kJ/mol (TS4a) and 114.6 kJ/mol (TS4b) for the quenching of proximal and distal radicals. Noticeably, the energy barrier for the C–C addition even along the kinetically preferred pathway a (at a distal C2 atom) is significantly higher (81.6 kJ/mol) than that of the pro*R* pathway (60.5 kJ/mol), thus favoring the production of *R*-over *S*-benzylsuccinate. The preferential mode of C–C bond formation is followed by radical quenching at a lower energy barrier (TS4a 60.3 kJ/mol), which would suggest the formation of *S*-benzylsuccinate.

Interestingly, we also detected a different protonation mode of the *S*-benzylsuccinyl radical in the pro*S* pathway. Compared with the pro*R* pathway, there is no internal H-bonding detected between the carboxyl groups of the product radical (I3a′ and I3^β^a’—Figure S19 and Table S5). This may contribute to the enzyme’s specificity for *R*-benzylsuccinate production by elevating the energy barrier of the radical-quenching step in the pro*S* pathway.

Finally, similarly to the pro*R* pathway, the HAT in the anti conformation was associated with a significantly higher barrier (TS4^β^a, 100.5 kJ/mol), indicating that the *d*_2_-benzylsuccinate formation observed in the experiment is not a result of unspecific exchange in the *S*-product.

### Microkinetic Analysis of Reaction Enantioselectivity

We decided to use microkinetic analysis to explain the experimentally observed enantioselectivity of the BSS reaction, which yields R-benzylsucciante exclusively. We used Gibbs free energies obtained from modeling of the pro*R* and pro*S* reaction pathways to calculate kinetic constants for the elementary steps of both pathways.

Then, we assumed that quenching of the product radical is a rate-limiting step. This is because quenching of the benzylsuccinyl radical is associated with the lowest kinetic constant value of all partial steps of the mechanism (see Tables 1 and S6, S8) when calculated with respect to the preceding stationary point. With such assumptions, the rates of the overall reaction should only depend on the concentration of the BSS enzyme containing the cysteine with SH group and benzylsuccinyl radical [BSS-C:BS·].

1 $$V=k_{5}\left[BSS-C:{BS}^{\cdot }\right]$$

**Table 1:** Kinetic Constants (*k*_*i*_) and Kinetic Constants Corrected for Tunnelling (*k* ^*T*^_*i*_) Calculated from Gibbs Free Energy Barriers in QM/MM for the pro*R* Pathway, as Well as for Two Steps of the pro*S* Pathway

	Δ*G*(QM2^303 K^) [kJ/mol]	*k*_i_ [s^–1^]	*k* ^*T*^_i_ [s^–1^]
*k*_2_	65.8	29	55.3
*k*_–2_	93.4	4.99 × 10^–4^	9.5 × 10^–4^
*k*_3_	29.1	6.15 × 10^7^	6.17 × 10^7^
*k*_–3_	39.9	8.45 × 10^5^	8.48 × 10^5^
*k*_4_	13.7	2.7 × 10^10^	2.7 × 10^10^
*k*_–4_	10.9	8.37 × 10^10^	8.41 × 10^10^
*k*_5_	50.0	1.50 × 10^4^	3.1 × 10^4^
*k*_–5_	27.9	9.63 × 10^7^	2.0 × 10^8^
*k*_6_	55.6	1.62 × 10^3^	1.9 × 10^3^
*k*_–6_	68.6	9.25	10.87
*k*_7_	68.9	8.45	21.4
*k*_–7_	78.1	0.22	0.55
*k*_9_	2.3^a^	2.5 × 10^12^	n.d
*k*_–9_	78.3^a^	0.2	n.d
*k*_9_^’^	103.3	1.0 × 10^–5^	1.8 × 10^–5^
*k*_–9_^′^	63.5	71.6	130
^S^ *k*_4_	39.0	1.21 × 10^6^	1.4 × 10^6^
^S^ *k*_–4_	65.6	30.6	35.5
^S^ *k*_5_	91.4	1.11 × 10^–3^	2.5 × 10^–3^
^S^ *k*_–5_	70.9	3.72	8.5

a Data calculated for apo-BSS in^46^.

Furthermore, we assumed that the binding of substrates is not limiting the observed reaction rate (substrate saturation conditions). The reverse reaction (i.e., binding of benzylsuccinate and its conversion to the radical) can be neglected at the initial stage of the reaction, due to the low concentration of the available product, which results in a low probability of its binding back and reversing the reaction. With these assumptions, we have derived an equation describing the reaction velocity using the King-Altman algorithm^55^ (see Supporting Information for the full derivation).

2 $$\frac{V}{E_{T}}=\frac{k_{2}k_{3}k_{4}k_{5}k_{6}k_{7}}{\begin{array}{l} k_{2}k_{3}k_{4}k_{5}k_{6}+k_{2}k_{3}k_{4}k_{5}k_{7}+k_{2}k_{3}k_{4}k_{5}k_{-6}+k_{2}k_{3}k_{4}k_{6}k_{7}+k_{2}k_{3}k_{4}k_{7}k_{-5}+k_{2}k_{3}k_{4}k_{-5}k_{-6}+k_{2}k_{3}k_{5}k_{6}k_{7}\\ +k_{2}k_{3}k_{6}k_{7}k_{-4}+k_{2}k_{3}k_{7}k_{-4}k_{-5}+k_{2}k_{3}k_{-4}k_{-5}k_{-6}+k_{2}k_{4}k_{5}k_{6}k_{7}+k_{2}k_{5}k_{6}k_{7}k_{-3}+k_{2}k_{6}k_{7}k_{-3}k_{-4}\\ +k_{2}k_{73}k_{-3}k_{-4}k_{-5}+k_{2}k_{-3}k_{-4}k_{-5}k_{-6}+k_{3}k_{4}k_{5}k_{6}k_{7}+k_{4}k_{5}k_{6}k_{7}k_{-2}+k_{5}k_{6}k_{7}k_{-2}k_{-3}+k_{6}k_{7}k_{-2}k_{-3}k_{-4}\\ +k_{7}k_{-2}k_{-3}k_{-4}k_{-5}+k_{-2}k_{-3}k_{-4}k_{-5}k_{-6} \end{array}}$$

Finally, we calculated the rate constant using eq 2 to elucidate the enantioselectivity of the enzyme. The estimated *k*_cat_ for reaction with toluene at 303 K along the pro*R* pathway is 0.029 s^–1^ *E_T_. When for the calculation of the *k*_cat_^S^ *k*_4_,^S^ *k*_–4_, and ^S^ *k*_5_ are used (Table 1), the value of *k*_cat_ drops to 6.65 × 10^–4^ s^–1^*E_*T*_. This means that the reaction leading to *R*-benzylsuccinate is 43-fold faster than that yielding *S*-benzylsuccinate. In case Wigner’s nuclear tunnelling is taken into consideration, the estimated *k*_cat_ for reaction along the pro*R* pathway is 0.047 s^–1^, while for the pro*S* pathway it is 1.5 × 10^–3^ s^–1^ *E_*T*_, leading to 31-times faster production of *R*-benzylsuccinate than of *S*-benzylsuccinate. The estimation effect of the tunnelling also indicates that the overall rate may be accelerated by a factor of 1.6 for the pro*R* pathway and 2.3 for the pro*S* pathway, which indicates that tunnelling acts against the enantioselectivity of the enzyme, although it does not compromise it to any significant degree. The barrier-dependent kinetics is additionally modulated by a higher probability of C–C bond formation in the pro*S* pathway at the proximal C atom of the fumarate. The attack on this atom leads to prohibitively high energy for product radical quenching, which would further decrease the observed rate toward the *S*-product.

The analysis of values of Wigner’s tunneling correction also points out the steps that will be especially prone to the below-the-barrier tunneling acceleration. Interestingly, the highest acceleration factors were predicted for the final step of the reaction, i.e., HAT from Gly to Cys in the E:P complex (*k*_9_ TS5, 2.5–3-fold acceleration), and the quenching of the benzylsuccinyl radical intermediate (*k*_7_ TS4, 2.5-fold acceleration). As can be expected, the weakest influence of the tunnelling was predicted for a broad-barrier C–C bond formation (1.2-fold acceleration). At the same time, conformational change of Cys was not accelerated by tunnelling at all.

## Discussion

We present in this work the first detailed QM/MM modeling study of the reaction mechanism of BSS from the substrate-to the product-bound state of the enzyme. It should be underlined that our study was limited to one conformation, representing the most abundant conformational variant of the active site observed in MD simulation, which results in a lack of information on the influence of protein dynamics on the potential energy profile. While in the broad perspective, the results reported herein confirmed the previously proposed individual steps of the mechanism, we learned unexpected details on the involvement of active site residues and their movement during the catalytic cycle. In particular, the essential Cys493 needs to rotate around its C_α_-C_β_ bond to achieve conformations necessary for HAT reactions involved in generating the various radical intermediates of the mechanism. For its initial conversion into a thiyl radical (step 1), Cys493 must get close to and face the stable glycyl radical on Gly829 by assuming the C–C_α_-C_β_-S_γ_ dihedral angle of 52°. This places the thiol group of Cys493 close to the border of the active site cavity, while the glycyl radical resides at or beyond the wall of the cavity. After the thiyl radical is formed, Cys493 rotates in a clockwise orientation to reach the dihedral angle of 186°, which offers the lowest-energy barrier for abstracting an H atom from the methyl group of a bound toluene (step 2), and the only available option for adding it to fumarate with inversion of its stereochemical configuration. Interestingly, such a conformation of cysteine was recently shown in the most complete up-to-date crystal structure of BSS with both substrates bound.^31^ The subsequent C–C bond formation, by adding the generated benzyl radical to the double bond of fumarate (step 3), has to occur at the distal C atom of fumarate for a productive reaction. The following step of transferring the H atom from Cys493 back to the benzylsuccinyl radical (step 4) only works reasonably when the radical in the intermediate is positioned at the proximal C atom. If a C–C bond should be formed with the “wrong” (proximal) C atom of fumarate, we expect that this will be corrected by eliminating fumarate again from the product radical, taking advantage of the microreversibility of the partial reaction. Finally, we postulate that the product formed by HAT from Cys493 to the benzylsuccinyl radical is immediately released from the enzyme. The release of the product triggers conformational changes (also associated with changes in protein protonation), which stabilize the glycyl radical over the thiyl radical form. This conformational change also relatively decreases the barrier for the final glycyl/thiyl HAT by destabilizing the radical at Cys494 in the empty enzyme.^46^ Therefore, our model suggests that the HAT between Gly829 and Cys494 does not happen in the E:P complex, because it is too endergonic. It must be noted that we propose this explanation based on our previous investigation of HAT between Gly829 and Cys493 in the apoenzyme (based on the different structure derived from PDB 4PKF). The evolution of the enzyme structure from the model discussed here to that derived from the apoenzyme is beyond the scope of this study. Finally, during the reviewing process of this publication, a new study focused on the mechanisms of Cys493 activation has been released. The recent paper by Anas et al.^31^ suggests that the radical transfer between Gly829 and Cys493 is highly facilitated by the BSSβ accessory subunit, even in the absence of the bound substrate. Still, the authors confirmed our hypothesis that substrate binding to the active site also promotes transfer of radical between Gly829 and Cys493. As a consequence, one can expect that product release will also trigger a reverse effect. However, in order to capture the full effect described, one would need to model the reaction with both accessory subunits present, which was not possible before the advent of a new structure (PDB 9PZJ).

In addition to the movements of the amino acid side chains actively involved in the reaction, we have also observed the need for other residues in the active site to move into slightly different positions at almost every step of the catalytic cycle. The most apparent of these cases relates to the associated movement of the active site residue Tyr197, which is necessary to enable the HAT reaction between the thiyl radical and the methyl group of toluene (step 2), and probably acts to fine-tune this step of the reaction cycle, e.g., as a gating or modulating device. Interestingly, in the recently released structure of BSS, which contains both substrates in the active site, Tyr197 is oriented away from fumarate, confirming our conclusion.^31^

### Enantioselectivity

The obtained potential energy surface for the pro*S*-pathway, at first glance, contradicts our previous results from the significantly simpler QM-only analysis on the cluster model and docking-based binding energy estimation.^44^ We no longer observe any statistically significant preference for binding fumarate in the pro*R* manner over pro*S* in this study, regardless of its protonation state. The obtained reaction energy profiles no longer show a clear switch in the preference for C–C bond formation, at the proximal or distal carbon atom of the fumarate, as a function of the fumarate binding mode. However, the analysis of MD simulations for the models approximating the reaction intermediate I2 indicated a difference in the probability of an attack on the distal vs proximal atom of the fumarate bound in the pro*S* or pro*R* position. The increased chance of forming a C–C bond at the proximal atom for the pro*S*-bound fumarate, combined with a slower rate, favors the *R*-enantioselectivity of the reaction.

However, the decisive explanation of BSS enantioselectivity was provided by the calculation of the overall rate of the chemical steps of the catalytic cycle proceeding along the pro*R* or pro*S* pathways, indicating that the formation of the *R*-benzylsuccinyl radical is 43-fold faster (31-fold with tunnelling effects included) than that of the *S*-enantiomer. Those two factors together, i.e., shift in the probability of the preferential near attack conformation in function of the fumarate binding mode, and over 40-fold slower kinetics of *S*-benzylsuccinate formation along the kinetically preferred pathway a should explain the high enantioselectivity of benzylsuccinate synthase.

## Conclusions

In summary, our modeling revealed mechanistic grounds for reaction enantioselectivity and quantitatively demonstrated its kinetic origin. Furthermore, we explained the origin of H/D exchange of more than one deuteron in the product during its incubation in D_2_O, by presenting a potential pathway for nonenantioselective radical quenching. Finally, we showed that the enzyme, in its holoenzyme form, exhibits a preference for stabilizing the thiyl radical.

## Supplementary Material







## Notes

Contains details of methods such as derivation of all kinetic equations, reaction schemes and analytical procedures as well as results such as RMSD of all MD simulations, additional figures describing mechanism, results of MD analysis, tables with all data from QM calculations (energies and vibration corrections), tables with kinetic constants, chromatograms of chiral separation of reaction mixture as well as all PDB structures of stationary points used in the study (PDF)
pdb_proR (ZIP)
pdb_proS (ZIP)
