# Supplementary material for: Unravelling the Enantioselective Mechanism of Benzylsuccinate Synthase: Insights into Anaerobic Hydrocarbon Degradation through Multiscale Modeling and Microkinetics
Source: Biochemistry. 2026 Mar 3;65(6):748–63. doi: 10.1021/acs.biochem.5c00699 (PMC13298812; doi:10.1021/acs.biochem.5c00699)
Supplement: Supplementary file 1 [file bi5c00699_si_001.pdf]

Supporting information

Unravelling the Enantioselective Mechanism of Benzylsuccinate Synthase: Insights into Anaerobic Hydrocarbon Degradation Through Multiscale Modelling and Microkinetics

Maciej Szaleniec[1*], Gabriela Oleksy[1,2],Tomasz Borowski[1], Johann Heider[2,3*]

1 - Jerzy Haber Institute of Catalysis and Surface Chemistry, Polish Academy of Sciences, Kraków 30-239, Poland; email: maciej.szaleniec@ikifp.edu.pl
2 - Department of Biology, Laboratory for Microbial Biochemistry, Philipps University Marburg, 35043 Marburg, Germany; email: heider@staff.uni-marburg.de
3 - SYNMIKRO Research Center, 35043 Marburg, Germany

Index

METHODS 2
Preparation of BSS model for MD simulation 2
MM geometry minimization protocol and MD simulation protocol 2
Atom mask of the active site (clustering, RMSD of the active site) 3
Kinetic rate estimations 3
Derivation of the rate equations 5
RESULTS 9
RMSD 9
Step 1 – activation of Cys493 17
Step 2 – activation of toluene 20
Step 3 - C-C bond formation 22
Step 4 – quenching the radical benzylsuccinate intermediate 23
Step 5 – transfer of the H atom between Gly and Cys for the E:P complex 24
Reaction enantioselectivity 27
QM:MM energies 31
QM:MM – geometry of stationary states 39
Chiral analysis 42

METHODS

Preparation of BSS model for MD simulation

BSS model preparation. The initial geometry of the α subunit of BSS_{Ta} strain T1 was taken from the crystal structure (PDB codes: 5BWD, 5BWE; [1]). The β and γ subunits, as well as water molecules, were removed, and protons were added by the programs Calculate Protein Ionisation and Residue pK (Biovia Discovery Studio 4.0), assuming an optimum pH value of 7.4. Histidine residues His 53, 115, 130, 454, 609, 775, 794, 816 were protonated on the Ne atom (HIE), His 83, 164, 321, 356, 373, 499 on the Nd atom (HID), while His 36, 38, 222, 486,793, 820 were double protonated (HIP). Furthermore, Glu 334 and 425 were also protonated at Oe2 atoms (GLH). Crystallization water was removed from 5BWD due to uneven isotropic displacement of the model, resulting in its uneven representation in the structure. The reproduction of all crystallization waters in the simulations was validated in the control MD run. The resulting overall charge of the enzyme was -6. The AMBER parameters for radical Gly829 were taken from[2]. The parameters for radical Cys493 were established using Gaussian 16 (DFT B3LYP/6-31G* level of theory in a vacuum) followed by the RESP procedure as implemented in the ANTECHAMBER program from the AMBER software package[3, 4]. The initial position of toluene was taken from the position of toluene in 5BWE[1], while the position of (*R*)-benzylsuccinate from QMMM calculations was used to model its initial position in the MD simulation. The BSS-substrate models were solvated with explicit water molecules (10 Å radius around the protein, 24215 molecules) and the calculations were conducted in a periodic-boundaries box (119.6 Å x 88.0 Å x 97.7 Å). The charges of protein (-6) and substrates (-1 or -2) were balanced with the respective number of sodium ions.

Ligand parameters. The structures of the BSS ligands (fumarate, toluene, (R)-benzylsuccinate) were optimized in Gaussian 09 using DFT (B3LYP/6-31G* level of theory in a vacuum). The partial charges were obtained using Merz-Kollman electron density calculations[5] and the RESP procedure as implemented in the ANTECHAMBER program from the AMBER software package[3, 4]. The typing of ligands was achieved using the Generalised Amber Force Field (GAFF). The fumarate was considered in monoprotonated (charge -1) and deprotonated (charge -2) forms. The mol2 files with partial charges and frcmod configurational files are attached to the SI.

Molecular dynamics (MD) simulation. Classical MD simulations were performed for BSS models in complex with fumarate and toluene or its derivative using the AMBER force field [6-8]. The calculations were conducted using AMBER 18-22[7-9] employing ff03.r1 force field [10, 11]. First, the models geometry were minimized using the steepest descent (the first 500 steps) and conjugated gradient (subsequent steps) algorithms according to the following protocol:

MM geometry minimization protocol and MD simulation protocol

1. Minimization of geometry of water solvent and ions (imin=1, maxcyc=50000, ncyc=2500, ntb=1, ntr=1) with protein residue and substrates frozen with force 500 kcal

2. Minimization of geometry of water solvent and ions (imin=1, maxcyc=50000, ncyc=2500, ntb=1, ntr=1) with protein residue and substrates frozen with force 250 kcal
3. Minimization of geometry of water solvent and ions (imin=1, maxcyc=10000, ncyc=2500, ntb=1, ntr=1) with protein residue and substrates frozen with force 100 kcal
4. Minimization of geometry of water solvent and ions (imin=1, maxcyc=10000, ncyc=2500, ntb=1, ntr=1) with protein residue and substrates frozen with force 10 kcal
5. Minimization of geometry of the whole model (imin=1, maxcyc=100000, ncyc=5000, ntb=1, ntr=1) without any constarins

After the minimization of the model geometry, the MD simulation was conducted. The heating of the systems from 0 to 303 K was conducted using Langevin dynamics for 100 ps NVT, followed by 100 ps at NPV ensemble with the positional restraint of 1 kcal/mol/Å^2 on the protein's backbone and substrate heavy atoms. The production phase of MD simulations was conducted at an NPT ensemble for 45 ns at 303 K and 1 atm. The simulations were performed in periodic boundary conditions with a 2 fs time step using the SHAKE algorithm [12]. The MD simulations were used to investigate the substrate behavior in the BSS active site. The stabilities of the BSS-substrates complexes were established based on changes in the RMSD of the main chain and heavy atoms of the active site residues as well as total energy of the modeled systems.

The selection of the model for QM:MM modeling was conducted using the k-means clustering method. The stable trajectory was divided into 10 clusters among and within the most abundant cluster, describing 36% of the frames. Within this population, the frame with the closest distance of methyl substituent of toluene to Cys493 was selected as a starting point for further studies. The geometry of the frame was minimized with MM using a protocol analogous to that used before MD simulation, but reduced to 3 steps. The obtained geometry was prepared for further modeling as described in the main manuscript.

Atom mask of the active site (clustering, RMSD of the active site)

:184@CA,CB,CG,CD:192@CA,CB,CG,CZ,OH:193@CA,CB,CG,ND2:194@CA,CB,OG:323@CA,CB,OG:376@CA,CB,CG,CZ,OH:379@CA,CB,CG1,CG2,CD1:380@CA,CB,CG,CZ:386@CA,CB,CG:485@CA,CB,CG,ND2:486@CA,CB:487@CA,CB,CG:488@CA,CB,SG:489@CA,CB,CG,SD:490@CA,CB,OG:491@CA,CB,CG,CD,N:500@CA,CB,CG,ND2:503@CA,CB,CG,CD,NE:506@CA:507@CA:508@CA:509@CA,CB,OG:511@CA,CB,CG,CZ:608@CA,CB,CG,CD1,NE1,CZ2,CH2,CZ3,CE3,CD2:609@CA,CB,CG,ND1,ND2,CE1:610@CA,CB,CG,ND2:612@CA,CB,CG1,CG2,CD1:700@CA,CB,OG1,CG2:702@CA,CB,CG,CD,NE2:

Kinetic rate estimations

All kinetic constants were calculated according to a standard equation from transition state theory:

$$k_i = \left(\frac{k_B T}{h}\right) exp\left(\frac{-\Delta G^{\ddagger}}{RT}\right) \qquad \text{(eq. 1)}$$

where k_B is the Boltzmann constant, h is the Planck constant, R is the gas constant, T is 303 K, and the transmission coefficient is assumed to be unity (Table S6 and S8).

To account for the tunnelling effect that may be involved in the H atom transfer, the obtained rates were corrected by Wigner's tunnelling corrections Γ(T)[13] according to eq. 2-3:

$$k_i^T = \Gamma(T)k_i \text{ (eq. 2)}$$

$$\Gamma(T) = 1 + \frac{1}{24}\left(\frac{h\,Im(\,\nu^{\ddagger})}{k_b T}\right)^2 \quad \text{(eq. 3)}$$

where $\nu^{\ddagger}$ is the imaginary vibration frequency [Hz] associated with the transition state.

Derivation of the rate equations

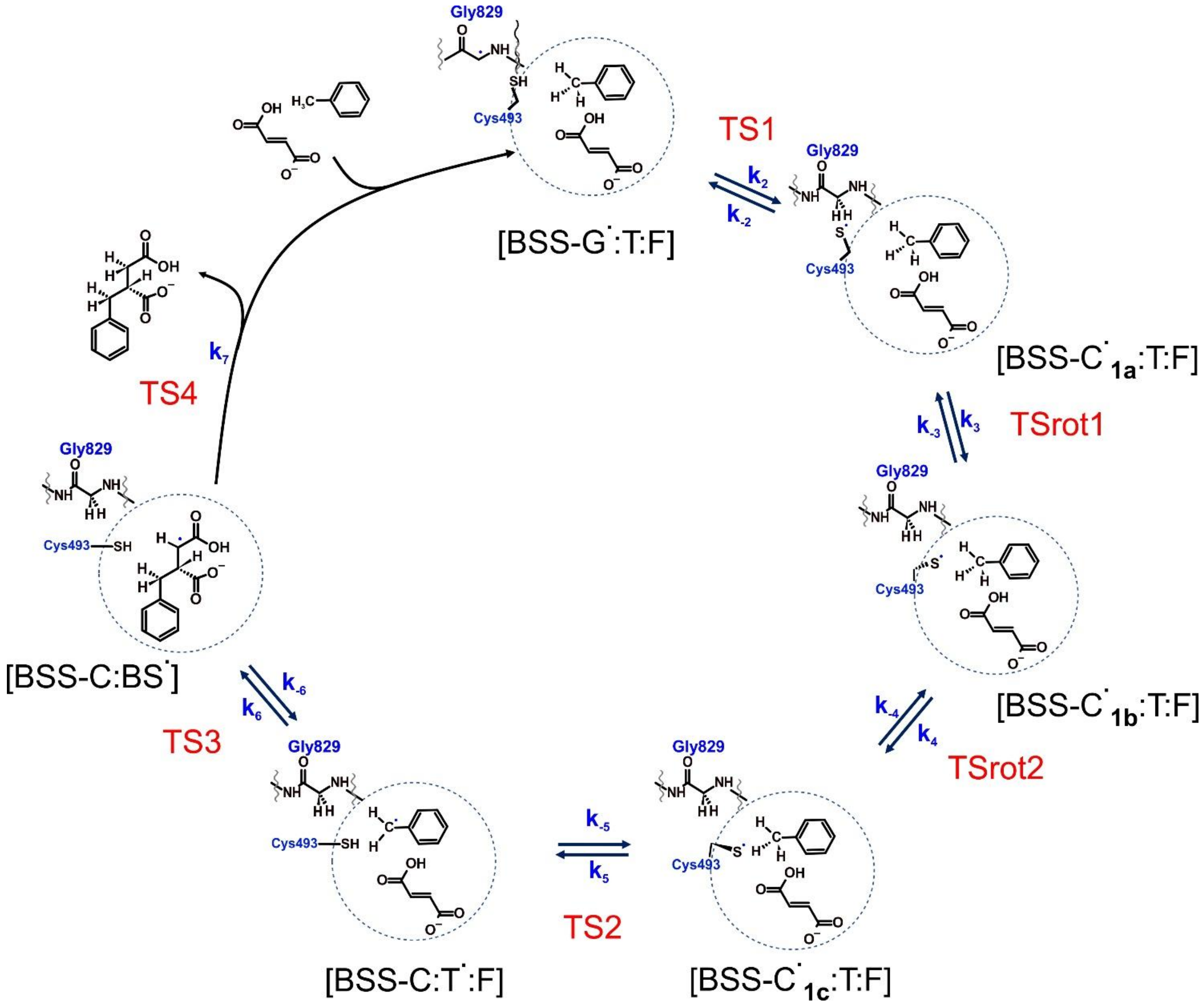

Scheme S1. The schematic representation of the whole reaction leading to the formation of benzylsuccinate, assuming the irreversibility of the benzylsuccinate radical quenching step and no kinetic limitation from substrates binding/product release steps. [BSS-G·:T:F] – BSS with glycyl radical in complex with toluene and fumarate, [BSS-$C^{\cdot}_{1a}$:T:F], [BSS-$C^{\cdot}_{1b}$:T:F], [BSS-$C^{\cdot}_{1c}$:T:F] BSS in complex with toluene and fumarate with cysteinyl radical in conformation respectively I1a, I1b, I1c , [BSS-C:T·:F] – BSS with cysteine in complex with benzyl radical and fumarate, [BSS-C:BS·] – BSS with cysteine in complex with benzylsuccinyl radical

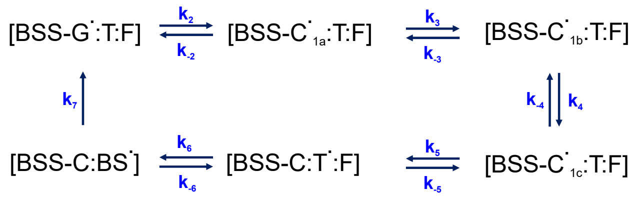

I

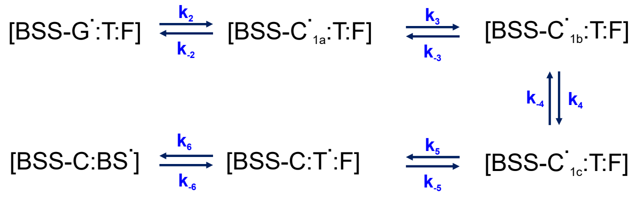

II

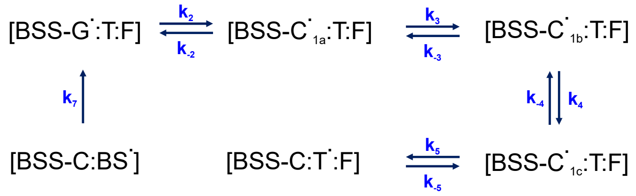

III

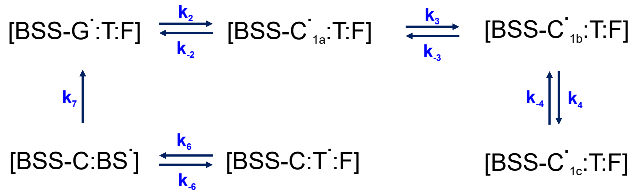

IV

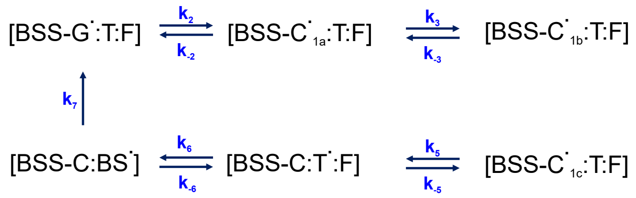

V

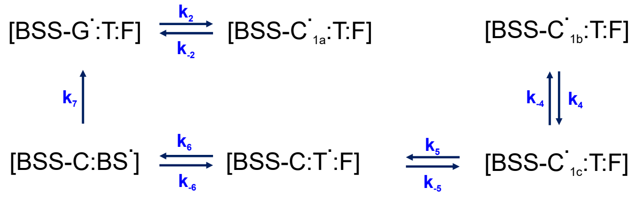

VI

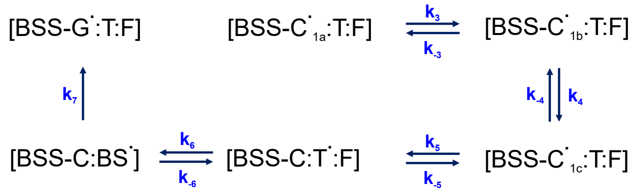

From the diagram describing internal enzyme reactions (at substrate saturation conditions) with irreversible radical quenching step (k_7) we construct four different subdiagrams (I-VI) without one transition. We construct

equations describing the relative concentration of each enzyme species by multiplying microkinetic constants leading to that species but not involving it. We add terms for each diagram and introduce it to the nominator of the equation.

Eq. S4 E:S

$$\frac{[B-G^{\cdot}\!:T\!:F]}{E_T} = \frac{k_{-2}k_{-3}k_{-4}k_6k_7 + k_{-2}k_{-3}k_5k_6k_7 + k_{-2}k_4k_5k_6k_7 + k_3k_4k_5k_6k_7 + k_7k_{-2}k_{-3}k_{-4}k_{-5} + k_{-2}k_{-3}k_{-4}k_{-5}k_{-6}}{\Delta}$$

Eq. S5 P1

$$\frac{[B-C_{1a}\!:T\!:F]}{E_T} = \frac{k_2k_6k_7k_{-3}k_{-4} + k_2k_{-3}k_5k_6k_7 + k_2k_4k_5k_6k_7 + k_2k_7k_{-3}k_{-4}k_{-5} + k_2k_{-3}k_{-4}k_{-5}k_{-6} + 0\cdot k_{-3}k_{-4}k_{-5}k_{-6}}{\Delta} = \frac{k_2k_6k_7k_{-3}k_{-4} + k_2k_{-3}k_5k_6k_7 + k_2k_4k_5k_6k_7 + k_2k_7k_{-3}k_{-4}k_{-5} + k_2k_{-3}k_{-4}k_{-5}k_{-6}}{\Delta}$$

Eq. S6 P2

$$\frac{[B-C_{1b}\!:T\!:F]}{E_T} = \frac{k_2k_3k_5k_6k_7 + k_2k_3k_6k_7k_{-4} + k_2k_3k_7k_{-4}k_{-5} + k_2k_3k_{-4}k_{-5}k_{-6} + 0\cdot k_3k_{-6}k_{-5}k_{-4} + 0\cdot k_{-2}k_{-4}k_{-5}k_{-6}}{\Delta} = \frac{k_2k_3k_5k_6k_7 + k_2k_3k_6k_7k_{-4} + k_2k_3k_7k_{-4}k_{-5} + k_2k_3k_{-4}k_{-5}k_{-6}}{\Delta}$$

Eq. S7 P3

$$\frac{[B-C_{1c}\!:T\!:F]}{E_T} = \frac{k_2k_3k_5k_6k_7 + k_2k_3k_4k_7k_{-5} + k_2k_3k_4k_{-5}k_{-6} + 0\cdot k_{-3}k_{-2}k_{-5}k_{-6} + 0\cdot k_{-2}k_{-6}k_{-5}k_4 + 0\cdot k_3k_4k_{-5}k_{-6}}{\Delta} = \frac{k_2k_3k_5k_6k_7 + k_2k_3k_4k_7k_{-5} + k_2k_3k_4k_{-5}k_{-6}}{\Delta}$$

Eq. S8 P4

$$\frac{[B-C\!:T^{\cdot}\!:F]}{E_T} = \frac{k_2k_3k_5k_6k_7 + k_2k_3k_4k_5k_{-6} + 0\cdot k_{-4}k_{-3}k_{-2}k_{-6} + 0\cdot k_{-3}k_{-2}k_{-6}k_5 + 0\cdot k_{-2}k_{-6}k_4k_5 + 0\cdot k_{-6}k_3k_4k_5}{\Delta} = \frac{k_2k_3k_5k_6k_7 + k_2k_3k_4k_5k_{-6}}{\Delta}$$

Eq. S9

$$\frac{[B-C{:}BS^{\cdot}]}{E_T} = \frac{k_2k_3k_4k_5k_6 + 0\cdot k_{-5}k_{-4}k_{-3}k_{-2} + 0\cdot k_6k_{-4}k_{-3}k_{-2} + 0\cdot k_5k_6k_{-3}k_{-2} + 0\cdot k_6k_5k_4k_{-2} + 0\cdot k_3k_4k_5k_6}{\Delta} = \frac{k_2k_3k_4k_5k_6}{\Delta}$$

As at the substrate saturation conditions, the total enzyme concentration equals the sum of the individual species we can show that Δ is a sum of all nominators of all four equations.

Eq. S10

$$\frac{[B-G^{\cdot}{:}T{:}F]}{E_T} + \frac{[B-C_{1a}^{\cdot}{:}T{:}F]}{E_T} + \frac{[B-C_{1b}^{\cdot}{:}T{:}F]}{E_T} + \frac{[B-C_{1c}^{\cdot}{:}T{:}F]}{E_T} + \frac{[B-C{:}T^{\cdot}{:}F]}{E_T} + \frac{[B-C{:}BS^{\cdot}]}{E_T} = 1$$

Eq. S11

$$\begin{aligned}\Delta = {} & k_2k_3k_4k_5k_6 + k_2k_3k_4k_5k_7 + k_2k_3k_4k_5k_{-6} + k_2k_3k_4k_6k_7 + k_2k_3k_4k_7k_{-5} + k_2k_3k_4k_{-5}k_{-6} \\ & + k_2k_3k_5k_6k_7 + k_2k_3k_6k_7k_{-4} + k_2k_3k_7k_{-4}k_{-5} + k_2k_3k_{-4}k_{-5}k_{-6} + k_2k_4k_5k_6k_7 \\ & + k_2k_5k_6k_7k_{-3} + k_2k_6k_7k_{-3}k_{-4} + k_2k_{73}k_{-3}k_{-4}k_{-5} + k_2k_{-3}k_{-4}k_{-5}k_{-6} + k_3k_4k_5k_6k_7 \\ & + k_4k_5k_6k_7k_{-2} + k_5k_6k_7k_{-2}k_{-3} + k_6k_7k_{-2}k_{-3}k_{-4} + k_7k_{-2}k_{-3}k_{-4}k_{-5} \\ & + k_{-2}k_{-3}k_{-4}k_{-5}k_{-6}\end{aligned}$$

The observed in the experiment reaction velocity is the rate of product formation. In that case (assuming a low concentration of the product) this will depend on the concentration of the enzyme before the irreversible step:

Eq. S12

$$V = k_7[BSS-C{:}BS^{\cdot}]$$

Combining Eq 7 with Eq. 4 we get:

Eq. S13

$$V = \frac{k_2k_3k_4k_5k_6k_7E_T}{\begin{gathered}k_2k_3k_4k_5k_6 + k_2k_3k_4k_5k_7 + k_2k_3k_4k_5k_{-6} + k_2k_3k_4k_6k_7 + k_2k_3k_4k_7k_{-5} + k_2k_3k_4k_{-5}k_{-6} + k_2k_3k_5k_6k_7 \\ +k_2k_3k_6k_7k_{-4} + k_2k_3k_7k_{-4}k_{-5} + k_2k_3k_{-4}k_{-5}k_{-6} + k_2k_4k_5k_6k_7 + k_2k_5k_6k_7k_{-3} + k_2k_6k_7k_{-3}k_{-4} \\ +k_2k_7k_{-3}k_{-4}k_{-5} + k_2k_{-3}k_{-4}k_{-5}k_{-6} + k_3k_4k_5k_6k_7 + k_4k_5k_6k_7k_{-2} + k_5k_6k_7k_{-2}k_{-3} + k_6k_7k_{-2}k_{-3}k_{-4} \\ +k_7k_{-2}k_{-3}k_{-4}k_{-5} + k_{-2}k_{-3}k_{-4}k_{-5}k_{-6}\end{gathered}}$$

The V/E_T value can be calculated using the kinetic constants derived from pro*R* or pro*S* reaction pathway thus enabling estimation of the relative rates leading to *R* or *S*-benzylsuccinate.

RESULTS

RMSD

Backbone | **Active site**

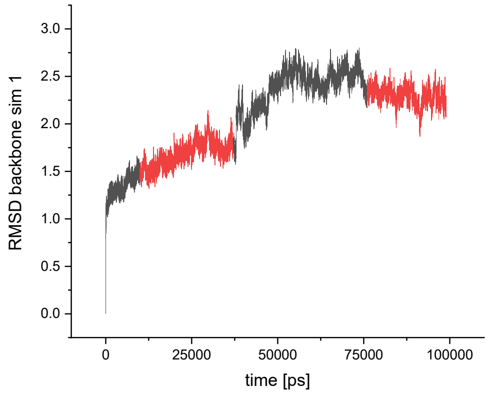

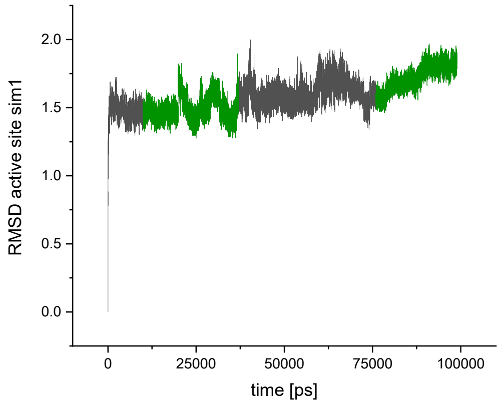

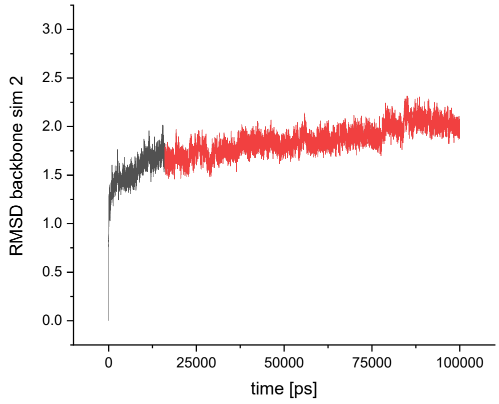

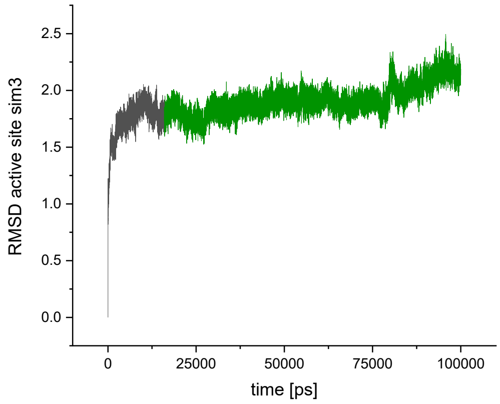

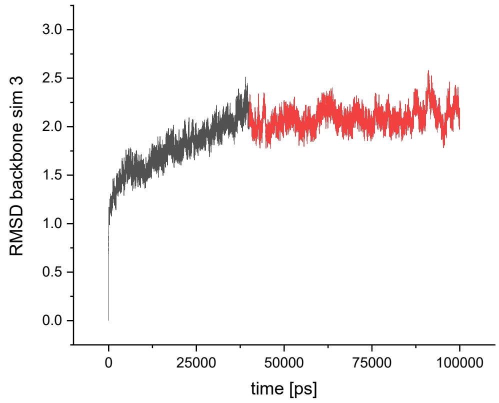

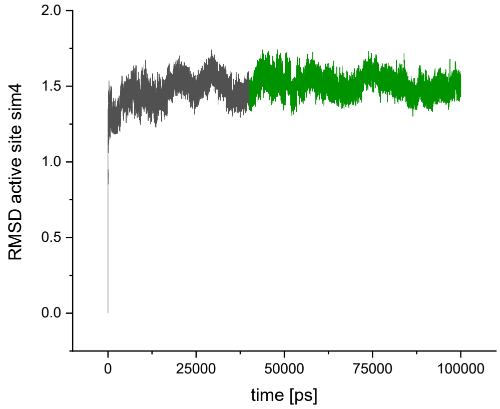

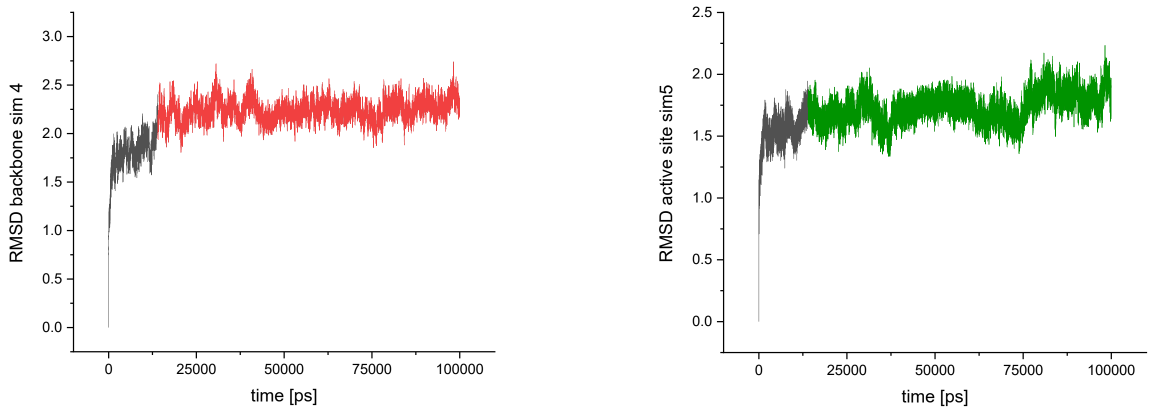

Figure S1. RMSD of the backbone (left) and heavy atoms of the active site (right) for four MD simulations with radical Gly, toluene and mono-protonated fumarate in pro*R* orientation. The stable parts of the simulations (red or green curves, respectively) were considered in statistical analysis. The clustering conducted for simulation 1 based on the RMSD of the active site.

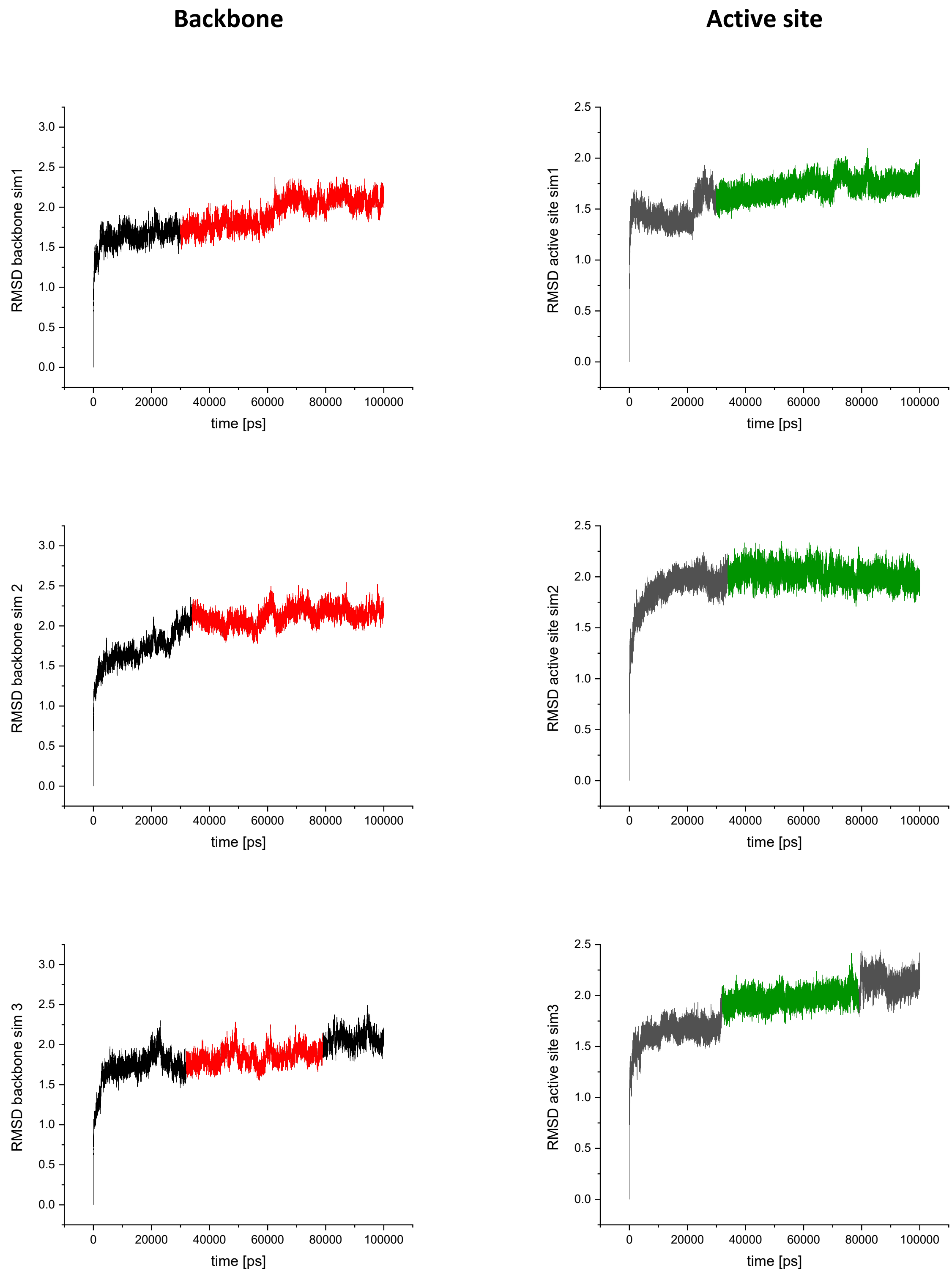

Figure S2. RMSD of the backbone for three MD simulations with radical Gly, toluene and mono-protonated fumarate in pro*S* orientation. The stable parts of the simulations (red or green curves, respectively) were considered in statistical analysis. The decision of region selected for the analysis was undertaken based on RMSD of the backbone.

Backbone **Active site**

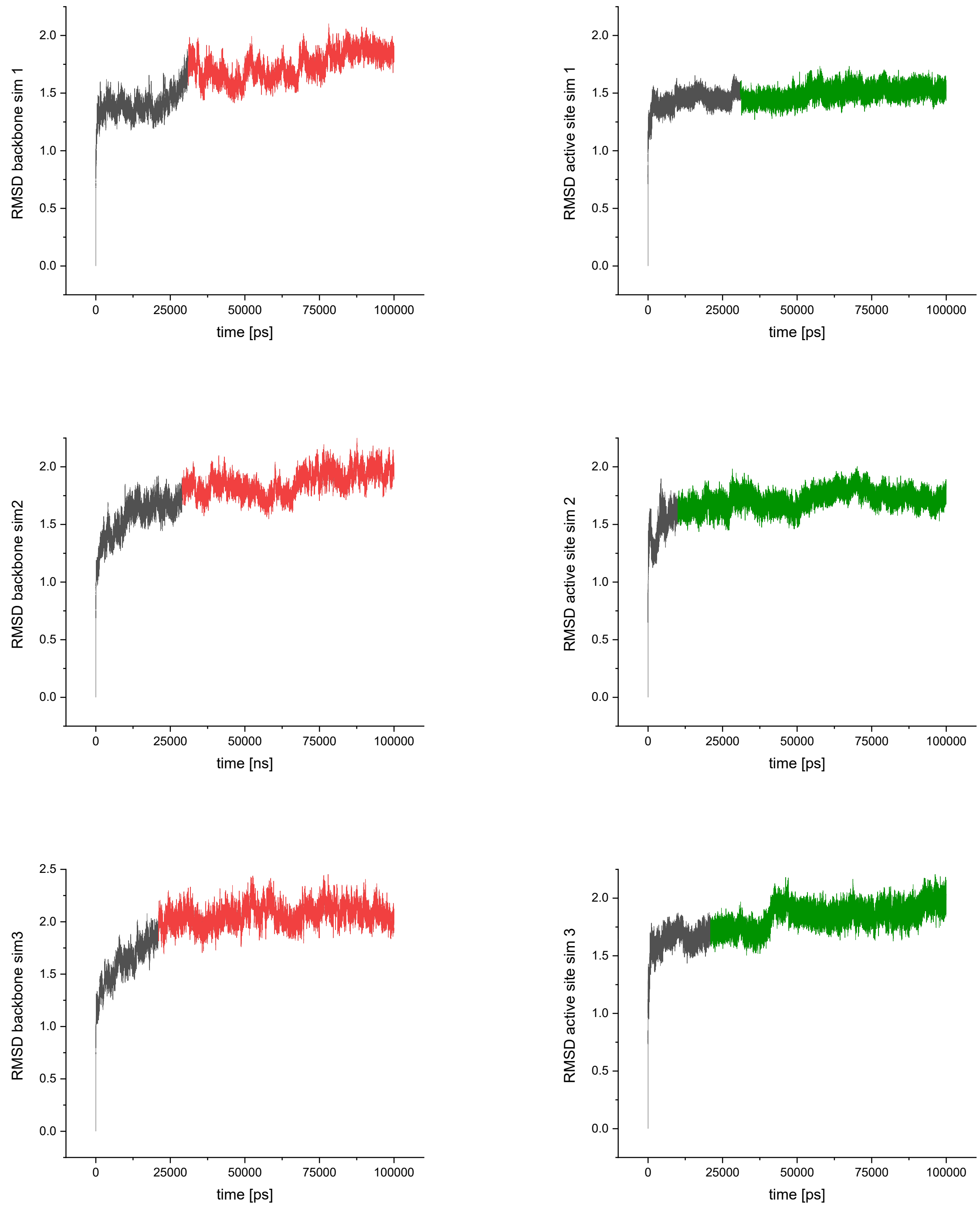

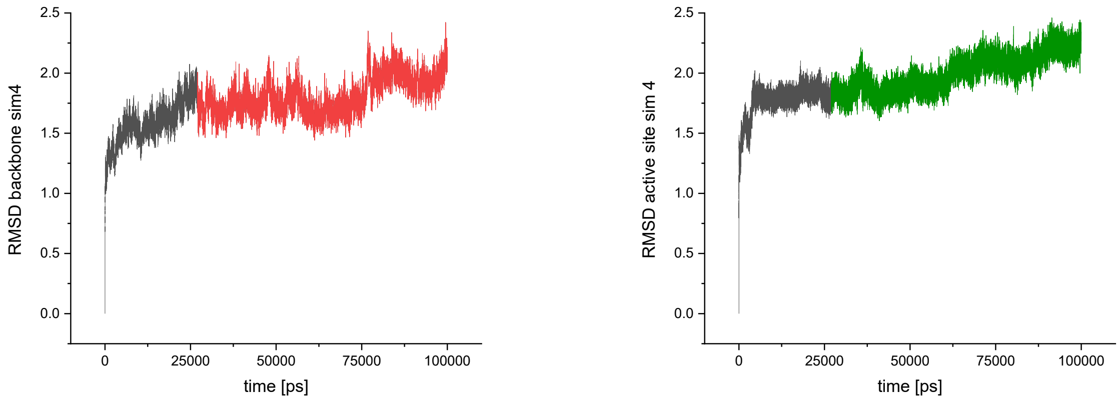

Figure S3. RMSD of the backbone for four MD simulations with radical Cys, toluene and mono-protonated fumarate. The stable parts of the simulations (red or green curves, respectively) were considered in statistical analysis. The decision of the region selected for the analysis was undertaken based on the RMSD of the backbone.

A B

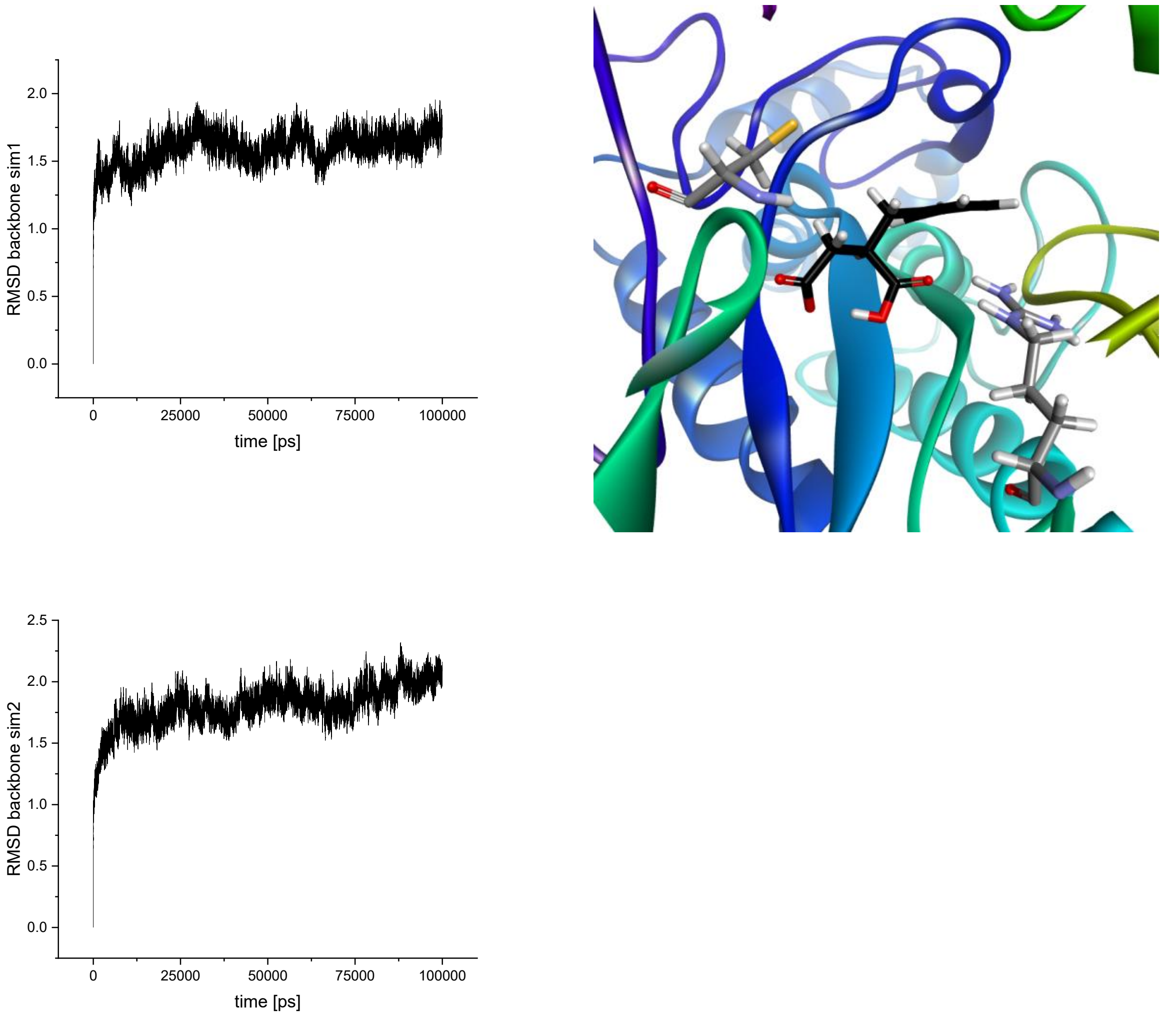

Figure S4. A) RMSD of the backbone for two MD simulations of BSS with radical Cys493 and mono-protonated benzylsuccinate (protonation at carboxyl group close to Arg508). B) figure presenting protonation mode

Backbone **Active site**

A)

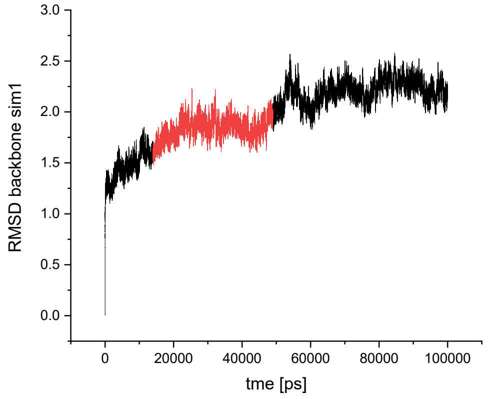

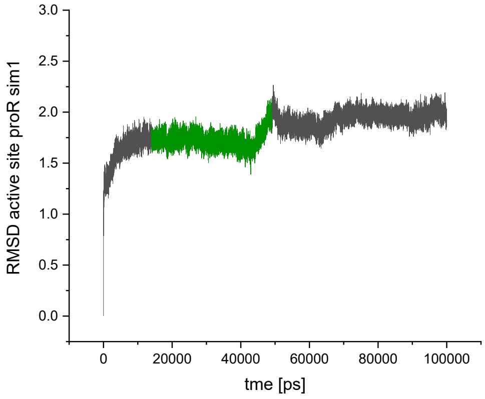

B)

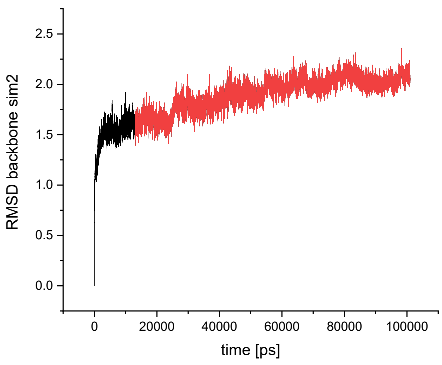

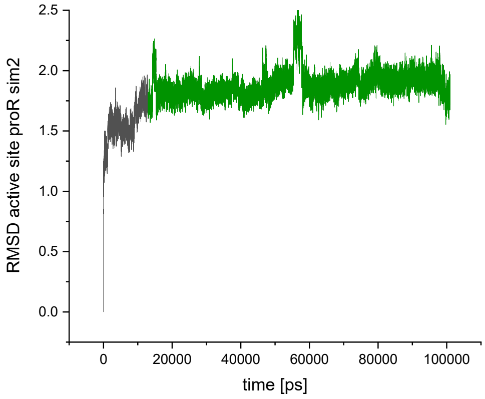

C)

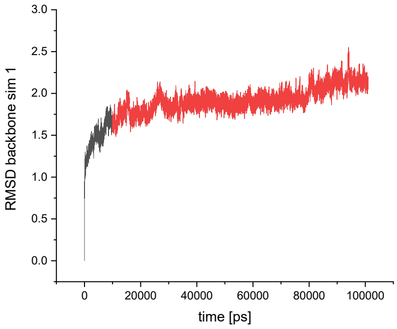

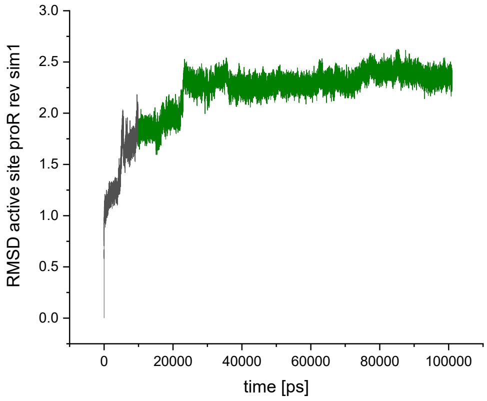

D)

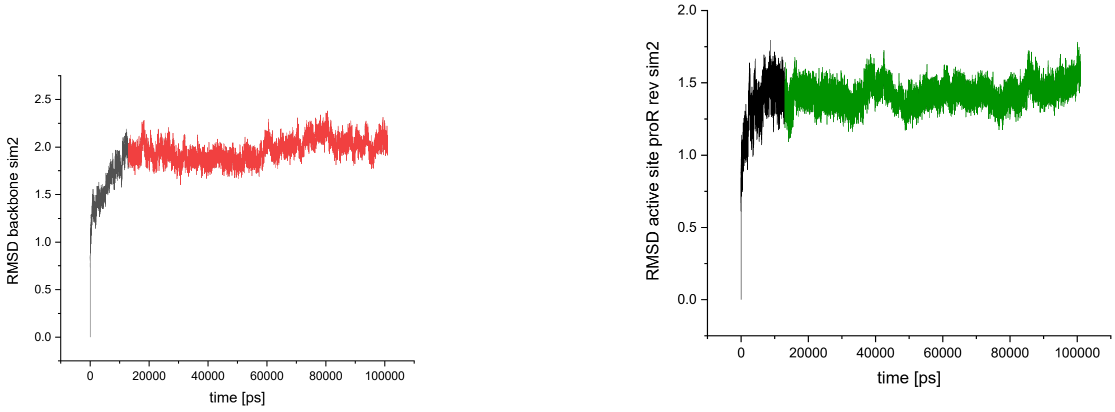

E)

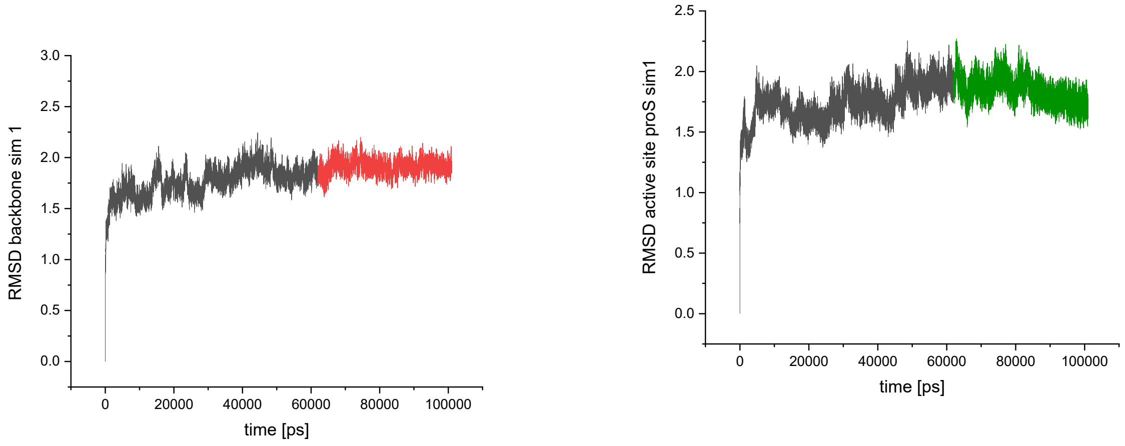

F)

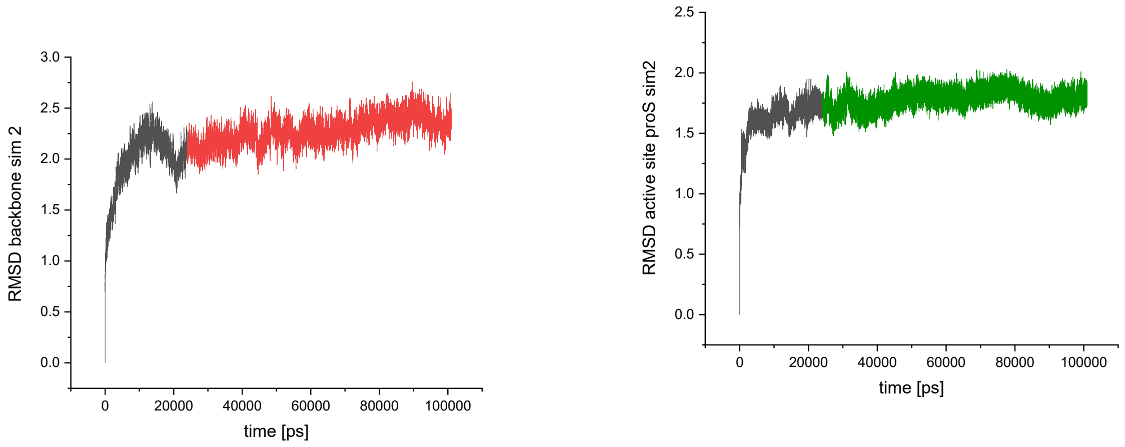

Figure S5. RMSD of the backbone for A and B) simulations with mono-protonated fumarate in pro*R* orientation, C and D) simulations with mono-protonated fumarate in pro*R* orientation with a rotated proximal carboxyl group, E and F) simulations with mono-protonated fumarate in pro*S* orientation. The stable parts of the simulations (red or green curves, respectively) were considered in the statistical analysis. The decision of the region selected for the analysis was undertaken based on the RMSD of the backbone.

Step 1 – activation of Cys493

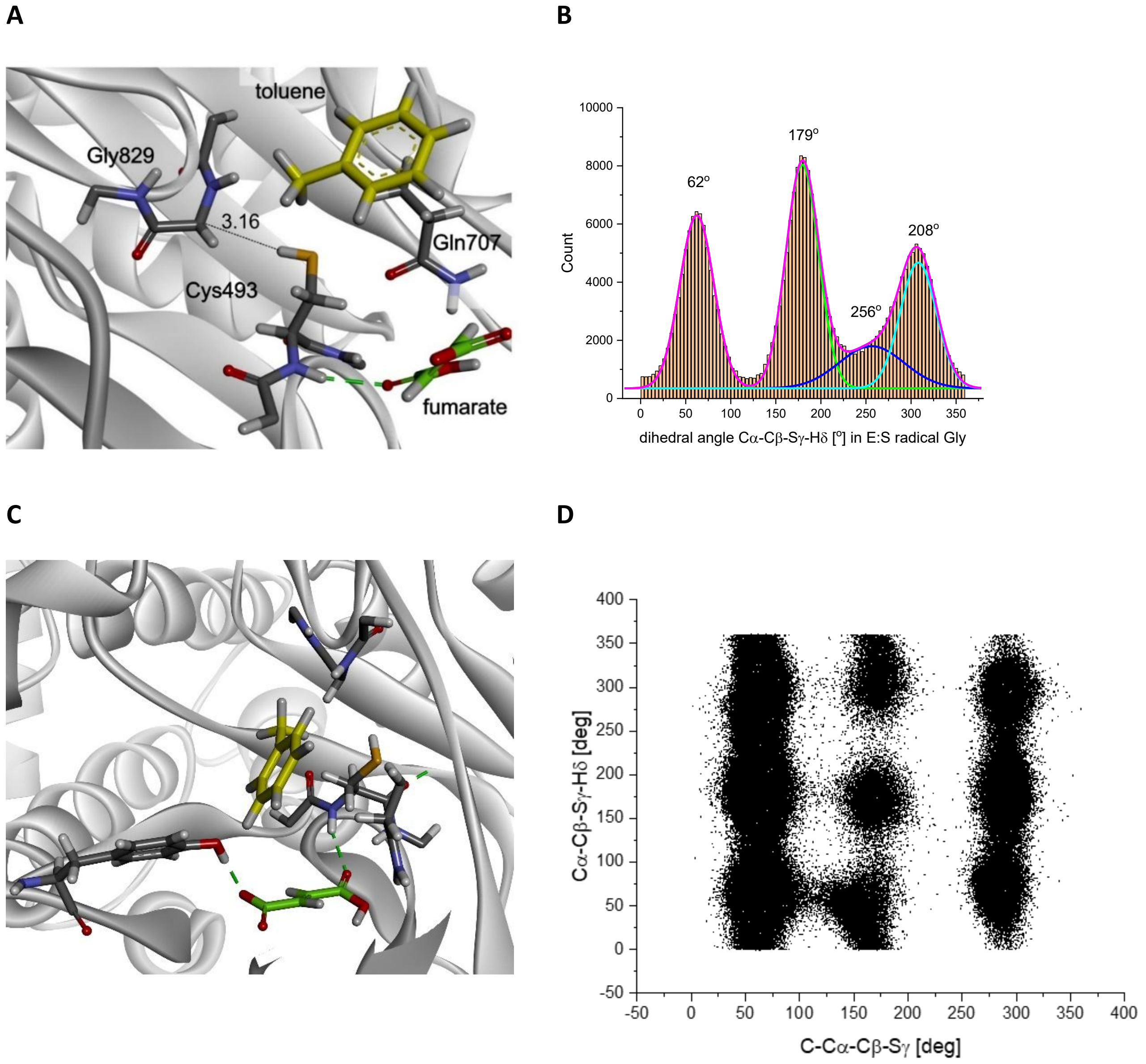

Figure S6. A) The structure of E:S_{rot} with the Cys493 SH group facing the glycyl radical after rotation around the Cβ-Sγ bond. B) The distribution of Cα-Cβ-Sγ-Hδ dihedral angle during 4 MD simulation of E:S with radical Gly; C) E:S_{rot} with Tyr197 shown forming an H-bond with fumarate D) distribution of Cα-Cβ-Sγ-Hδ dihedral angle in the function of the Cys493 conformation (C-Cα-Cβ-Sγ dihedral angle)

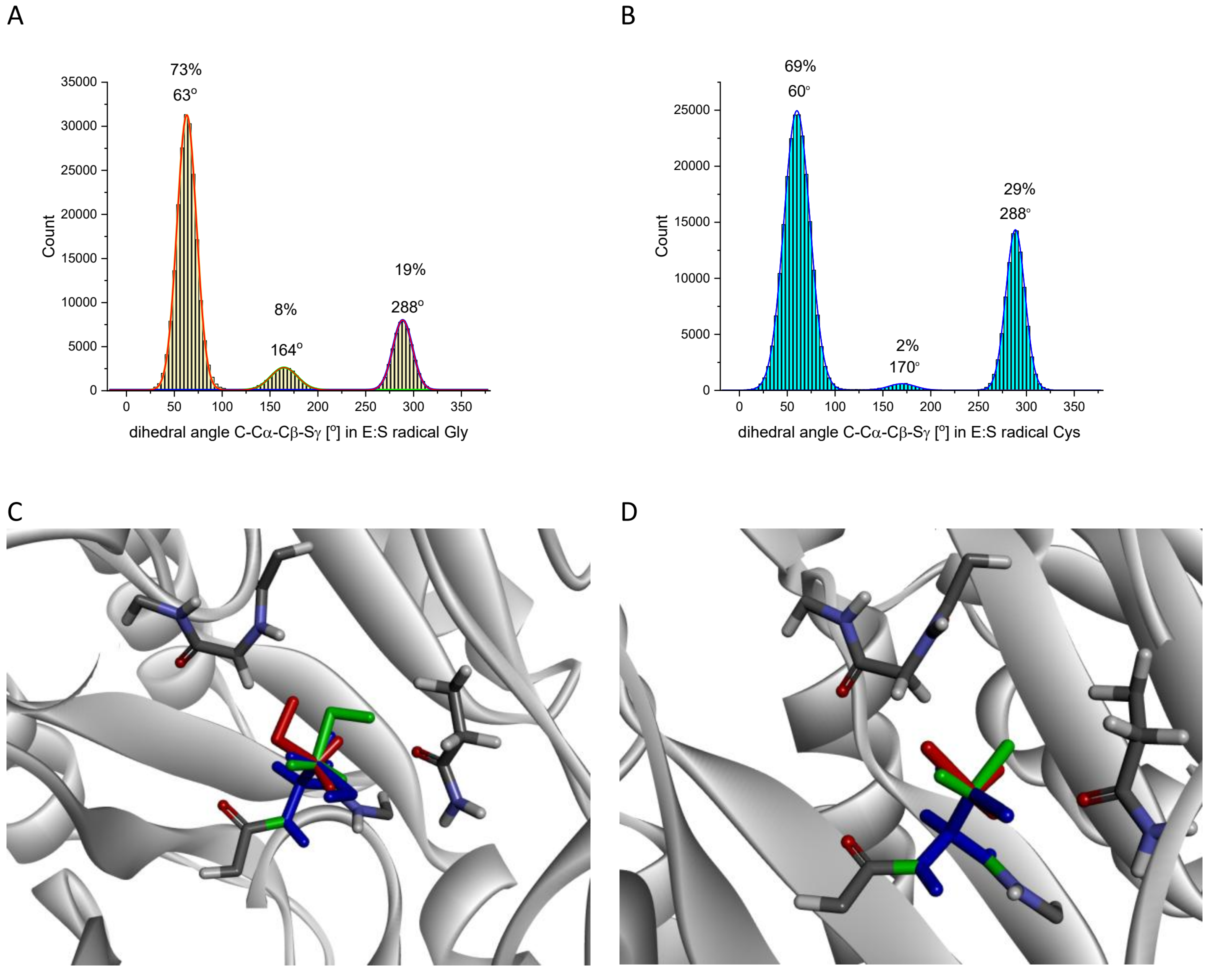

Figure S7. Distribution of the C-Cα-Cβ-Sγ dihedral angle in Cys493 for A) 4 MD simulation of E:S with radical Gly829, B) 4 MD simulation of E:S with radical Cys493; Geometry representing C) 3 conformation populations of Cys493: 63° green, 164° red, 288° blue; D) 3 conformation populations of radical Cys493: 60° green, 170° red, 288° blue

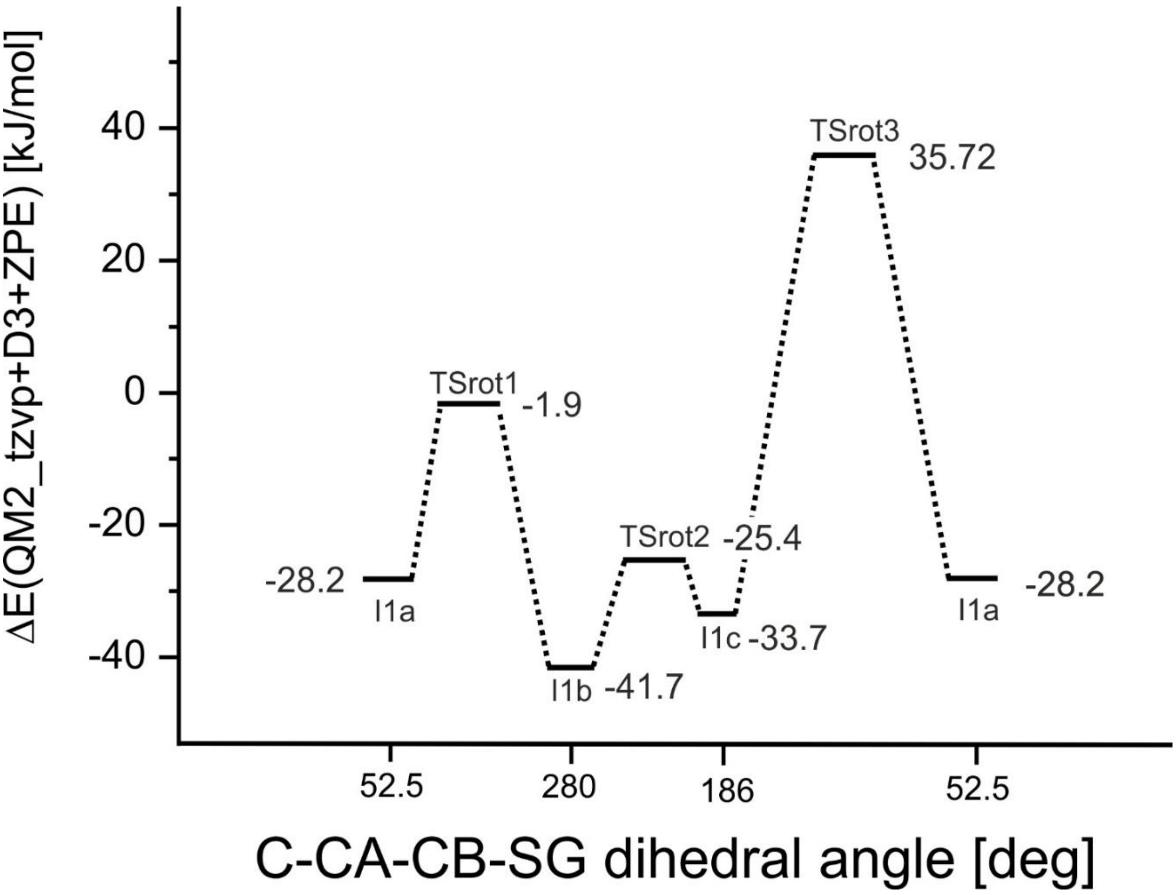

Figure S8.The rotational analysis for radical Cys493 calculated at the highest theory level. Note that the geometry of TSrot3 was obtained only for loose convergence criteria.

Step 2 – activation of toluene

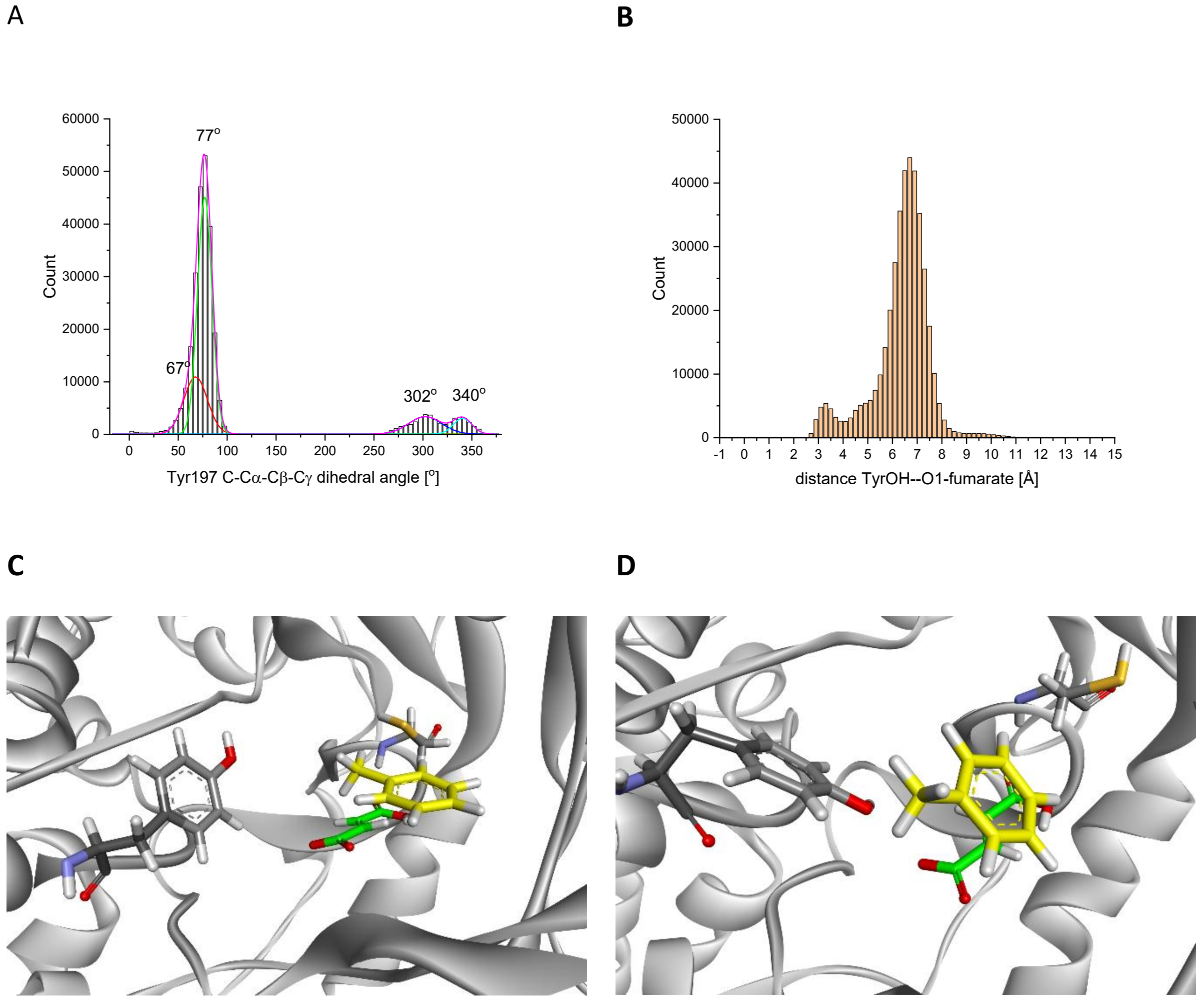

Figure S9. The analysis of Tyr197 during four MD simulations: A) distribution of C-Cα-Cβ-Cγ dihedral angle of Tyr, B) distribution of the distance between Tyr OH group and O1 atom of fumarate; C) example of Tyr197 conformation at C-Cα-Cβ-Cγ dihedral angle of 67° and D) example of Tyr197 conformation at C-Cα-Cβ-Cγ dihedral angle of 332°

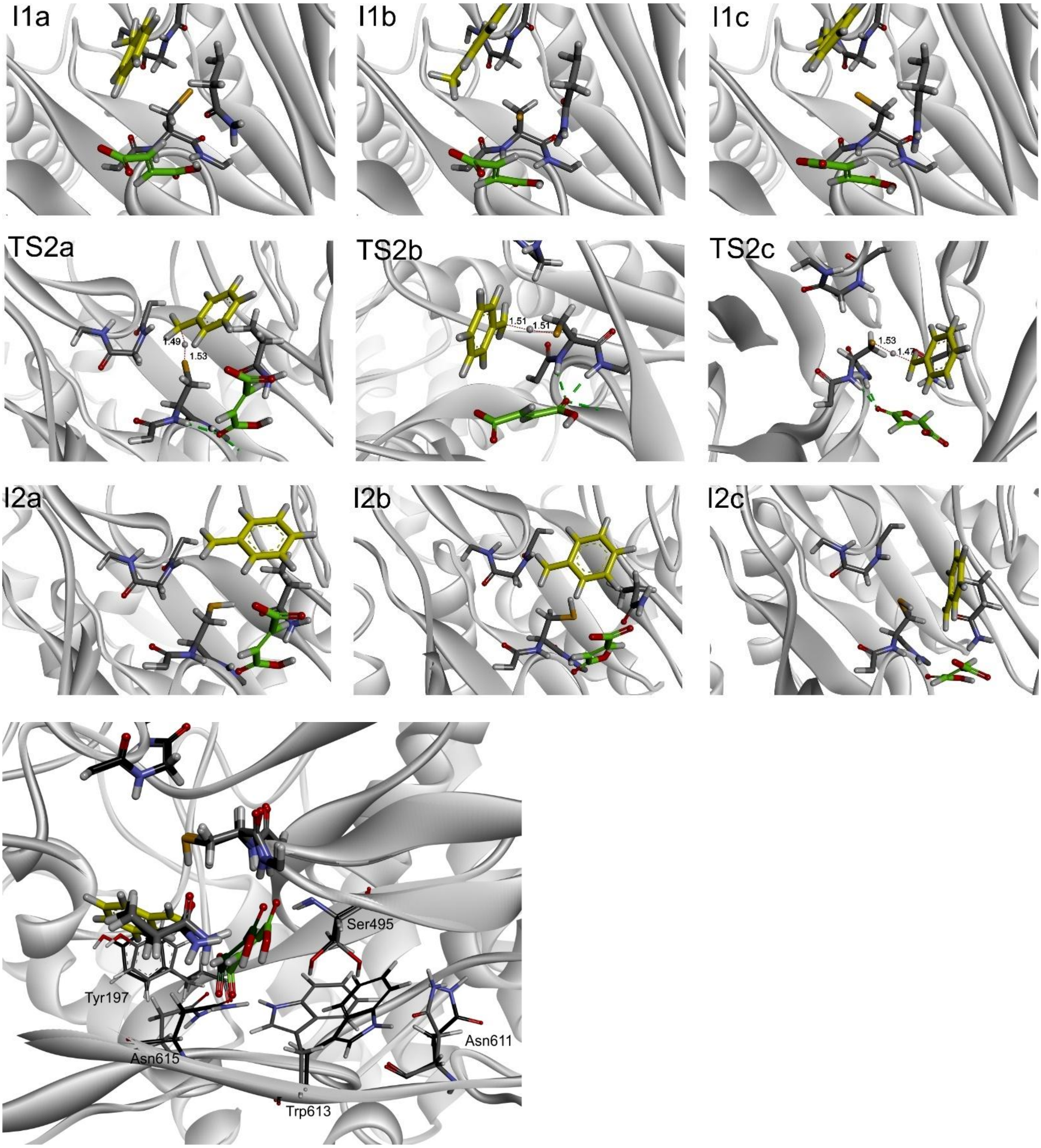

Figure S10. The geometries of toluene activation in all three variants, depending on the conformation of Cys493 and the conformational difference between I2c derived from TS2 (grey sticks) and I2c derived from TS3 (black sticks).

Step 3 - C-C bond formation

Distal C2

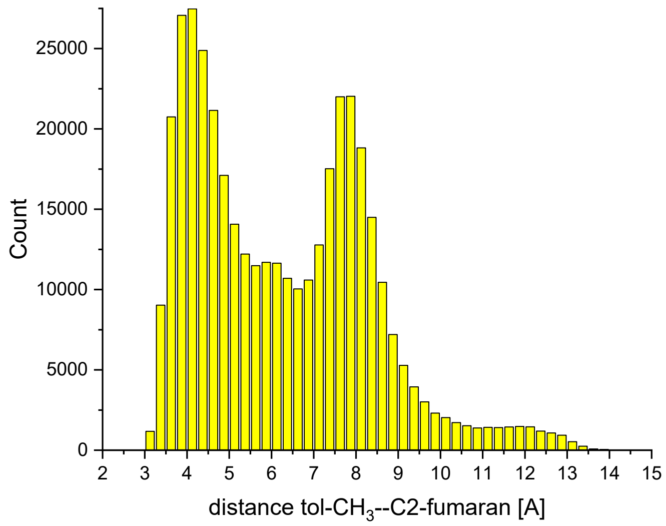

N frames	Mean [Å]	Median [Å]	Min [Å]	Max [Å]
398928	6.27	6.03	2.87	14.55

Proximal C3

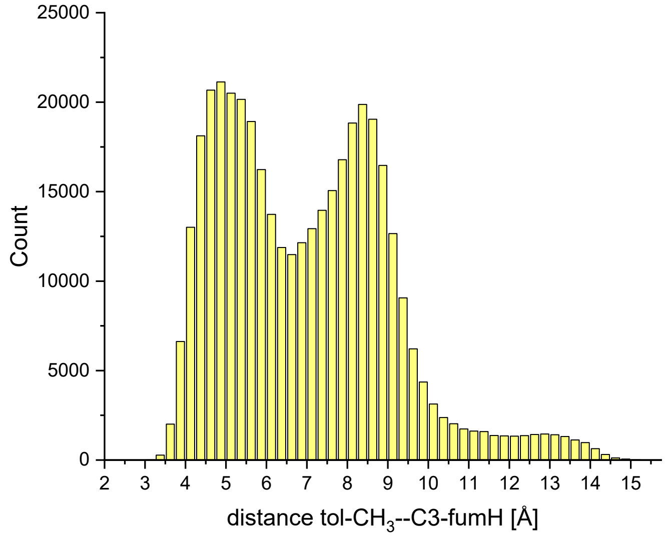

N frames	Mean [Å]	Median [Å]	Min [Å]	Max [Å]
398928	7.00	6.85	3.17	15.66

Figure S11. Distances between distal (C2) and proximal(C3) atoms of the fumarate double bond and benzyl C atom of toluene during 4 independent 100 ns MD simulations of pro*R*-bound monoprotonated fumarate.

Step 4 – quenching the radical benzylsuccinate intermediate

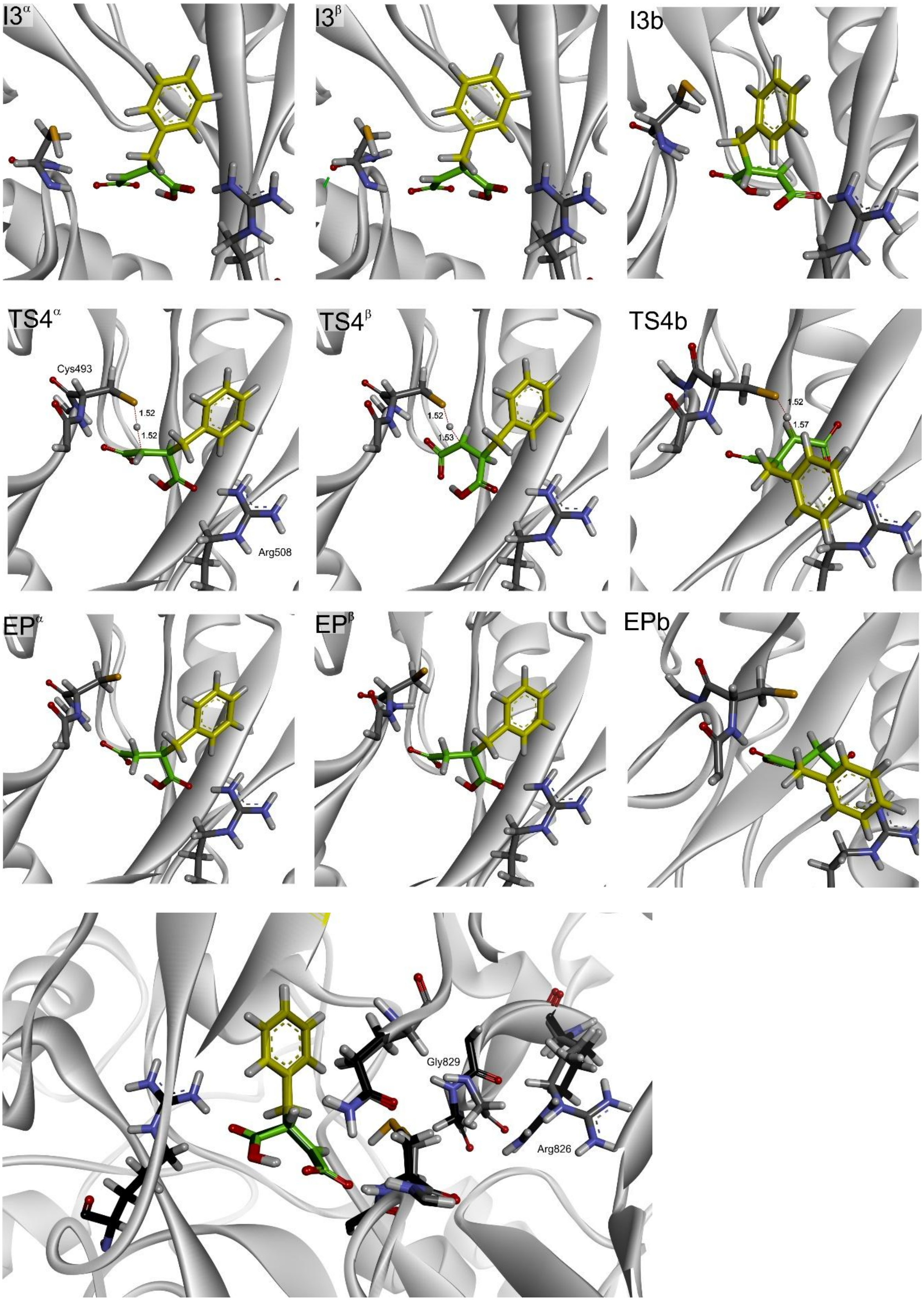

Figure S12. The geometries of benzylsuccinate radical quenching by HAT from Cys493 and the conformational difference between I3 derived from TS3 (grey sticks) and I3 derived from TS4 (dark sticks).

Step 5 – transfer of the H atom between Gly and Cys for the E:P complex.

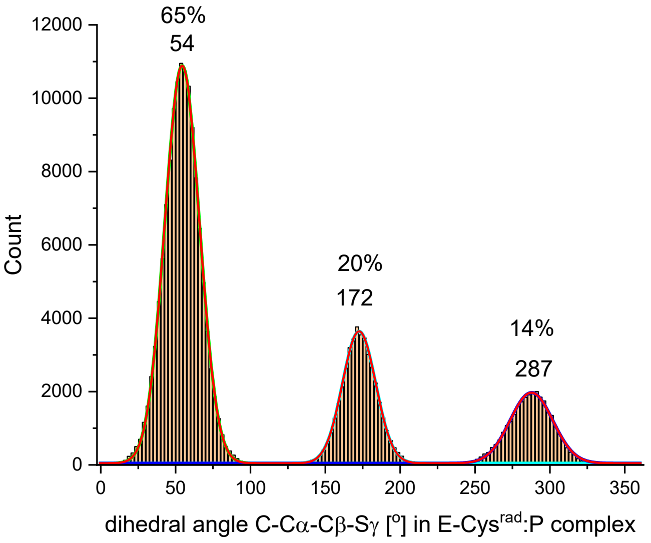

Figure S13. Distribution of dihedral angle C-Cα-Cβ-Cγ in radical Cys493 in two independent MD simulations conducted for E:P (fumarate mono-protonated on carboxyl group closer to Arg508).

A)

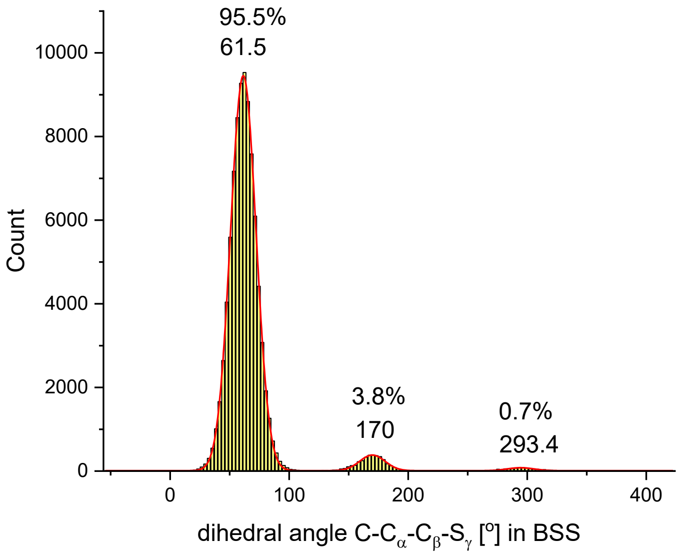

B)

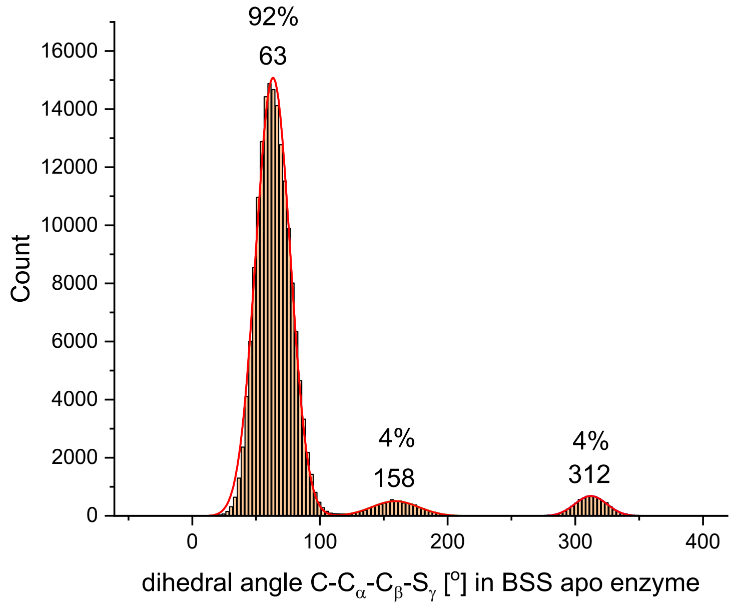

Figure S14. Distribution of dihedral angle C-Cα-Cβ-Cγ in radical Cys493 in: A) one independent MD simulation conducted with BSS model from this work and without reagents, B) two independent MD simulations conducted for apo protein model (4PKF) used in[14].

A)

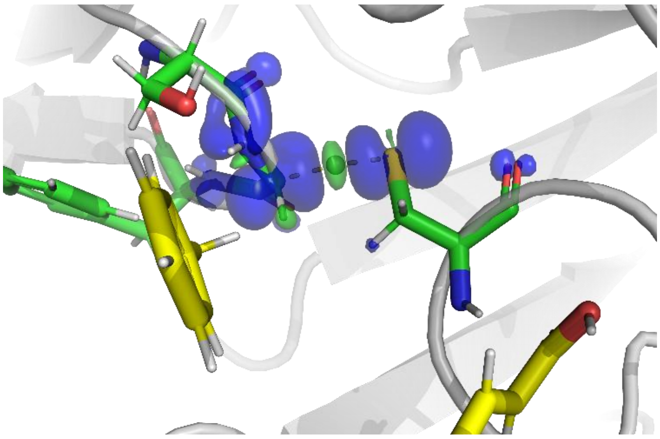

B)

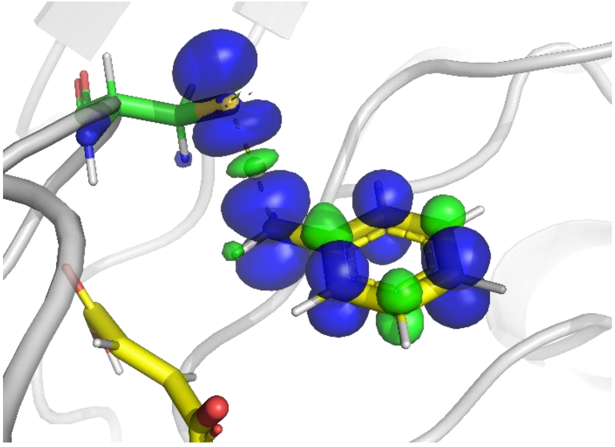

C)

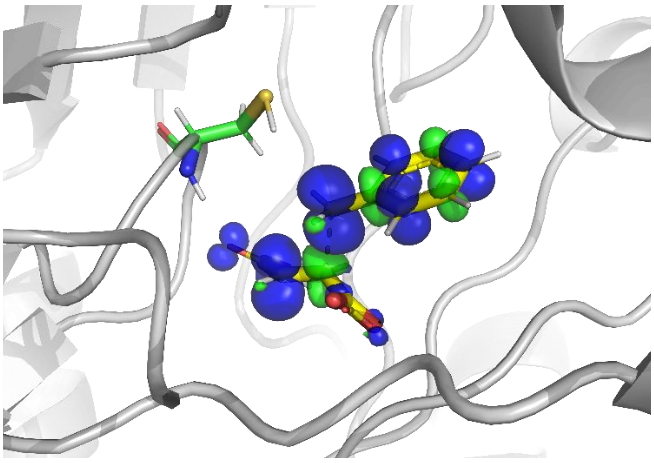

D)

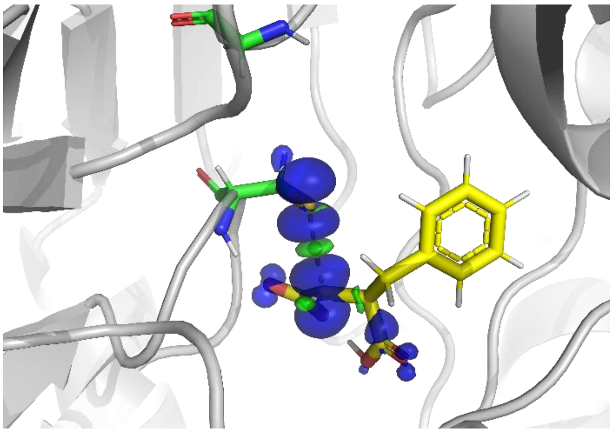

E)

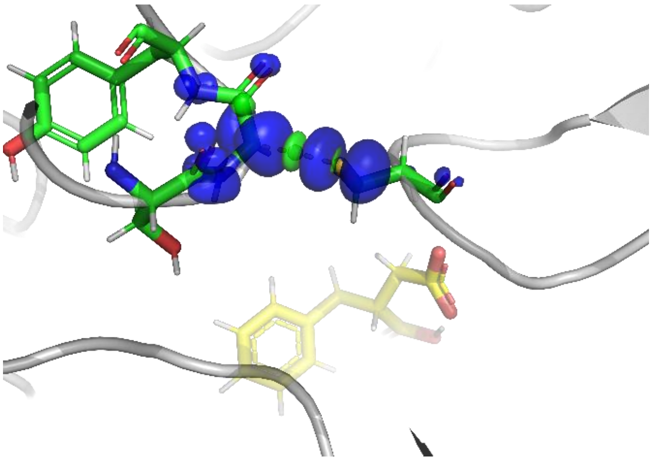

Figure S15. Spin density isosurface visualised for all transition states of the main proR pathway: A) TS1, B) TS2, C) TS3, D) TS4, E) TS5

Reaction enantioselectivity

A

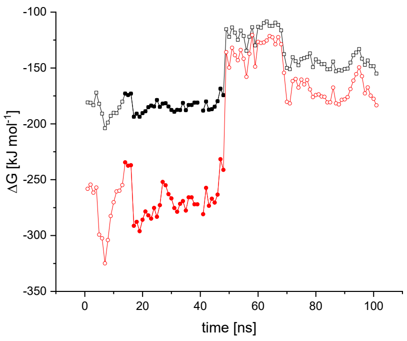

B

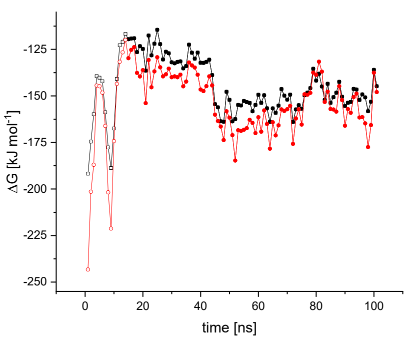

C

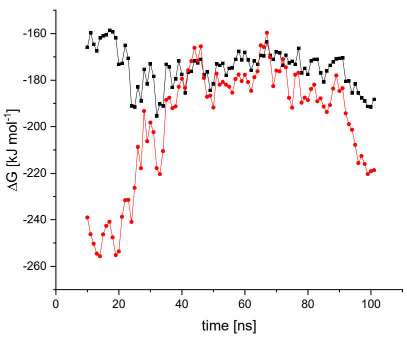

D

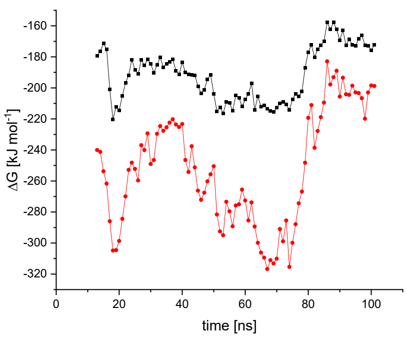

E

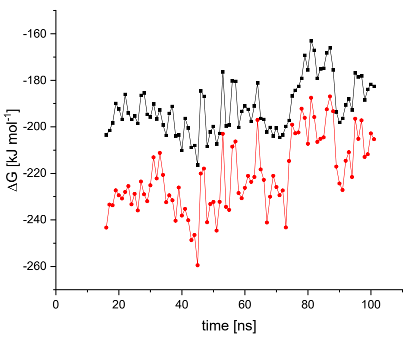

F

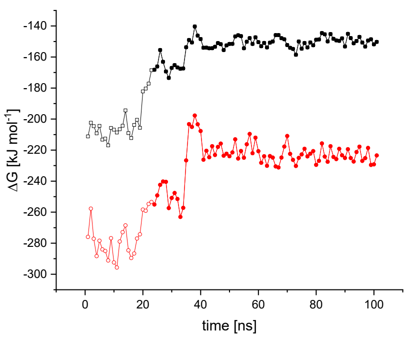

Figure S16. The results of MM/PBSA ΔG calculations from MD simulations of BSS:mono-protonated fumarate: A-B) pro*R* bound fumarate, C-D) pro*R* fumarate with a rotated protonated carboxyl group, E-F) pro*S* bound fumarate. Black line – Generalized Born, Red line – Poisson-Boltzmann, full symbol – points selected for the analysis

Distal C2

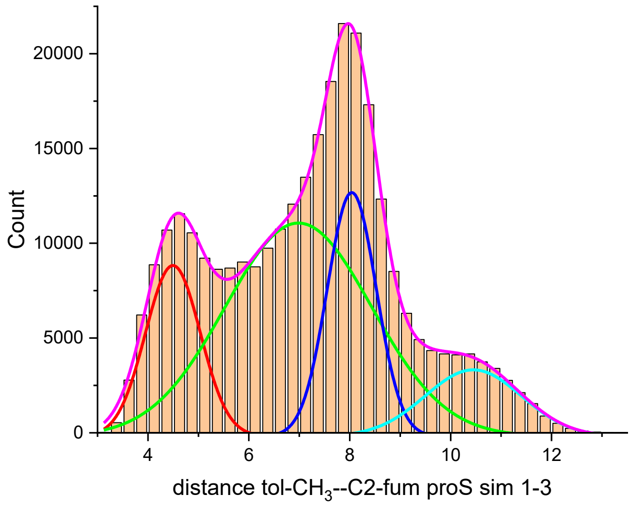

N total	Mean [Å]	Median [Å]	Min [Å]	Max [Å]
300000	7.18	7.39	3.13	14.27

Proximal C3

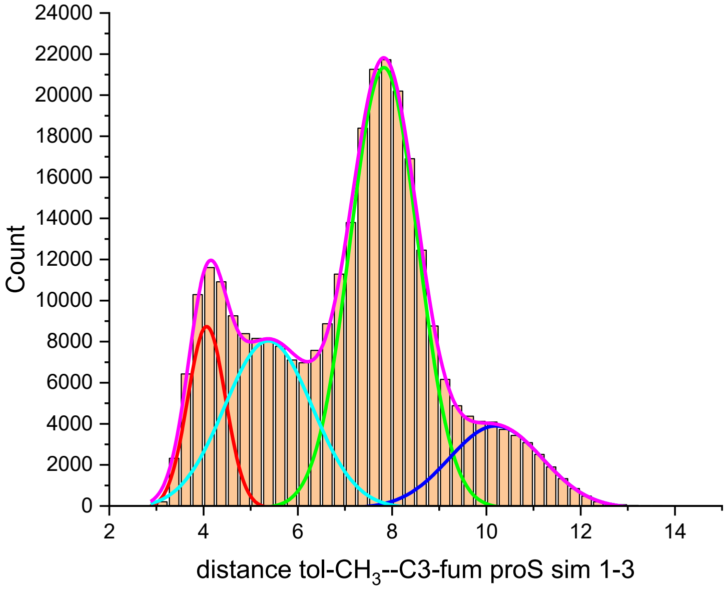

N total	Mean [Å]	Median [Å]	Min [Å]	Max [Å]
300000	7.09	7.41	3.02	14.46

Figure S17. Distances between the distal (C2) and proximal(C3) atoms of the fumarate double bond and the benzyl C atom of toluene during 3 independent 100 ns MD simulations of pro*S*-bound monoprotonated fumarate.

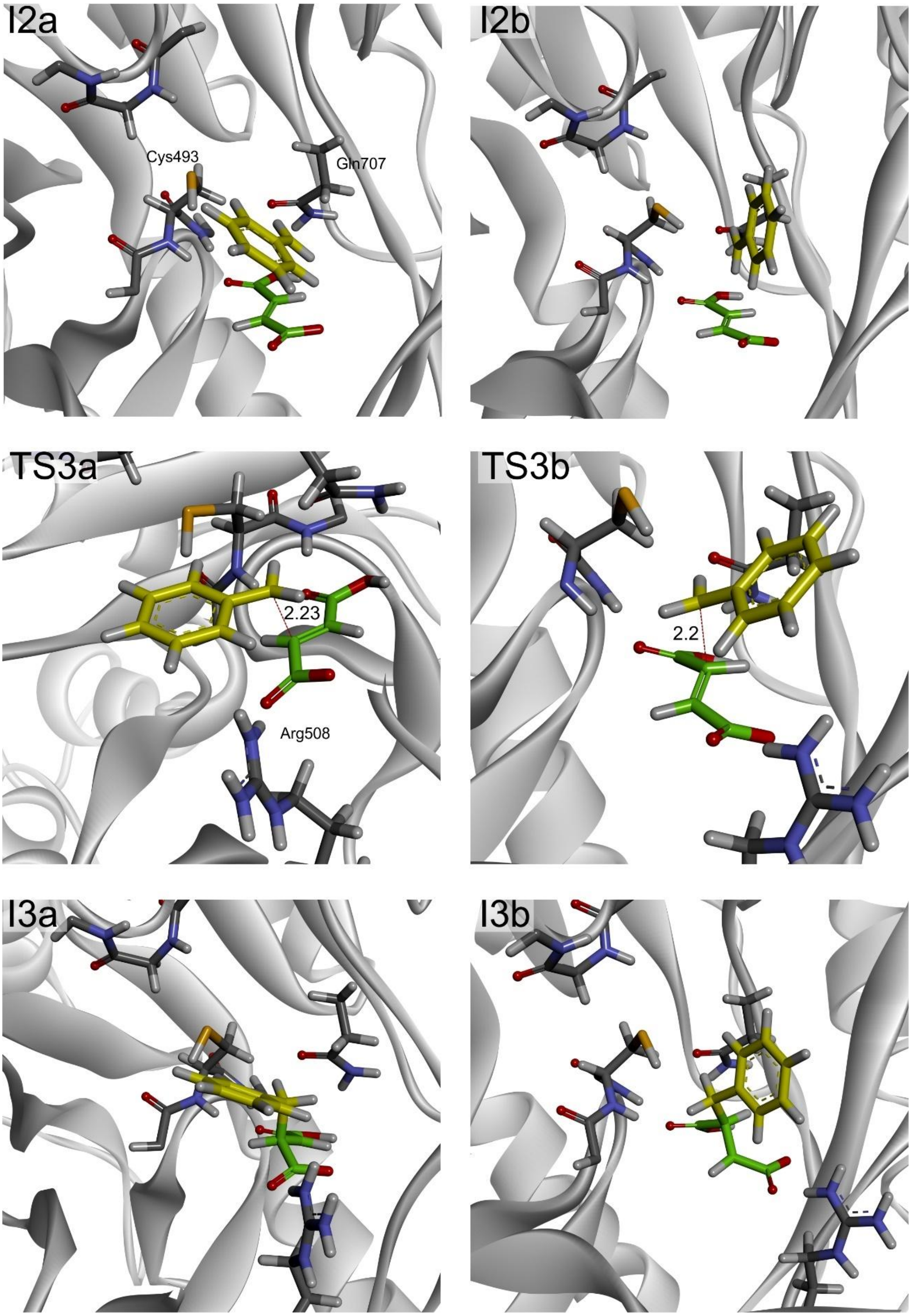

Figure S18. C-C bond formation for the pro*S*-bound fumarate: a) C-C bond formed between benzyl radical and distal C3 atom of fumarate, b) C-C bond formed between benzyl radical and proximal C2 atom of fumarate

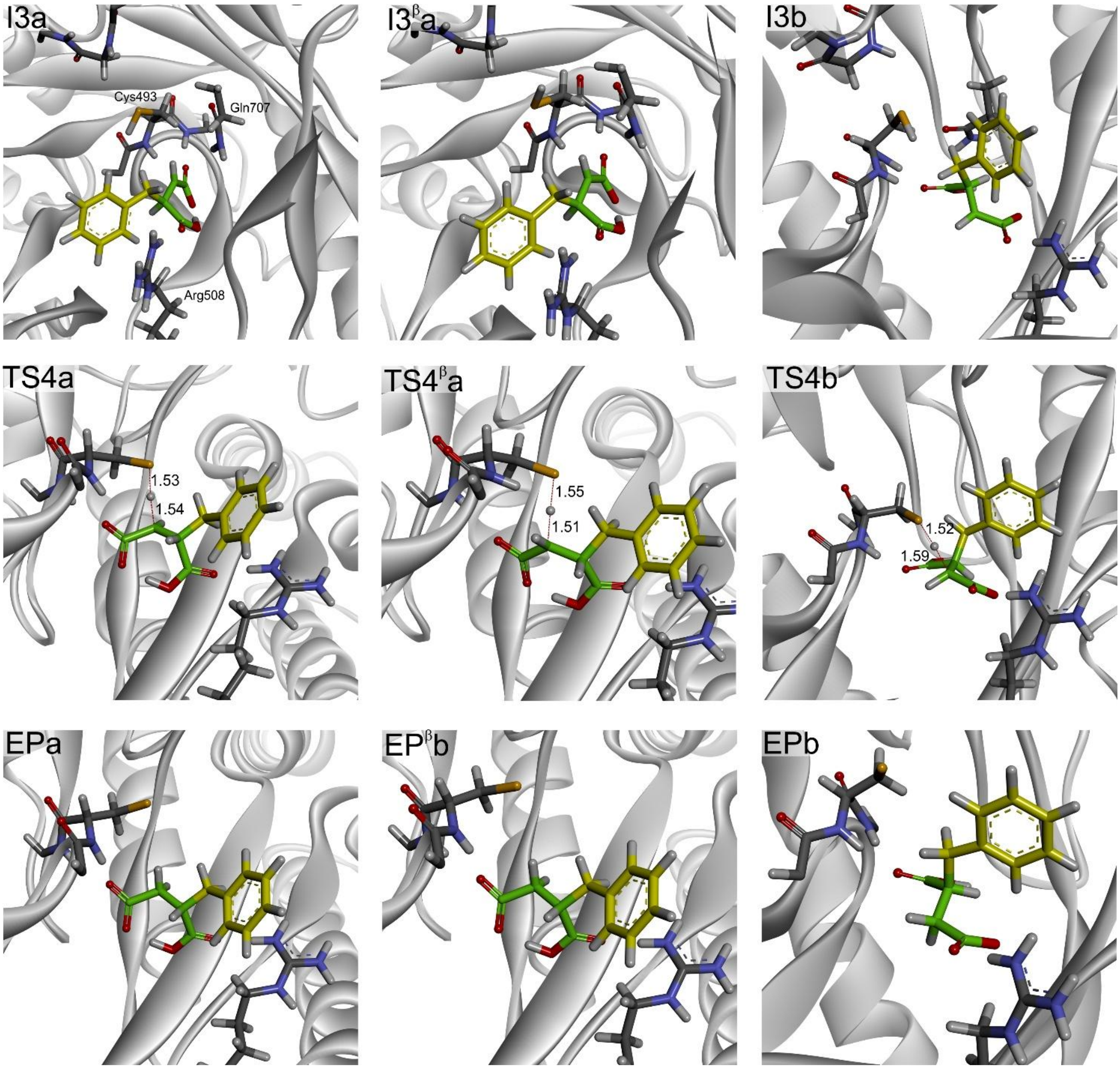

Figure S19. *S*-benzyl succinate radical quenching by Cys493: a) syn HAT from Cys to proximal C2 atom, a$^{\beta}$) anti HAT from Cys to proximal C2 atom, b) HAT from Cys to distal C3 atom.

QM:MM energies

Table S1. Energies and vibration corrections for the pro*R* pathway calculated at default conditions (1 atm., 273 K, no scaling factor). All units are in Hartree

[Ha]	E^{QM1} dzvp	E^{QM1} tzvp	E^{QM2} dzvp	E^{QM2} tzvp	ZPE	Thermal	H	G
**Step 1 – activation of Cys493**								
**E:S**	-2365.55824493968	-2366.20961790382	-8363.75303975607	-8366.11453637253	24.536596	26.05049	26.051434	23.045644
**E:S Hrot**	-2365.55541742932	-2366.20838338787	-8363.74757941795	-8366.11196473799	24.535406	26.047769	26.048713	23.046833
**TS1**	-2365.53795032585	-2366.19090709356	-8363.73073384547	-8366.09067216491	24.535406	26.047769	26.048713	23.046833
**I1**	-2365.581937946	-2366.233018092	-8363.76789923912	-8366.13003621295	24.541351	26.054725	26.055669	23.050805
**I1a***	-2365.57973303777	-2366.23058662424	-8363.76536993320	-8366.12753031643	24.541756	26.05499	26.05593	23.052458
**TSrot1**	-2365.57348427022	-2366.22620228654	-8363.75321749086	-8366.11730157776	24.541532	26.05388	26.05482	23.053247
**I1b**	-2365.58827480461	-2366.24194966642	-8363.77331147330	-8366.13388901962	24.542981	26.05549	26.05643	23.054683
**TSrot2**	-2365.57683090189	-2366.22951329371	-8363.76295837319	-8366.12499923352	24.540303	26.05295	26.05389	23.051007
**I1c**	-2365.57827999697	-2366.23055406862	-8363.76600888837	-8366.12806032555	24.540188	26.05363	26.05457	23.049951
**TSrot3**	-2365.54929776910	-2366.20240452790	-8363.74489661294	-8366.10761723273	24.546191	26.0567	26.05765	23.060657
**Step 2 – activation of toluene**								
**I1a**	-2365.56870369175	-2366.22015505998	-8363.76724805284	-8366.12678460707	24.545021	26.057341	26.058285	23.055568
**TS2a**	-2365.53155577164	-2366.18051221079	-8363.72820225284	-8366.09061137933	24.538529	26.049914	26.050859	23.050998
**I2a**	-2365.55754977988	-2366.20869661411	-8363.75497339268	-8366.11539186589	24.54145	26.053615	26.054559	23.053912
**I1b**	-2365.56633542016	-2366.21922226430	-8363.75839686177	-8366.11502261967	24.545859	26.05733	26.058274	23.059524
**TS2b**	-2365.52889781096	-2366.17954212640	-8363.71632450491	-8366.07923348779	24.538435	26.04933	26.050274	23.05372
**I2b**	-2365.54476757033	-2366.20869661411	-8363.74006439215	-8366.10177296496	24.54129	26.053183	26.054127	23.054493
**I1c**	-2365.56646282554	-2366.21885058225	-8363.76453764535	-8366.12173925949	24.545703	26.056605	26.057549	23.060942
**TS2**	-2365.54112015292	-2366.19014262673	-8363.73779947531	-8366.09838162977	24.540468	26.051058	26.052002	23.056516
**I2c**	-2365.55023224354	-2366.20132139113	-8363.74813172313	-8366.10911066041	24.543131	26.054684	26.055628	23.056729
**Step 3 - C-C bond formation**								
**I2c**	-2365.45194508504	-2366.10068922705	-8363.65402355762	-8366.03305887327	24.547839	26.056803	26.057747	23.069128
**TS3**	-2365.42852539412	-2366.07341173620	-8363.63936883283	-8366.01487514838	24.549072	26.056749	26.057693	23.072086

**I3**	-2365.48143694255	-2366.11856395130	-8363.67678717914	-8366.04930408459	24.553187	26.059255	26.060199	23.080335
**I2b**	-2365.45084761027	-2366.10239087908	-8363.65708136116	-8366.03495042814	24.547638	26.057178	26.058122	23.067142
**TS3b**	-2365.41300912051	-2366.05928368785	-8363.61849203256	-8366.00035660046	24.550074	26.057441	26.058385	23.072397
**I3b**	-2365.45081421471	-2366.09407121021	-8363.65066529819	-8366.02959948585	24.552279	26.0595	26.060445	23.075012
**Step 4 – quenching the radical benzylsuccinate intermediate**								
**I3$^{\alpha}$a**	-2365.50700866888	-2366.14346375052	-8363.70579042290	-8366.07779821857	24.552585	26.059258	26.060202	23.07772
**TS4 $^{\alpha}$a**	-2365.48588258744	-2366.11938122718	-8363.68242424925	-8366.05242786375	24.550248	26.055587	26.056531	23.07844
**E:P$^{\alpha}$a**	-2365.52336320995	-2366.15670556527	-8363.71865395157	-8366.08816044918	24.55777	26.063373	26.064317	23.084599
**I3$^{\beta}$a**	-2365.52576542669	-2366.16263312239	-8363.73225598623	-8366.10008982158	24.555723	26.061032	26.061976	23.082117
**TS4 $^{\beta}$a**	-2365.47538833495	-2366.11195044818	-8363.68210748019	-8366.05308283158	24.549209	26.055177	26.056122	23.074296
**E:P$^{\beta}$a**	-2365.53603896205	-2366.16686854738	-8363.74803588762	-8366.11246640593	24.559458	26.06443	26.065374	23.0867
**I3b**	-2365.45081421471	-2366.09407121021	-8363.65066529819	-8366.02959948585	24.552279	26.0595	26.060445	23.075012
**TS4b**	-2365.41615080984	-2366.05948626481	-8363.63186527267	-8366.00595887116	24.549184	26.055144	26.056088	23.076993
**E:Pb**	-2365.46395102289	-2366.10700481023	-8363.67341351608	-8366.04754834518	24.554089	26.061216	26.06216	23.079125
**Step 5 – transfer of the H atom between Gly and Cys for the E:P complex.**								
**E:P_rot**	-2365.52559254147	-2366.15729078714	-8363.71785348512	-8366.08581950566	24.557753	26.063432	26.064376	23.084624
**E:P**	-2365.52384603110	-2366.15562248325	-8363.71442505103	-8366.08357385082	24.558912	26.064159	26.065103	23.087309
**TS5**	-2365.47814153330	-2366.11109418045	-8363.67055202975	-8366.03996412632	24.552674	26.057067	26.058011	23.08285
**E:P-Gly$^{\cdot}$**	-2365.49737337207	-2366.13196334355	-8363.69288270337	-8366.06449495651	24.555323	26.060797	26.061741	23.083324

- I1a obtained from TSrot1 but at the same conformation as I1

Table S2. Energies and vibration corrections for the pro*S* pathway calculated at default conditions (1 atm., 273 K, no scaling factor). All units are in Hartree.

[Ha]	E^{QM1} dzvp	E^{QM1} tzvp	E^{QM2} dzvp	E^{QM2} tzvp	ZPE	Thermal	H	G
**Step 3 - C-C bond formation**								
**I2a**	-2365.45426513554	-2366.10515765690	-8363.66279734439	-8366.04041271922	24.550016	26.058578	26.059522	23.070180
**TS3a**	-2365.44062609987	-2366.08968192676	-8363.65491684864	-8366.03077507779	24.552389	26.058983	26.059927	23.075266
**I3a**	-2365.47062824028	-2366.11851152391	-8363.68324805964	-8366.05684786764	24.553318	26.060496	26.061440	23.076421
**I2b**	-2365.46114306825	-2366.11077446835	-8363.66653417132	-8366.04206937598	24.548774	26.057541	26.058485	23.068212
**TS3b**	-2365.42980640614	-2366.07785854329	-8363.63564001947	-8366.01367091872	24.550715	26.057622	26.058566	23.074127
**I3b**	-2365.46417775924	-2366.11100758158	-8363.66854643547	-8366.04438976022	24.553698	26.060557	26.061501	23.077365
**Step 4 – quenching the radical benzylsuccinate intermediate**								
**I3a**	-2365.47823691481	-2366.11591250153	-8363.66773592451	-8366.04153502962	24.550883	26.057953	26.058897	23.074527
**TS4a**	-2365.46024728375	-2366.09580278392	-8363.64812122863	-8366.02071905051	24.548263	26.054010	26.054954	23.074894
**E:Pa**	-2365.49153293516	-2366.12733865014	-8363.67945526683	-8366.05174965579	24.554956	26.061459	26.062403	23.079102
**$I3^{\beta}a$**	-2365.48208643381	-2366.11990094550	-8363.67353819525	-8366.04760948655	24.550851	26.057994	26.058938	23.073962
**$TS4^{\beta}a$**	-2365.44107367365	-2366.07751968091	-8363.63483886080	-8366.00637384456	24.549205	26.054500	26.055445	23.077480
**$E{:}P^{\beta}a$**	-2365.49120157568	-2366.12646934420	-8363.68526126910	-8366.05718887944	24.554684	26.061274	26.062218	23.078338
**I3b**	-2365.46598837033	-2366.11215356271	-8363.68255468387	-8366.05785724590	24.555896	26.062134	26.063078	23.080288
**TS4b**	-2365.42223170851	-2366.06841117497	-8363.63311799287	-8366.00508215599	24.550389	26.056410	26.057354	23.076388
**E:Pb**	-2365.46598837033	-2366.13066424488	-8363.68774125972	-8366.06110552488	24.558061	26.064145	26.065089	23.081065

Table S3. Vibrational corrections calculated for pro*R* pathway at 303 K, 1 atm and a scaling factor of 0.9806, which were used in microkinetic analysis.

	**ZPE**	**Thermal**	**H**	**G**
**E:S**	24.060562	25.647039	25.647999	22.489731
**TS1**	24.059419	25.644356	25.645315	22.490913
**I1**	24.065249	25.651198	25.652157	22.494700
**I1a**	24.065646	25.651458	25.652417	22.496367
**TSrot1**	24.065426	25.650330	25.651290	22.497210
**I1b**	24.041121	25.627302	25.628261	22.471818
**TSrot2**	24.038476	25.624807	25.625766	22.468172
**I1c**	24.064108	25.650129	25.651089	22.493868
**I1c**	24.069516	25.652960	25.653919	22.504934
**TS2c**	24.064383	25.647535	25.648494	22.500633
**I2c**	24.066995	25.651131	25.652090	22.500720
**I2c**	24.071611	25.653174	25.654133	22.513249
**TS3**	24.072820	25.653069	25.654028	22.516258
**I3**	24.076855	25.655490	25.656450	22.524540
**I3a**	24.076265	25.655491	25.656451	22.521895
**TS4a**	24.073973	25.651857	25.652816	22.522758
**E:P**	24.081349	25.659483	25.660443	22.528735
**E:P_rot**	24.082470	25.660248	25.661208	22.531462
**TS5**	24.076352	25.653280	25.654239	22.527184
**E:P-Gly$^{\cdot}$**	24.078949	25.656978	25.657937	22.527532

Table S4. Pro*R* pathway: differences in energies calculated for QM2 at B3LYP/6-311g+(2d,2p)/GD3:AMBER level of theory without (ΔE^{QM2} tzvp) or with ZPE corrections ($\Delta E^{QM2+ZPE}$ tzvp) calculated with respect to E:S geometry; corrections – energy correction introduced to the reaction energy profile matching corresponding stationary points after conformational changes of the model; final energy profile ($\Delta E^{QM2+ZPE}$ tzvp corrected). All units are in kJ mol^{-1}.

[kJ/mol]	ΔE^{QM2} tzvp	$\Delta E^{QM2+ZPE}$ tzvp	corrections	$\Delta E^{QM2+ZPE}$ tzvp corrected
**Step 1 – activation of Cys493**				
**E:S**	0.00	0.00	0	0.00
**E:S Hrot**	14.3	3.63	0	3.63
**TS1**	58.56	59.53	0	59.53
**I1**	-39.01	-28.21	0	-28.21
**I1a***	-32.37	-20.57	-7.64	-28.21
**TSrot1**	-0.47	5.84	-7.64	-1.94
**I1b**	-53.22	-37.85	-7.64	-41.69
**TSrot2**	-26.04	-10.71	-7.64	-25.38
**I1c**	-34.05	-25.77	-7.64	-33.72
**TSrot3**	21.38	43.36	-7.64	35.72
**Step 2 – activation of toluene**				
**I1a**	-32.16	-10.04	-18.17	-28.21
**TS2a**	62.82	67.89	-18.17	49.72
**I2a**	-2.25	10.50	-18.17	-7.67
**I1b**	-1.28	23.04	-18.17	4.87
**TS2b**	92.69	97.52	-18.17	79.34
**I2b**	33.51	45.83	-18.17	27.66
**I1c**	-18.91	5.00	-18.17	-13.17
**TS2**	42.41	52.58	-18.17	34.41
**I2c**	14.25	31.40	-18.17	13.23
**Step 3 - C-C bond formation**				
**I2c**	213.92	243.44	-230.21	13.23
**TS3**	261.66	294.42	-230.21	64.21
**I3**	171.27	214.83	-230.21	-15.38
**I2b**	208.95	237.94	-224.71	13.23
**TS3b**	299.78	335.17	-224.71	110.45
**I3b**	223.00	264.18	-224.71	39.46
**Step 4 – quenching the radical benzylsuccinate intermediate**				
**I3$^{\alpha}$a**	96.46	138.44	-153.82	-15.38
**TS4 $^{\alpha}$a**	163.07	198.91	-153.82	45.09
**E:P$^{\alpha}$a**	69.25	124.84	-153.82	-28.97
**I3$^{\beta}$a**	37.93	88.15	-103.53	-15.38
**TS4 $^{\beta}$a**	161.35	194.46	-103.53	90.93
**E:P$^{\beta\alpha}$a**	5.43	65.46	-103.53	-38.07
**I3b**	223.00	264.18	-224.71	39.46
**TS4b**	285.07	318.12	-224.71	93.41

**E:Pb**	175.88	221.80	-224.71	-2.91
**Step 5 – transfer of the H atom between Gly and Cys for the E:P complex.**				
**E:P_rot**	75.40	130.94	-153.82	-22.87
**E:P**	81.29	139.88	-162.75	-22.87
**TS5**	195.79	238.00	-162.75	75.25
**E:P-Gly$^{\cdot}$**	131.38	180.55	-162.75	17.80

Table S5. Pro*S* pathway: differences in energies calculated for QM2 at B3LYP/6-311g+(2d,2p)/GD3:AMBER level of theory without (ΔE^{QM2} tzvp) or with ZPE corrections ($\Delta E^{QM2+ZPE}$ tzvp) calculated with respect to I2b geometry; corrections – energy correction introduced to the reaction energy profile matching corresponding stationary points after conformational changes of the model; final energy profile ($\Delta E^{QM2+ZPE}$ tzvp corrected); I3$^{\alpha}$a' and I3$^{\beta}$a' represent respective I3 structures with shifter proton.

[kJ/mol]	ΔE^{QM2} tzvp	$\Delta E^{QM2+ZPE}$ tzvp	corrections	$\Delta E^{QM2+ZPE}$ tzvp corrected
**Step 1 – activation of Cys493**				
**I2c**	14.25	31.40	-18.17	13.23
**Step 3 - C-C bond formation**				
**I2a**	4.35	7.61	5.62	13.23
**TS3a**	29.65	39.14	5.62	44.76
**I3a**	-38.80	-26.87	5.62	-21.25
**I2b**	0.00	0.00	13.23	13.23
**TS3b**	74.56	79.66	13.23	92.89
**I3b**	-41.45	-22.75	13.23	-9.52
**Step 4 – quenching the radical benzylsuccinate intermediate**				
**I3$^{\alpha}$a'**	1.40	6.94	5.62	12.56
**TS4 $^{\alpha}$a**	56.06	54.71	5.62	60.33
**E:P$^{\alpha}$a**	-25.42	-9.18	5.62	-3.56
**I3$^{\beta}$a'**	-14.55	-9.09	5.62	-3.47
**TS4 $^{\beta}$a**	93.72	94.85	5.62	100.47
**E:P$^{\beta}$a**	-39.70	-24.18	5.62	-18.56
**I3b**	-41.45	-22.75	13.23	-9.52
**TS4b**	97.11	101.35	13.23	114.58
**E:Pb**	-49.98	-25.60	13.23	-12.37

Table S6. Kinetic constants calculated for each step of the pro*R* reaction pathway for the highest level of theory and Gibbs Free Energy corrections calculated at 1 atm, 303 K and using 0.9806 scaling factor.

	ΔG [kJ/mol]	ν [cm^{-1}]		k [s^{-1}]	Γ(T)*k [s^{-1}]	ΔG [kJ/mol]		k [s^{-1}]	Γ(T)*k [s^{-1}]
	FORWARD					REVERSE			
**E:S**	0.0					27.6			
**TS1**	65.8	i977.9	k_2	29	55.3	93.4	k_{-2}	5.0*10^{-4}	9.5*10^{-4}
**I1**	-27.6					0.0			
**I1a**	0.0					10.8			
**TSrot1**	**29.07**	i57.3	k_3	6.15*10^{7}	6.17*10^{7}	39.87	k_{-3}	8.45*10^{5}	8.48*10^{5}
**I1b**	-10.80					0.0			
**I1b**	0.0					-2.85			
**TSrot2**	13.74	i66.3	k_4	2.7*10^{7}	2.71*10^{7}	10.89	k_{-4}	8.4*10^{10}	8.4*10^{10}
**I1c**	2.85					0.0			
**I1c**	0.0					-22.09			
**TS2c**	50.03	i1081.2	k_5	1.5*10^{4}	3.1*10^{4}	27.94	k_{-5}	9.63*10^{7}	2.0*10^{8}
**I2c**	22.09					0.0			
**I2c**	0.0					13.0			
**TS3**	55.64	i821.6	k_6	1.62*10^{3}	1.9*10^{3}	68.65	k_{-6}	9.25	10.9
**I3**	13.0					0.0			
**I3a**	0.0					-2.79			
**TS4a**	68.9	i431.6	k_7	8.45	21.4	78.1	k_{-7}	0.22	0.55
**E:P**	-9.2					0.0			
**E:P**	0.0					-39.77			
**TS5**	103.3	i429.9	k_9'	9.97*10^{-6}	1.8*10^{-5}	63.49	k_{-9}'	72	1.3*10^{2}
**E:P-Gly$^{\cdot}$**	39.8					0.0			

Table S7. Vibrational corrections calculated for toluene at 303 K, 1 atm and a scaling factor of 0.9806 for pro*S* pathway.

	**ZPE**	**Thermal**	**H**	**G**
**I2a**	24.073746	25.654890	25.655850	22.514251
**TS3a**	24.076102	25.655239	25.656198	22.519453
**I3a**	24.076983	25.656723	25.657683	22.520527
**I3a'**	24.074596	25.654234	25.655194	22.518692
**TS4a**	24.072026	25.650334	25.651293	22.519206
**E:P**	24.078590	25.657638	25.658597	22.523217

Table S8. Kinetic constants calculated for each step of the pro*S* reaction pathway for the highest level of theory and Gibbs free energy calculated at 1 atm, 303 K and using 0.9806 scaling factor.

	**ΔG [kJ/mol]**	**ν [cm^{-1}]**		**k [s^{-1}]**	**Γ(T)*k [s^{-1}]**	**ΔG [kJ/mol]**		**k [s^{-1}]**	**Γ(T)*k [s^{-1}]**
	**FORWARD**					**REVERSE**			
**I2c**	0.0					26.7			
**TS3**	38.96	i414	$^{S}k_{6}$	1.2*10^{6}	1.4*10^{6}	65.6	$^{S}k_{-6}$	30.6	35.5
**I3a**	-26.67					0.0			
**I3a**	0.0					-20.45			
**TS4a**	91.4	i1166	$^{S}k_{7}$	1.1*10^{-3}	2.5*10^{-3}	70.9	$^{S}k_{-7}$	4	8.5
**E:P**	20.4					0.0			

QM:MM – geometry of stationary states

Table S9. Geometric parameters of all stationary states of the proR pathway

**Step 1 – activation of Cys493**					
**Step**	Cys$_{493}$ (C-Cα-Cβ-Sγ) [°]	Cys$_{493}$ (Cα-Cβ-Sγ-Hδ) [°]	d(Cys$_{493}$ S-H—C^{rad}-Gly$_{829}$) [Å]	d(Cys$_{493}$S—H) [Å]	angle (Cys$_{493}$-S-H-C^{rad}-Gly$_{829}$) [°]
**E:S**	62.2	240	5.06	1.36	-
**E:S H$_{rot}$**	52.5	63.6	3.17	1.1	160
**TS1**	68.5	143	1.58	1.48	167.4
**I1**	52.5	-	1.09	3.7	109

**Rotation of radical cysteine**				
**Step**	Cys$_{493}$ (C-Cα-Cβ-Sγ)[°]	d(Cys$_{493}$ S^{rad}—HC-tol) [Å]		
**TSrot1**	-20.9	4.13		
**TSrot2**	-137	3.01		
**TSrot3**	144	2.8		

**Step 2 – activation of toluene**				
**Step**	Cys$_{493}$ (C-Cα-Cβ-Sγ)[°]	d(Cys$_{493}$ S^{rad}—HC-tol) [Å]	d(tolC—H) [Å]	angle (Cys$_{493}$-S-H-C-tol) [°]
**I1a**	51.7	3.74	1.1	168
**TS2a**	84	1.54	1.49	165.7
**I2a**	62	1.36	6.0	84
**I1b**	280	4.14	1.09	122.3
**TS2b**	294.5	1.52	1.49	173
**I2b**	299	1.35	4.08	149.5
**I1c**	187.5	2.93	1.1	150.4
**TS2c**	179.4	1.53	1.47	167.4
**I2c**	173.6	1.35	2.61	147.2

**Step 3 - C-C bond formation**			
**Step**	d(**tol-C^{meth}**—C-**fum**) [Å]	angle (**fum-C=C-C^{meth}-tol**) [°]	**dihedral fum (C1-C2-C3-C4)** [°]
**I2c**	3.72	100	-173.6
**TS3a**	2.218	102.3	-157.8
**I3a**	1.55	111.8	-82
**I2b**	5.9	85	175
**TS3b**	2.2	108	-157
**I3b**	1.56	109	-116

**Step 4 – quenching the radical benzylsuccinate intermediate**

Step	Cys_{493} (C-Cα-Cβ-Sγ)[°]	d(Cys_{493} S—H) [Å]	d(Cys_{493} S-H—C^{rad}-bs) [Å]	Angle (Cys_{493}-S-H-C^{rad}-bs) [°]
**I3$^{\alpha}$a**	172.4	1.345	4.08	110
**TS4 $^{\alpha}$a**	208	1.52	1.52	171
**E:P$^{\alpha}$a**	182	3.85	1.09	155
**I3$^{\beta}$a**	169	1.345	4.2	113
**TS4 $^{\beta}$a**	210	1.52	1.53	166.5
**E:P$^{\beta}$a**	176	5.74	1.1	76
**I3b**	169	1.34	5.43	144
**TS4b**	165	1.52	1.57	169.8
**E:Pb**	162	3.14	1.09	170

Step 5 – transfer of the H atom between Gly and Cys for the E:P complex.

Step	Cys_{493} (C-Cα-Cβ-Sγ) [°]	Cys_{493} (Cα-Cβ-Sγ-Hδ) [°]	d(Cys_{493} S-H—C^{rad}-Gly_{829}) [Å]	d(Cys_{493}S—H) [Å]	angle (Cys_{493}-S-H-C^{rad}-Gly_{829}) [°]
**E:P_rot**	52	-	1.09	3.9	87
**E:P**	54	-	1.09	3.9	87
**TS5**	71	138	1.48	1.57	166
**E:P-Gly$^{\cdot}$**	53.9	47	2.94	1.52	119.8

Table S10. Geometric parameters of all stationary states of the pro*S* pathway

**Step 3 - C-C bond formation**				
**Step**	d(**tol-C^{meth}**—C-**fum**) [Å]	angle (**fum-C=C-C^{meth}-tol**) [°]	**dihedral fum (C1-C2-C3-C4)** [°]	
**I2a**	3.64	73.5	-176	
**TS3a**	2.23	103	171	
**I3a**	1.58	110	144	
**I2b**	3.89	82	-176	
**TS3b**	2.19	109	162	
**I3b**	1.56	115	132	
**Step 4 – quenching the radical benzylsuccinate intermediate**				
**Step**	Cys_{493} (C-Cα-Cβ-Sγ)[°]	d(Cys_{493} S—H) [Å]	d(Cys_{493} S-H—C^{rad}-bs) [Å]	Angle (Cys_{493}-S-H-C^{rad}-bs) [°]
**I3$^{\alpha}$a'**	167	1.35	3.9	108
**TS4 $^{\alpha}$a**	199	1.51	1.54	172
**E:P$^{\alpha}$a**	171	3.2	1.1	166
**I3$^{\beta}$a'**	171	1.35	5.3	131
**TS4 $^{\beta}$a**	211	1.55	1.52	162
**E:P$^{\beta}$a**	187	3.3	1.1	163
**I3b**	171	1.35	5.28	131
**TS4b**	168	1.52	1.59	174
**E:Pb**	171.6	3.2	1.1	166

Chiral analysis

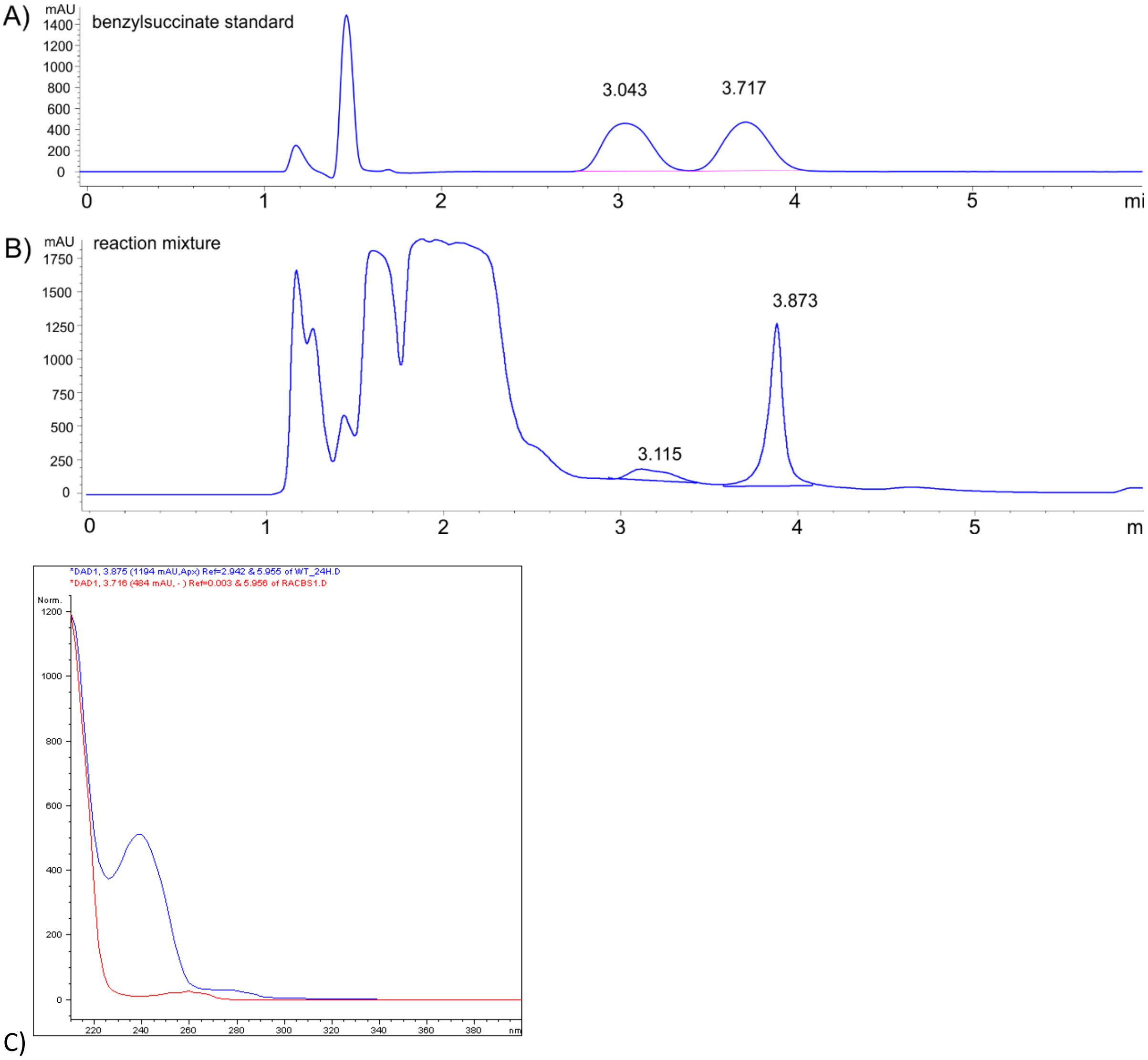

Figure S20. Chromatograms of chiral analysis conducted for the BSS reaction mixture. A) separation of *rac*-benzylsuccinate with *R* enantiomer eluting at 3.043 min and *S* enantiomer eluting at 3.72 min. B) separation of reaction mixture extract with R enantiomer eluting at 3.1 min, peak at 3.875 min is an impurity derived from cell extract with a different UV-vis spectrum from benzylsuccinate, C) UV-vis spectra – red UV-vis of standard, blue – UV-vis of impurity at 3.875 min from the reaction mixture.

Acknowledgments

The authors acknowledge the financial support provided by Deutsche Forschungsgemeinschaft/National Science Center Poland under Beethoven Life grant He2190/13-1 / 2018/31/F/NZ1/01856, as well as Polish high-performance computing infrastructure PLGrid (HPC Center: ACK Cyfronet AGH) for providing computer facilities and support within computational grant no. PLG/2023/016888, PLG/2022/016024 and PLG/2021/015218.

References

[1] Funk, M. A., Marsh, E. N., and Drennan, C. L. (2015) Substrate-bound Structures of Benzylsuccinate Synthase Reveal How Toluene Is Activated in Anaerobic Hydrocarbon Degradation, *J Biol Chem 290*, 22398-22408.

[2] Barone, V., Capecchi, G., Brunel, Y., Dheu, A. M.-L., and Subra, R. (1997) Development and validation of force-field parameters for molecular simulations of peptides and proteins containing open-shell residues, *J Comput Chem 18*, 1720-1728.

[3] Wang, J., Wang, W., Kollman, P. A., and Case, D. A. (2006) Automatic atom type and bond type perception in molecular mechanical calculations, *J. Mol. Graphics Model. 25*, 247-260.

[4] Wang, J. M., Wolf, R. M., Caldwell, J. W., Kollman, P. A., and Case, D. A. (2004) Development and testing of a general amber force field, *J. Comput. Chem. 25*, 1157-1174.

[5] Singh, U. C., and Kollman, P. A. (1984) An Approach to Computing Electrostatic Charges for Molecules, *J. Comput. Chem. 5*, 129-145.

[6] Cornell, W. D., Cieplak, P., Bayly, C. I., Gould, I. R., Merz, K. M., Ferguson, D. M., Spellmeyer, D. C., Fox, T., Caldwell, J. W., and Kollman, P. A. (1995) A Second Generation Force Field for the Simulation of Proteins, Nucleic Acids, and Organic Molecules, *J. Am. Chem. Soc. 117*, 5179-5197.

[7] Salomon-Ferrer, R., Case, D. A., and Walker, R. C. (2013) An overview of the Amber biomolecular simulation package, *Wiley Interdisciplinary Reviews: Computational Molecular Science 3*, 198-210.

[8] Pearlman, D. A., Case, D. A., Caldwell, J. W., Ross, W. S., Cheatham, T. E., DeBolt, S., Ferguson, D., Seibel, G., and Kollman, P. (1995) AMBER, a package of computer programs for applying molecular mechanics, normal mode analysis, molecular dynamics and free energy calculations to simulate the structural and energetic properties of molecules, *Comput. Phys. Commun. 91*, 1-41.

[9] Case, D. A., Cheatham, T. E., Darden, T., Gohlke, H., Luo, R., Merz, K. M., Onufriev, A., Simmerling, C., Wang, B., and Woods, R. J. (2005) The Amber biomolecular simulation programs, *J. Comput. Chem. 26*, 1668-1688.

[10] Duan, Y., Wu, C., Chowdhury, S., Lee, M. C., Xiong, G., Zhang, W., Yang, R., Cieplak, P., Luo, R., Lee, T., Caldwell, J., Wang, J., and Kollman, P. (2003) A point-charge force field for molecular mechanics simulations of proteins based on condensed-phase quantum mechanical calculations, *J Comput Chem 24*, 1999-2012.

[11] Lee, M. C., and Duan, Y. (2004) Distinguish protein decoys by Using a scoring function based on a new AMBER force field, short molecular dynamics simulations, and the generalized born solvent model, *Proteins: Structure, Function, and Bioinformatics 55*, 620-634.

[12] Ryckaert, J.-P., Ciccotti, G., and Berendsen, H. J. C. (1977) Numerical integration of the cartesian equations of motion of a system with constraints: molecular dynamics of n-alkanes, *Journal of Computational Physics 23*, 327-341.

[13] Wigner, E. P. (1932) *Z. Phys. Chem. B19*, 203.

[14] Szaleniec, M., Oleksy, G., Sekuła, A., Aleksić, I., Pietras, R., Sarewicz, M., Krämer, K., Pierik, A. J., and Heider, J. (2024) Modeling the Initiation Phase of the Catalytic Cycle in the Glycyl-Radical Enzyme Benzylsuccinate Synthase, *The Journal of Physical Chemistry B 128*, 5823-5839.
